# Physicochemical and antioxidant properties of *Apis cerana* honey from Lombok and Bali Islands

**DOI:** 10.1371/journal.pone.0301213

**Published:** 2024-04-05

**Authors:** Fahrul Huyop, Saeed Ullah, Roswanira Abdul Wahab, Nurul Huda, I. Gede Arya Sujana, Satrijo Saloko, Anak Agung Sagung Putri Risa Andriani, Nyoman Semadi Antara, Ida Bagus Wayan Gunam

**Affiliations:** 1 Faculty of Science, Department of Biosciences, Universiti Teknologi Malaysia, UTM Johor Bahru, Johor Bahru, Malaysia; 2 Department of Agro-Industrial Technology, Bioindustry Laboratory, Udayana University, Denpasar, Indonesia; 3 Faculty of Science, Department of Chemistry, Universiti Teknologi Malaysia, UTM Johor Bahru, Johor Bahru, Malaysia; 4 Faculty of Sustainable Agriculture, Universiti Malaysia Sabah, Sandakan, Sabah, Malaysia; 5 Faculty of Food Technology and Agro Industry, University of Mataram, Nusa Tenggara Barat, Indonesia; 6 Program Studi Agroteknologi, Fakultas Pertanian, Universitas Warmadewa, Denpasar, Indonesia; Monash University Malaysia, MALAYSIA

## Abstract

Limited honey production worldwide leads to higher market prices, thus making it prone to adulteration. Therefore, regular physicochemical analysis is imperative for ensuring authenticity and safety. This study describes the physicochemical and antioxidant properties of *Apis cerana* honey sourced from the islands of Lombok and Bali, showing their unique regional traits. A comparative analysis was conducted on honey samples from Lombok and Bali as well as honey variety from Malaysia. Moisture content was found slightly above 20% in raw honey samples from Lombok and Bali, adhering to the national standard (SNI 8664:2018) of not exceeding 22%. Both honey types displayed pH values within the acceptable range (3.40–6.10), ensuring favorable conditions for long-term storage. However, Lombok honey exhibited higher free acidity (78.5±2.14 meq/kg) than Bali honey (76.0±1.14 meq/kg), surpassing Codex Alimentarius recommendations (≤50 meq/kg). The ash content, reflective of inorganic mineral composition, was notably lower in Lombok (0.21±0.02 g/100) and Bali honey (0.14±0.01 g/100) compared to Tualang honey (1.3±0.02 g/100). Electric conductivity, indicative of mineral content, revealed Lombok and Bali honey with lower but comparable values than Tualang honey. Hydroxymethylfurfural (HMF) concentrations in Lombok (14.4±0.11 mg/kg) and Bali (17.6±0.25 mg/kg) were slightly elevated compared to Tualang honey (6.4±0.11 mg/kg), suggesting potential processing-related changes. Sugar analysis revealed Lombok honey with the highest sucrose content (2.39±0.01g/100g) and Bali honey with the highest total sugar content (75.21±0.11 g/100g). Both honeys exhibited lower glucose than fructose content, aligning with Codex Alimentarius guidelines. The phenolic content, flavonoids, and antioxidant activity were significantly higher in Lombok and Bali honey compared to Tualang honey, suggesting potential health benefits. Further analysis by LC-MS/MS-QTOF targeted analysis identified various flavonoids/flavanols and polyphenolic/phenolic acid compounds in Lombok and Bali honey. The study marks the importance of characterizing the unique composition of honey from different regions, ensuring quality and authenticity in the honey industry.

## Introduction

The search for high-quality honey free from human contamination and also rich in natural products is crucial to ensure that the honey is fit for consumption as this commodity is highly prone to adulteration worldwide [1,2]. *Apis cerana* is commonly found all over Indonesia, especially in the Bali and Lombok Islands [3,4]. Honey bees from this genus collect nectar from many plants on the island, including coconut and sugar palm saps, and subsequently create honey through regurgitation and digestion of the acquired nectar, which incorporates various healthy biological compounds proven to give benefits to cure gastric disturbances, skin burns, ulcers as well as its antioxidant, anti-proliferative and anti-bacterial properties [5,6].

The main types of honey produced worldwide originate from two primary categories of bees, namely, the *Apis* species, including the commonly known honeybee, and stingless bees of the *Trigona* species. Codex standards [7] for honey from *Apis* species are well established but not for stingless bee honey. Adulteration of raw honey harvested from wild sources is a main concern due to constraints in the monthly production cycle. Although honeybee cultivation is a viable strategy to boost monthly production, the production remains inadequate to meet local and global demands, leading to the soaring prices of commercial pure honey [8]. Driven by economic considerations and the scarcity of pure honey in the market, unscrupulous profiteering individuals realized that they could make considerable revenues by adulterating pure honey with cheap and readily available sweeteners before selling them at premium prices [9]. Efforts to tackle the adulteration issue have been made locally and globally. Despite these attempts, there has yet been a tangible success in reducing adulterated honey production, highlighting the ongoing challenges in implementing effective countermeasures.

It is important to note that the distinctive attributes of unexplored wild honey originating from the regions of Lombok and Bali are attributable to the diverse flora and fauna endemic to the jungle environment. This unique ecological setting harbors a plethora of medicinal properties believed to address contemporary ailments, potentially derived from the nectar in flowers or plant saps processed within the gastrointestinal tract of bees [10]. The resultant honey encapsulates these therapeutic qualities, particularly from this geographical expanse. Its richness emanates from the abundance of diverse flowering plant species and the natural vegetation indigenous to this locale, thereby augmenting the inherent benefits of the honey. The composition of honey is greatly influenced by factors such as, i) the species of bees involved, ii) the botanical origins of the floral sources, and iii) the amalgamation of environmental and processing influences, thus collectively contributing to their macro and micronutrient profile [6,11].

Honey constitutes a complex matrix of sugars comprising approximately 80% simple sugars, such as fructose and glucose, which are readily metabolized by the digestive system of bees, and an extensive array of over 200 compounds, including antioxidants, vitamins, minerals, phenolic acids, proteins, and enzymes [12]. While the specific composition of antioxidants in honey can vary according to botanical sources frequented by foraging bees across diverse geographic regions, their fundamental constituents remain relatively consistent across different honey varieties [13]. Similarly, several physicochemical or proximate analysis attributes, including pH, moisture content, hydroxymethylfurfural (HMF), and antioxidant properties, are used to assess honey quality [14]. The notable inverse correlation between pH levels and microbial presence has to do with the acidic conditions impeding the viability of unwanted microorganisms, while the low moisture content delays the fermentation processes in honey. Likewise, the HMF levels are useful in gauging honey freshness, at which high levels point to high-temperature exposure or prolonged storage durations of the honey [14]. Additionally, heightened antioxidant properties bear relevance to biomedical applications.

The adulterated honey mostly contains table sugar, sucrose syrup, or high fructose syrup, and lacks the provenance of undergoing enzymatic processes within the honey bee stomach [15,16]. Advancements in technology have further rendered fraudulent honey production increasingly sophisticated, confounding even experts in their ability to discern authenticity [9]. Hence, current data is imperative to establish a foundational understanding of honey’s unique characteristics from distinct regions, thereby facilitating the prevention of adulterated honey production and ensuring consumer well-being. Given the above circumstance, our research aims to establish a standard physicochemical profile of the *Apis cerana* honey from unreported regions of Lombok and Bali. This research essentially profiles honey collected exclusively to the Lombok and Bali islands, rich in unexplored flora- and fauna-rich jungles. We aim to fill the gaps in the physicochemical data on honey samples from the selected area in Bali and Lombok island varieties. A comparative analysis with selected Malaysian honey and the Tualang honey from Sabah on Borneo island and Manuka [16,17], was also done to serve as a reference for understanding different profiles of honey varieties from the above-said Indonesian islands.

## Material and methods

### Honey sample collection

A Letter of Research Permit (LRP) with reference number 620/SIP/IV/FR/10/2023 was obtained from the National Research and Innovation Agency (BRIN-Badan Riset & Inovasi Nasional), Jakarta, Indonesia, at the start of this study. This permit authorized the research activities and sample collection in the forest reserve. Additionally, the collection of honey samples on private land/properties and the precise locations are properly documented in the following section of this study.

**Fig 1:**
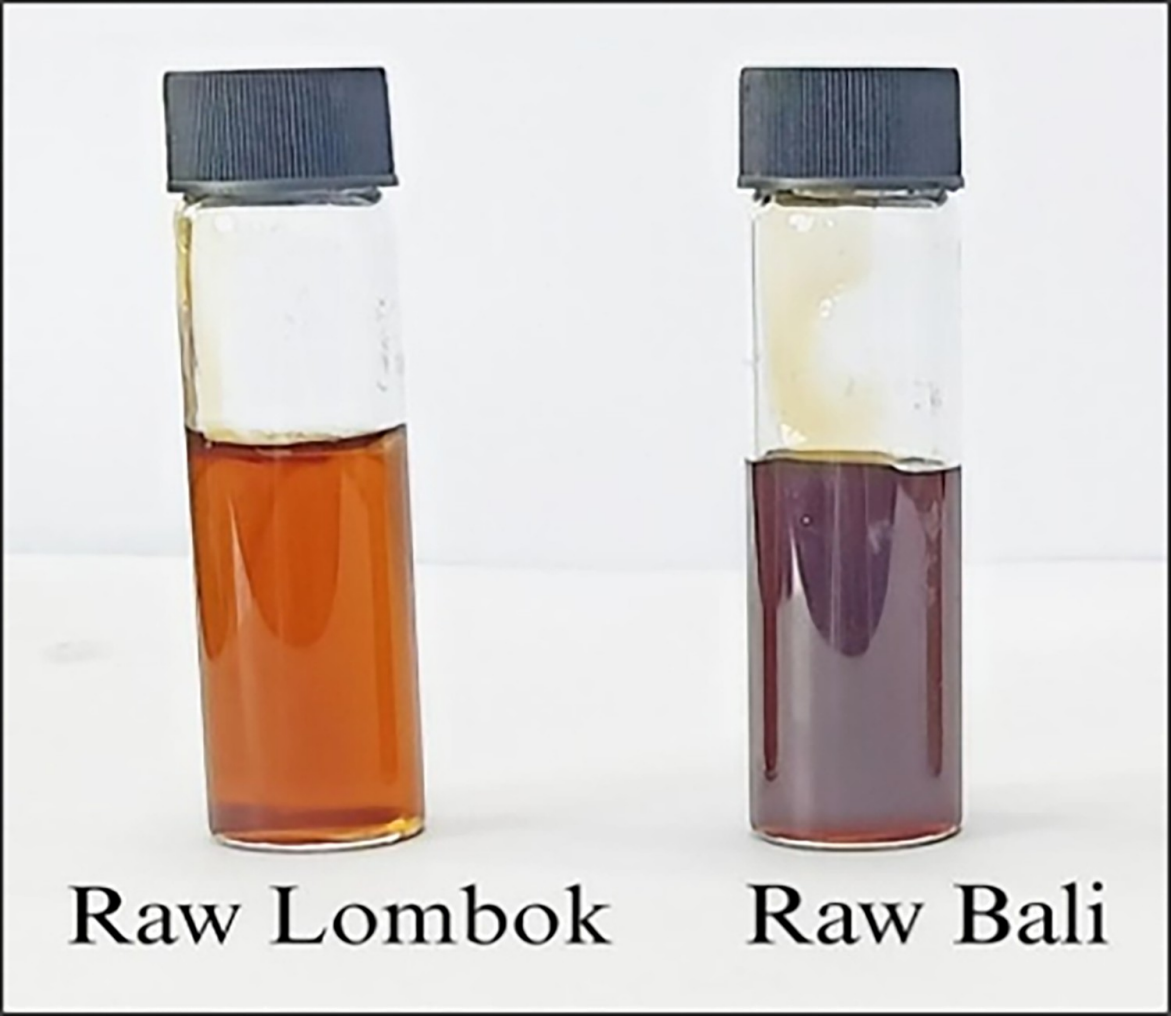
Raw Lombok (gold color) and raw Bali (slightly dark gold). Both were kept at room temperature (approximately 25°C) before analyses.

Meanwhile, raw honey samples were carefully selected from two local private suppliers: Honey Bee Farm in Desa Kuwum, Badung Regency, Bali (GPS coordinates: longitude 115.18728, latitude -8.45512) and Madu Lombok Utara in Desa Sukadana, North Lombok (GPS coordinates: longitude 116.39950, latitude -8.22134). The freshly collected honey samples were designated raw Lombok and raw Bali (Fig 1). To ensure statistical reliability in our sampling approach, three honey samples were collected from different hives within each locality/area in August 2023. This sample size allows for the application of basic statistical tests such as mean and standard deviation analysis to identify significant differences in honey composition in each area. Before analysis, all honey specimens were maintained within sealed screw-capped containers and stored under standard ambient conditions, specifically at ~25°C.

### Chemicals and reagents

Acquisition of Milli-Q ultrapure water and all chemicals was facilitated through reputable suppliers, namely Sigma-Aldrich (St. Louis, Missouri, United States) and Merck (Darmstadt, Germany). Ultrapure water, obtained via the ELGA Pure Lab Classic system (ELGA, Woodridge, IL, USA), was employed for all analyses. Chemicals and solvents, unless otherwise specified, were of analytical grade.

### Physicochemical properties

The physicochemical characteristics of raw Lombok and Bali honey were assessed in relation to local Tualang honey, used as the international benchmark. All measurements were conducted in triplicates for each attribute used in this study. The data are presented as mean values along with their corresponding standard deviations.

#### Ph

The pH determination of honey samples adhered to the AOAC method 962.19 (AOAC, 2016). Measurement of the pH value was conducted utilizing a water-resistant Mettler Toledo pH meter (Switzerland).

#### Free acidity

Free acidity was ascertained as described by the International Honey Commission (IHC) [16]. The titration procedure utilized NaOH (0.1 N) until pH 8.5. Following this, an additional 10 mL of NaOH was introduced to the sample, elevating the pH to approximately 10. Subsequent titration using HCl (0.1 N) gradually reduces the pH to 8.3. The volumes of NaOH and HCl consumed during each titration were recorded to calculate the total acidity of the sample. The acidity value was determined using:

Freeacidity=correctedvolumeofNaOHspent×10 (1)

#### Moisture content

The moisture content was measured using a digital refractometer (Digital ATAGO™ RX-5000α-Germany) set at 20°C. The refractive index (RI) values were transformed into moisture content (%) through the utilization of [18]:

Moisture(%)=[‐0.2681–log(R1‐1)/0.002243] (2)

#### Ash content

Ash was measured according to the standard set by AOAC Official 920.181 (AOAC,2016). Honey (100 g) was heated up to 600°C for two hours until a constant weight was obtained, using a Phoenix electrical furnace (CEM, United States). The resultant ash was quantified and expressed as ash content in grams per 100 g of honey.

#### Electroconductivity (EC)

Electrical conductivity (EC) testing was done on honey samples at a concentration 20% (w/v) honey. Honey (20 g) dissolved in 100 ml of ultrapure water. The HI-98311 electrical conductivity meter from Hanna Instruments, United States, was employed to measure electroconductivity.

#### Sugar content

Sugar content was measured using High-Performance Liquid Chromatography equipped with a Refractive Index Detector (HPLC-RID), as set by the AOAC Official 920.181 (AOAC,2016). A small aliquot of honey (5% w/v) was dissolved in a mixture of distilled water and acetonitrile at a 1:1 ratio, and the mixture was filter sterilized through a 0.22 μm nylon syringe filter. All chromatographic analyses were done on the Zorbax carbohydrate column (Agilent, California) (5 μm particle size x 250 mm length x 4.6 mm inner diameter) as the stationary phase, and eluted isocratically using acetonitrile: distilled water (75:25) as the mobile phase. A 40 μL aliquot of the honey sample was injected at a flow rate of 1.4 mL/min, and the column temperature was held at 30°C. The runtime per sample was 25 minutes, aligning with the glucose, fructose, maltose, and sucrose standards.

The sugar content was determined by correlating the retention times with established sugar standards. Quantitative analyses were executed by preparing standard fructose, glucose, sucrose, and maltose solutions across a range of concentrations (2–10 g/L). Calibration curves were then generated based on the corresponding peak areas of the analytes.

#### Hydromethylfurfural (HMF) content

The hydroxymethylfurfural (HMF) content in honey was measured based on AOAC Official Method 980.23 (AOAC, 2016) by using the HPLC 1200 Series (Agilent Technologies. U.S.A) equipped with an autosampler and a photodiode array detector. A 5% w/v of honey solution was prepared and filtered through a 0.45 μm nylon filter. The analytical column ZORBAX Eclipse XDB C18 column size 4.6 × 150 mm, 5 μm (Agilent Technologies, U.S.A) and mobile phase methanol:water (10:90; v/v) at flow rate of 0.5 mL/min. The injection volume was 20 μL, the column temperature was 25°C, and the absorbance read at 285 nm [19]. The HMF standard calibration curve was prepared for concentrations 5 to 25 mg/kg. The amount of HMF was recorded in the unit mg per kg of honey.

#### Total phenolic content (TPC)

The TPC was examined using the Folin Ciocalteu spectrophotometric method [20,21]. The honey sample (1 g) was diluted to 20 mL with distilled water. Subsequently, honey solution (1 mL) was pipetted into 5 mL of Folin Ciocalteu reagents (0.2 N) and incubated for 5 min at room temperature. Following this, 4 mL of 7.5% w/v aqueous sodium carbonate solution was added and further incubated at room temperature for 2 h. The absorbance of the resulting solution was measured at A_765nm_ against distilled water (blank) by using the UV-VIS spectrophotometer (Cary 60, Agilent Technologies, U.S.A). Gallic acid was employed at concentrations ranging from 20 to 100 ppm to generate the standard calibration curve. The total phenolic content was expressed in milligrams of gallic acid equivalent (GAE) per kilogram of honey.

#### Total flavonoid content (TFC)

The assessment of total flavonoid content was carried out following the procedure by Alothman et al., [22] utilizing quercetin as the standard (Sigma-Aldrich™) with a purity of 95%. To 1 mL of diluted honey (0.2 g/mL), 4 mL of distilled water was added, followed by the addition of 0.3 mL of a 5% w/v sodium nitrite solution. After a 5-minute interval, 0.3 mL of a 10% w/v aluminum chloride solution was introduced. Subsequently, 2 mL of 1 M NaOH was added after 6 minutes, and the total volume was adjusted to 10 mL by incorporating 2.4 mL of distilled water. The resulting solution was thoroughly mixed, and its absorbance was measured at A_510 nm_. A calibration curve was established using standard solutions of quercetin (0–100 μg/mL, Y = 0.0004x + 0.0007, R^2^ = 0.99). Each measurement was triplicated, and the results were presented as the total flavonoid concentration expressed in milligrams of quercetin equivalents (mg QE) per 100 grams of honey.

#### Antioxidant DPPH activity

The assessment of 2,2-diphenyl-1-picrylhydrazyl (DPPH) radical scavenging activity followed the methodology described by Ferreira et al., [23] with some modifications. Pure honey samples at various concentrations (0.98–62.5 mg/mL), ascorbic acid (0.24–31.25 μg/mL), and DPPH (0.04 mg/mL) were prepared in methanol. In 96-well plates, 100 μL of each sample was blended with 100 μL of a methanolic solution containing DPPH radical. The resulting mixture was thoroughly homogenized and allowed to incubate in darkness for 30 minutes. Absorbance readings were taken at A_517nm_ against a blank, which comprised a honey sample without DPPH radical to correct for the color influence of honey. Ascorbic acid served as the control. The Radical Scavenging Activity (RSA) was computed using the following formula:

$$Radical\;Scavenging\;Activity\;(RSA\%)=[A_{DPPH}‐A_{sample}]/A_{DPPH}\times 100$$ (3)

Where, A_sample_ denotes the absorbance of the solution after the addition of the sample at a specific level and A_DPPH_ represents the absorbance of the DPPH solution.

#### LCMS-MS-analysis of targeted flavonoid and polyphenolic compounds

Each honey sample (5 g) was dissolved in acidified water (pH 2) with stirring (200 rpm, 3 minutes) to a concentration of 10% (w/v), with a final volume of 100 mL. Subsequently, 10 mL of the diluted honey sample was transferred into a conical flask to which 450 mL of chloroform (used as the dispersive liquid-liquid microextraction extractant) and 750 μL of dimethyl carbonate (Me_2_CO_2_) were introduced. The mixture was stirred for 1 minute (200 rpm), then centrifuged (20,000g, for 10 minutes), before the supernatant was decanted and the settled phase transferred into a new ultracentrifuge tube. The residue was reconstituted in 100 μL of MeOH/H_2_O (1:4, v/v) and filtered into a 50 mL volumetric flask through a 0.045 μm nylon filter to eliminate particulate matter before chromatographic analysis.

Screening procedures were conducted using the Agilent 6560 Ion Mobility Quadrupole Time-Of-Flight (Q-TOF) LC/MS instrument. Compound identification and validation were meticulously performed by cross-referencing retention time (RT), molecular mass (mass), molecular formula, mass-to-charge ratio (m/z), and scores obtained from authoritative online databases (https://pubchem.ncbi.nlm.nih.gov/). The differential targeted (Diff tgt) compound match reliability threshold for the compounds identified was set to < 5 parts per million (ppm) [24].

## Results and discussion

The moisture content of honey indicates its water content, and reducing this moisture content is desirable because it lowers the risk of fermentation and spoilage in honey. Excessive moisture could expedite fermentation and spoilage, thus rendering a shorter shelf-life [25]. Although heating honey reduces water content to prevent fermentation and spoilage, this step tends to compromise quality. The study found that raw Lombok and Bali honey samples have moisture contents above 20%, slightly higher than the reported Tualang honey (18.3%). The observed values from the two Indonesian islands fell within the acceptable range set by the national standard SNI 8664:2018, which specifies a moisture content not exceeding 22% [26]. It’s worth noting that the honey from both Bali and Lombok is freshly collected, relying entirely on seasonal variations and weather conditions.

Similarly, pH is another parameter that is monitored in honey as it represents the acidity or alkalinity of honey. The pH ranges in Table 1. for the raw Lombok and Bali honey samples were found to be acidic, with pH corresponding to 4.1 and 3.9, respectively. The pH values of all honey samples fall within the standard pH limit of 3.40–6.10 as set by Codex Alimentarius [7]. Most importantly, the data points to a suitable condition for long-term storage of the honey samples, particularly under tropical conditions. Likewise, free acidity indicates the amounts of free acids present in honey and is measured in milli-equivalents per kilogram (meq/kg). Higher values may suggest increased acidity, which can influence flavor and stability in long-term storage. This study found that Lombok honey has the highest free acidity (78.5±2.14 meq/kg), followed by Bali (76.0±1.14 meq/kg). Since adhering to the quality standards requires an acidity level not exceeding 50 meq/kg, this honey sample meets the required quality level [7]. Moreover, literature has shown that a relatively higher acidity can somewhat exert an interesting flavor to the honey and also impart a satisfactory degree of storage stability [27]. Hence, the Lombok and Bali honey stands out in this aspect.

**Table 1:** Physicochemical analyses of raw Lombok and Bali honey compared to the published data on raw tualang honey from Sabah [17].

Honey types	Moisture (g/100g)	pH	Free acidity (meq/kg)	Ash (g/100g)	Electric conductivity (EC) (mS/cm)	HMF (mg/kg)	Total phenolic content (mg GAE/100g)	Total flavonoid content (mg QE/100g)	Antioxidant (DPPH) (%)
Raw Lombok	21.2±0.11	4.1±0.01	78.5±2.14	0.21±0.02	1.11±0.02	14.4±0.11	40.86±1.02	48.92±1.12	53.4±0.03
Raw Bali	22.7±0.12	3.9±0.04	76.0±1.14	0.14±0.01	0.85±0.05	17.6±0.25	55.57±1.11	55.58±1.22	65.34±0.02
Raw Tualang (Sabah)	18.3±0.21	3.5±0.01	29.5±6.14	1.3±0.02	5.06±0.02	6.4±0.11	17.86±0.02	6.92±0.02	2.4±0.03

1. HMF: Hydroxymethylfurfural; DPPH: 2,2-diphenyl-1-picryl-hydrazyl-hydrate; GAE: Gallic acid equivalents; QE: Quercetin equivalents.

2. Data presented as means ± standard deviation of three replicates.

Meanwhile, information on the ash content in honey signifies the inorganic mineral composition and is expressed as a percentage of the total weight. The ash content of honey can be used as a criterion of the authenticity and origin of honey and influences its color and flavor. Honeys with the greatest ash contents are darker and stronger flavored as the mineral components influence flavor by their natural alkalinity, while minerals impart a higher nutritional value. The Codex Alimentarius Commission established the essential composition and quality factors for honey as mineral content (ash) of not more than 0.6%. Honeydew honey and blends of honeydew and floral honey are not more than 1.0%. Determining the ash content of honey is important for assessing its quality and authenticity. The raw Lombok honey demonstrated an ash content of 0.21±0.02 g/100, while Bali honey exhibited 0.14±0.01 g/100, both considerably lower than Tualang honey, which displayed an ash content of 1.3±0.02 g/100. This discrepancy indicates a significantly higher mineral content in Tualang honey compared to Lombok and Bali varieties.

Electric conductivity is a measure of honey’s ability to conduct an electric current, influenced by the presence of ions. It provides honey’s mineral content and floral origin. Tualang honey has the highest electric conductivity (5.06±0.02), indicating a potentially higher mineral content. Nonetheless, the Lombok and Bali honey samples exhibit lower but comparable conductivity values of 1.11±0.02 mS/cm and 0.85±0.05 mS/cm, respectively. It is pertinent to indicate here that the higher conductivity in the Tualang honey corresponds well to its higher ash content (1.3±0.02 g/100) compared to the two other Indonesian honey samples.

Consequently, the hydroxymethylfurfural (HMF), represented as mg/kg, is a compound formed during honey processing and storage, and an elevated level conveys two issues related to an aging/long-term stored or heated honey samples, which two processes are deemed to reduce the quality of the honey. The Tualang honey (6.4±0.11 mg/kg) has the lowest HMF levels, suggesting the absence or lack of heat-related processing or long-term storage-related changes. It was a different scenario for the Lombok and Bali that recorded HMF levels of 14.4±0.11 mg/kg and 17.6±0.25 mg/kg, respectively, which, when compared to the HMF levels deemed by the Alimentarius [7] of HMF of not be more than 80 mg/kg the levels seen here remain low. The unexpected presence of HMF in Lombok and Bali honey may be attributed to specific environmental factors that warrant further investigation, as this discrepancy might be attributed to other unknown factors, or it could be an innate attribute of these honey samples.

The total phenolic compounds is an attribute that measures the content and antioxidant properties in honey samples, expressed in mg of gallic acid equivalent (GAE) per 100 g of honey. It was discovered that the Lombok (40.86±1.02 mg GAE/100g) and Bali (55.57±1.11 mg GAE/100g) honey samples gave higher total phenolic contents compared to the Tualang honey (17.86±0.02 mg GAE/100g). Higher phenolic contents in these two Indonesian honey samples are representative of their higher quality, which is associated with increased antioxidant capacity. Another class of compounds with anti-oxidative properties measured in this study are the flavonoids, observed in mg of quercetin equivalent (QE) per 100 g. The Bali honey (55.58±1.22 mg QE/100g) exhibited the highest total flavonoid content, followed by the Lombok (48.92±1.12 mg QE/100g) and Tualang (6.92±0.02 mg QE/100g) honey. Therefore, the consistent intake of honey rich in antioxidants is thought to contribute to reducing oxidative stress within the body, primarily through the neutralization of free radicals. Oxidative stress has been associated with various chronic health conditions, including numerous types of cancer [6]. Honey sourced from Lombok and Bali exhibits superior quality when compared to other reported varieties in the world, positively impacting the overall well-being of individuals who regularly incorporate it into their diets.

For the scavenging activity, the Antioxidant (DPPH) assay assesses the ability of honey to scavenge free radicals, which denotes the extent of antioxidant activity of the honey samples. It is worth noting here that higher DPPH percentages suggest stronger antioxidant capacity, thus a stronger protective effect on the consumers against the damaging effects of reactive oxygen species as a result of unhealthy lifestyles or due to extended exposure to polluted environments [6]. The highest scavenging activity was found in the Bali honey (65.34±0.02%), followed by the Lombok honey (53.4±0.03%) and, finally, the Tualang honey (2.4±0.03%). The remarkable antioxidant properties of the Bali and Lombok honey samples seen here are essential quality indicators closely tied to their phenolic content—potent oxygen radical scavengers from nectar and pollen [28] native to these islands. These compounds have been investigated for their potential in cancer pharmaceuticals, particularly for bolstering the immune system of humans [29,30]. Both Lombok and Bali honey, sourced from rural areas, demonstrate notably elevated total phenolic content and DPPH-radical scavenging activity, aligning with findings by Nicewicz et al., [31], which reported total phenolic contents and DPPH-radical scavenging activities of 98% and 23%, respectively in rural areas.

Likewise, the distinctive chemical composition of Southeast Asian honey samples seen here can be directly correlated with their reported and unreported botanical origin and floral diversity [32–34]. The naturally higher biodiversity associated with medicinal plant species and factors in the different abiotic factors in these regions further accentuate the distinctive chemical composition of these honey samples. Benefiting from natural fertilizers and an array of wild flowering plants in rural landscapes, the Lombok and Bali honey samples display considerably higher polyphenol content.

Honey mainly contains sugar components, especially fructose and glucose, followed by sucrose and maltose [35,36]. Typical honey is rich in glucose and fructose, and the percentage of sucrose in honey should be lower, less than 5% as set by Codex Alimentarius [7]. Sugar is also responsible for the viscosity, hygroscopic, and granulation characteristics of honey. However, honey samples from different regions are always prone to adulteration by unscrupulous individuals, who directly add a certain amount of sucrose syrup into the honey, alongside other physical-changing impurities, such as chemical colors, to attract consumers. Regular monitoring of quality and authenticity is germane in the honey industry to safeguard consumer health and ensure natural product quality [9,37,38].

Table 2 summarizes the results of the sugar contents in three different honey samples tested in this study. The total sugar content in raw Bali honey is the highest (75.21±0.11 g/100g), followed by raw Lombok honey (70.10±0.11g/100g) and the previously reported Tualang, Gelam, Kelulut, and Manuka honey [39] have relatively lower total sugar content. The sucrose content in Raw Bali honey is the highest at 2.39±0.01g/100g. The highest contents of glucose and fructose were observed in the Raw Bali honey, corresponding to 32.53±0.12 g/100g and 38.72±0.12 g/100g. Conversely, the Tualang honey has a relatively balanced ratio of glucose (28.79±0.08 g/100g) and fructose (31.73±0.14 g/100g).

**Table 2:** Sugar content of honey samples from Lombok and Bali Islands in comparison to those reported in honey by Zae et al. [39]*.

Honey	Total Sugar (g/100g)	Sucrose (g/100g)	Glucose (g/100g)	Fructose (g/100g)	Maltose (g/100g)
Raw Lombok	70.10±0.11	0.97±0.02	30.14±0.13	37.81±0.12	0.88±0.01
Raw Bali	75.21±0.11	2.39±0.01	32.53±0.12	38.72±0.12	1.14±0.02
*Tualang	63.40±0.01	1.48±0.01	28.79±0.08	31.73±0.14	ND
*Gelam	62.74±0.06	1.13±0.03	29.17±0.03	30.96±0.03	1.48±0.01
*Manuka	64.19±2.69	1.32±0.01	27.81±1.20	34.14±1.47	0.90±0.05

Likewise, maltose was detected in Raw Lombok (0.88±0.01 g/100g) and Raw Bali (1.14±0.02 g/100g), but absent in the Tualang honey. For comparison purposes, this study also compared known reports of maltose contents in the Gelam (1.48±0.01 g/100g), and Manuka (0.90±0.05 g/100g). Overall, the glucose content was found to be lower than the fructose content. This observation further reinforces the authenticity and high quality of the examined honey from Bali and Lombok.

Since this study found that both raw Lombok and Bali honey exhibited higher total flavonoid and phenolic content (Table 1), this prompted further investigation into the specific types of flavonoid and polyphenolic components present in each honey. Tables 3 and 4. summarize the flavonoids/flavanols and polyphenolic/phenolic acid compounds in raw Lombok and Bali honey, analysed by LC-MS/MS-QTOF. The chromatogram analyses of the raw data of Lombok and Bali honey can be accessed in S1 and S2 Appendix.

**Table 3:** Flavonoids and flavanol compounds were identified in raw Lombok honey using LC-MS/MS-QTOF (S1 Appendix).

No	Compound	Formula	Mass	Mass (Tgt)	Diff (Tgt, ppm)	RT	Charge	m/z	Scores
01	Galangin	C_15_H_10_O_5_	270.0535	270.0528	2.34	5.733	(M+NH_4_)^+^	288.08	46.18
02	Isorhamnetin	C_16_H_12_O_7_	316.0575	316.583	-2.40	7.123	(M+H)^+^	317.06	83.41
03	Chrysin	C_15_H_10_O_4_	254.0570	254.0578	-3.52	10.311	(M+H)^+^	255.06	78.91
04	(+) Catechin	C_15_H_14_O_6_	290.0786	290.0790	-1.56	1.466	(M+NH_4_)^+^	308.11	95.97
05	Naringenin	C_15_H_12_O_5_	272.0685	272.685	0.07	6.354	(M+NH_4_)+	290.10	93.59
06	Pinocembrin	C_15_H_12_O_4_	256.0731	256.0736	-1.87	6.861	(M+NH_4_)^+^	274.10	59.38
07	Apigenin	C_15_H_10_O_5_	270.0535	270.0528	2.34	5.733	(M+NH_4_)^+^	308.11	46.18
08	Quercetin	C_15_H_10_O_7_	302.0440	302.0427	4.38	4.572	(M+Na)^+^	325.03	67.67

RT: Retention time in minutes.

M/Z: Mass-to-charge ratio observed in the mass spectrometer.

Mass (tgt): Mass targeted for compound identification.

Diff (tgt, ppm): Differential (targeted, parts per million).

Score: Compound relevance in database.

**Table 4:** Characterization of the Phenolic acid compound identified in raw Lombok via LC-MS/MS-QTOF targeted analysis (S1 Appendix).

No	Compound	Formula	Mass	Mass (tgt)	Diff (Tgt, ppm)	RT	Charge	m/z	Scores
01	Caffeic acid	C_9_H_8_O_4_	180.0425	180.423	1.24	1.106	(M+NH_4_)^+^	198.07	84.25
02	Vanillic acid	C_8_H_8_O_4_	168.0420	168.0423	-1.34	1.891	(M+NH_4_)^+^	186.07	76.73
03	Ellagic acid	C_14_H_6_O_8_	302.0774	302.0790	1.92	0.616	(M+H)^+^	303.01	71.61
04	Syringic acid	C_9_H_10_O_5_	198.0535	198.0528	3.27	4.736	(M+H)^+^	199.05	83.48
05	Ferulic acid	C_10_H_10_O_4_	194.0577	194.0579	0.92	2.610	(M+NH_4_)^+^	212.09	76.50
06	3,4-dihydroxybenzoic acid	C_7_H_6_O_4_	154.0264	154.0266	-1.35	0.975	(M+NH_4_)^+^	172.06	80.15
07	Trans-cinnamic acid	C_9_H_8_O_2_	148.0517	148.0524	-4.86	3.035	(M+NH_4_)^+^	166.08	96.51
08	Gallic acid	C_7_H_6_O_5_	170.0212	170.0215	-2.16	4.294	(M+Na)^+^	193.01	46.95

RT: Retention time in minutes.

M/Z: Mass-to-charge ratio observed in the mass spectrometer.

Mass (tgt): Mass targeted for compound identification.

Diff (tgt, ppm): Differential (targeted, parts per million).

Score: Compound relevance in database.

Many studies, including food analytical research, have attempted to identify the flavonoid and phenolic profile of honey samples [29,40]. The presence of flavonoids and phenolic acids in honey is recognized for its antioxidant, anti-inflammatory, and potentially antimicrobial properties [41,42]. However, phenolic compounds are likely to enhance the complexity of honey’s flavor. For example, Galangin may impart a floral and herbaceous aroma, while caffeic acid imparts a bitter taste to honey [28]. This research indicates that these compounds may also play a role in the potential health advantages linked to consuming raw honey, as observed in samples from both Lombok and Bali.

Our analysis detected the presence of 16 distinct flavonoids/flavanols and phenolic acid compounds in raw Lombok honey samples, as in Tables 3 and 4. Among these, eight distinct flavonoids/flavanols were identified: Galangin, Isorhamnetin, Chrysin, (+)Catechin, Naringenin, Pinocembrin, Apigenin, and Quercetin. Additionally, eight phenolic acid compounds were observed in the same honey samples, namely Caffeic acid, Vanillic acid, Ellagic acid, Syringic acid, Ferulic acid, 3,4-dihydroxybenzoic acid, Trans-cinnamic acid, and Gallic acid. In the analysis of raw Bali honey samples, we discerned 10 distinct flavonoids and phenolic acid compounds, as detailed in Tables 5 and 6. Table 5 provides specifications for four distinct flavonoid compounds found in Bali honey: Kaempferol, Isorhamnetin, Rutin and Quercetin. Furthermore, Table 6 presents identification details for six different phenolic acid compounds, including Caffeic acid, Chlorogenic acid, Vanillic acid, Syringic acid, Gallic acid, and Sinapic acid.

**Table 5:** Flavonoids and flavanol compounds were identified in raw Bali honey using LC-MS/MS-QTOF (S2 Appendix).

No	Compound	Formula	Mass	Mass (Tgt)	Diff (Tgt, ppm)	RT	Charge	m/z	Scores
01	Kaempferol	C_15_H_10_O_6_	286.0474	286.0477	-1.18	5.806	(M+H)^+^	287.0547	80.82
02	Isorhamnetin	C_16_H_12_O_7_	316.0580	316.583	-0.86	5.708	(M+H)^+^	317.0653	99.53
03	Rutin	C_27_H_30_O_16_	610.1520	610.1534	-2.22	5.724	(M+H)^+^	611.1603	95.88
04	Quercetin	C_15_H_10_O_7_	302.0424	302.0427	-0.94	5.593	(M+H)^+^	303.0498	85.20

RT: Retention time in minutes.

m/z: Mass-to-charge ratio observed in the mass spectrometer.

Mass (tgt): Mass targeted for compound identification.

Diff (tgt, ppm): Differential (targeted, parts per million).

Score: Compound relevance in database.

**Table 6:** Characterization of the Phenolic acid compound identified in raw Bali via LC-MS/MS-QTOF targeted analysis (S2 Appendix).

No	Compound	Formula	Mass	Mass (Tgt)	Diff (Tgt, ppm)	RT	Charge	m/z	Scores
01	Caffeic acid	C_9_H_8_O_4_	180.04	180.4	0.95	4.798	(M+H)^+^	181.0497	85.06
02	Chlorogenic acid	C_16_H_18_O_9_	354.09	354.5	2.12	1.358	(M+H)^+^	355.1051	53.02
03	Vanillic acid	C_8_H_8_O_4_	168.04	168.6	-0.44	1.277	(M+NH_4_)^+^	186.0760	69.95
04	Syringic acid	C_9_H_10_O_5_	198.05	198.0	-3.10	5.806	(M+H)+	199.0588	73.45
05	Gallic acid	C_7_H_6_O_5_	170.0211	170.0215	-2.74	1.849	(M+Na)^+^	193.0103	89.65
06	Sinapic acid	C_11_H_12_O_5_	224.0675	224.0685	-4.29	5.152	(M+H)^+^	225.0750	76.45

RT: Retention time in minutes.

m/z: Mass-to-charge ratio observed in the mass spectrometer.

Mass (tgt): Mass targeted for compound identification.

Diff (tgt, ppm): Differential (targeted, parts per million).Score: Compound relevance in database.

Through a comparative analysis of the identified compounds, it becomes apparent that each honey sample (Lombok and Bali honey) exhibits specific flavonoids and phenolic acid compounds unique to its origin. However, certain compounds are shared or overlap between both honey samples, including Isorhamnetin, Quercetin, and Caffeic acid among flavonoids, and Caffeic acid, Vanillic acid, Syringic acid, and Gallic acid among phenolic compounds. Notably, the flavonoid compound Quercetin, identified in both honey samples, is renowned for its potent antioxidant properties, as documented in studies by Panche et al., [43]. Other flavonoids with high antioxidant properties include Kaempferol [44] and Rutin [45], the latter of which was only identified in the Bali honey sample.

The floral source and geographical location can significantly influence honey samples’ composition. For example, in another geographical region, galangin and chrysin were identified as the main flavonoids, whereas in the current study, galangin was exclusively found in Lombok honey but not in Bali honey [46]. Similarly, in a separate study, myricetin was the sole flavonoid detected in Eucalyptus honey from Spain, serving as a distinctive botanical marker for this type of honey [47]. However, our study did not detect myricetin in honey from Lombok and Bali. Therefore, myricetin can be considered a significant botanical marker for Eucalyptus honey. Discriminating honey based on floral sources presents challenges due to various factors impacting its chemical composition, including geographic origin, collection season, storage conditions, bee species, and interactions between chemical compounds and enzymes [48]. The presence of honey polyphenols seen here is generally attributed to the plant’s nectar. In particular, the quality and quantity of polyphenols can be influenced by geographical region, floral source, climatic conditions, and bee type [17]. Studies suggest that the polyphenol profile of honey, such as *p*-coumaric, gallic acid, caffeic acid, and ferulic acid, can serve as a floral marker to verify its botanical origin [5,49,50].

The identification of flavonoid and phenolic compounds is a common approach, but it might be insufficient, warranting the need for quantitative analysis to provide a more comprehensive understanding of the composition and potential health implications of consuming honey. Pertinently, our research successfully established an extensive flavonoid and phenolic profile for both Lombok and Bali honey samples. Current data offer valuable insights into the identification and potential presence of these compounds. However, quantitative information regarding their amounts in the honey samples is lacking, thus requiring further quantitative analysis to contribute to the body of knowledge pertaining to their composition and potential health benefits. Our findings highlight the unique characteristics of Lombok and Bali raw honey samples, which also point to their positive impact in guaranteeing the general well-being of regular consumers.

## Conclusions

The comprehensive physicochemical analysis in this study uncovers distinctive attributes in honeys sourced from Lombok and Bali. The honey samples exhibit favorable characteristics, such as the average moisture content typical of most Southeast Asian varieties, with a higher acidity level seen in Lombok honey with superior antioxidant properties than Malaysian honey. Despite Tualang honey displaying a higher ash content, its overall antioxidant capacity and phenolic and flavonoid content remain high. The Lombok- and Bali honey qualities exhibit promising nutritional and antioxidant properties and exceptional physicochemical properties unique to the region’s honey. The sugar contents in the raw Bali honey have a characteristically higher sucrose but still within the stipulated honey standard. In contrast, Tualang honey stands out with a balanced glucose and fructose ratio, and the absence of maltose adds to its distinct characteristics. However, it is worth noting that our study limitations include the absence of quantitative analysis for bioactive compounds and botanical source data concerning the identified chemical compositions. Metagenomic investigations may prove useful for future work in providing important determinants to authenticate these honeys. By investigating the microbial communities, metagenomic analyses can provide valuable insights into the origin and quality of honey, ensuring purity and unadulterated nature. This additional investigation can augment information that can be used to authenticate honey samples, especially from Lombok and Bali.

## Supporting information

S1 Appendix (PDF)

S2 Appendix (PDF)

## References

[1] Rysha A., et al., Evaluating the physicochemical properties of some kosovo’s and imported honey samples, Applied Sciences. 12 (2022) 629, doi: 10.3390/app12020629

[2] Palma-Morales M., Huertas J. R., and Rodríguez-Pérez C., A comprehensive review of the effect of honey on human health, Nutrients. 15 (2023) 3056, doi: 10.3390/nu15133056 37447382 PMC10346535

[3] Schouten C., Lloyd D., and Lloyd H., Beekeeping with the Asian honey bee (Apis cerana javana Fabr) in the Indonesian islands of Java, Bali, Nusa Penida, and Sumbawa, Bee world. 96 (2019) 45–49, doi: 10.1080/0005772X.2018.1564497

[4] Raffiudin R., et al., New haplotypes of Apis cerana in Indonesia: identification from mitochondrial and major royal jelly protein 2 genes, International Journal of Tropical Insect Science. 42 (2022) 389–401, doi: 10.1007/S42690-021-00556-X

[5] Samarghandian S., Farkhondeh T., and Samini F., Honey and health: A review of recent clinical research, Pharmacognosy research. 9 (2017) 121, doi: 10.4103/0974-8490.204647 28539734 PMC5424551

[6] Ranneh Y., et al., Honey and its nutritional and anti-inflammatory value, BMC complementary medicine and therapies. 21 (2021) 1–17, doi: 10.1186/s12906-020-03170-5 33441127 PMC7807510

[7] Alimentarius C., Revised codex standard for honey, Codex stan. 12 (2001) 1982,

[8] Strayer S. E., Everstine K., and Kennedy S., Economically motivated adulteration of honey: quality control vulnerabilities in the International honey market, Food Protection Trends. 34 (2014) 8–14.

[9] Se K. W., et al., A simple approach for rapid detection and quantification of adulterants in stingless bees (Heterotrigona itama) honey, Food Research International. 105 (2018) 453–460, doi: 10.1016/j.foodres.2017.11.012 29433236

[10] Norul L., et al., Physicochemical and radical scavenging activities of honey samples from Malaysia Agricultura’Sciences, Scirp. 4 (2013) 46–51, doi: 10.4236/as.2013.45B009

[11] Soares S., et al., Portuguese honeys from different geographical and botanical origins: A 4-year stability study regarding quality parameters and antioxidant activity, Molecules. 22 (2017) 1338, Portuguese honeys from different geographical and botanical origins: A 4-year stability study regarding quality parameters and antioxidant activity. doi: 10.3390/molecules22081338 28800099 PMC6152107

[12] Bogdanov S., Jurendic T., Sieber R., and Gallmann P., Honey for nutrition and health: a review, Journal of the American college of Nutrition. 27 (2008) 677–689, doi: 10.1080/07315724.2008.10719745 19155427

[13] de Almeida‐Muradian L. B., et al., Comparative study of the physicochemical and palynological characteristics of honey from M elipona subnitida and A pis mellifera, International Journal of Food Science & Technology. 48 (2013) 1698–1706, doi: 10.1101/2022.12.23.521720

[14] Ismail N., Characterisation of Malaysian honeys and electrochemical detection of gallotannin for pure honey identification, Universiti Teknologi Malaysia Johor Bahru, Malaysia. 2017 http://eprints.utm.my/id/eprint/79441/.

[15] Samat S., et al., Adulterated honey consumption can induce obesity, increase blood glucose level and demonstrate toxicity effects, Sains Malaysiana. 47 (2018) 353–365.

[16] Weihrauch M. R. and Diehl V., Artificial sweeteners—do they bear a carcinogenic risk?, Annals of Oncology. 15 (2004) 1460–1465, doi: 10.1093/annonc/mdh256 15367404

[17] Rajindran N., et al., Physicochemical Properties of a New Green Honey from Banggi Island, Sabah, Molecules. 27 (2022) 4164, doi: 10.3390/molecules27134164 35807409 PMC9268174

[18] Sesta G. and Lusco L., Refractometric determination of water content in royal jelly, Apidologie. 39 (2008) 225–232, doi: 10.1051/apido:2007053.

[19] Mendes E., Proença E. B., Ferreira I., and Ferreira M., Quality evaluation of Portuguese honey, Carbohydrate polymers. 37 (1998) 219–223, doi: 10.1016/S0144-8617(98)00063-0

[20] Singleton V. L., Orthofer R., and Lamuela-Raventós R. M., Analysis of total phenols and other oxidation substrates and antioxidants by means of folin-ciocalteu reagent, in *Methods in enzymology*. 1999, Elsevier. p. 152–178. doi: 10.1016/S0076-6879(99)99017-1

[21] Giusti M. M. and Wrolstad R. E., Current protocols in food analytical chemistry, Unit F. 1 (2001) 2.1–13, doi: 10.1002/0471142913.faf0402s01

[22] Alothman M., Bhat R., and Karim A., Antioxidant capacity and phenolic content of selected tropical fruits from Malaysia, extracted with different solvents, Food chemistry. 115 (2009) 785–788, doi: 10.1016/j.foodchem.2008.12.005

[23] Ferreira I. C., Aires E., Barreira J. C., and Estevinho L. M., Antioxidant activity of Portuguese honey samples: Different contributions of the entire honey and phenolic extract, Food chemistry. 114 (2009) 1438–1443, doi: 10.1016/j.foodchem.2008.11.028

[24] Ali A., et al., Lc-ms/ms-qtof screening and identification of phenolic compounds from australian grown herbs and their antioxidant potential, Antioxidants. 10 (2021) 1770, doi: 10.3390/antiox10111770 34829641 PMC8615083

[25] Singh I. and Singh S., Honey moisture reduction and its quality, Journal of food science and technology. 55 (2018) 3861–3871, doi: 10.1007/s13197-018-3341-5 30228384 PMC6133847

[26] Amri F. and Madelan S., Effort in Corrective Action and Improvement of Honey Product Quality Conforming to SNI 8664: 2018 in the Micro And Small Enterprise (Case Study: CV XYZ), doi: 10.5281/zenodo.7767653

[27] Chou W.-M., Liao H.-C., Yang Y.-C., and Peng C.-C., Evaluation of honey quality with stored time and temperatures, J. Food Nutr. Res. 8 (2020) 591–599, doi: 10.12691/jfnr-8-10-8

[28] Becerril-Sánchez A. L., Quintero-Salazar B., Dublán-García O., and Escalona-Buendía H. B., Phenolic compounds in honey and their relationship with antioxidant activity, botanical origin, and color, Antioxidants. 10 (2021) 1700, doi: 10.3390/antiox10111700 34829570 PMC8614671

[29] Lachman J., Orsák M., Hejtmánková A., and Kovářová E., Evaluation of antioxidant activity and total phenolics of selected Czech honeys, LWT-Food Science and Technology. 43 (2010) 52–58, doi: 10.1016/j.lwt.2009.06.008

[30] Kavanagh S., et al., Physicochemical properties and phenolic content of honey from different floral origins and from rural versus urban landscapes, Food chemistry. 272 (2019) 66–75, doi: 10.1016/j.foodchem.2018.08.035 30309595

[31] Nicewicz A. W., Nicewicz Ł., and Pawłowska P., Antioxidant capacity of honey from the urban apiary: A comparison with honey from the rural apiary, Scientific Reports. 11 (2021) 9695, doi: 10.1038/s41598-021-89178-4 33958670 PMC8102481

[32] Margońska H. B., Champion J., and Lipińska M. M., Preliminary Checklist of Malaxidinae and Liparidinae Representatives (Orchidaceae, Malaxideae) from Bali and Lombok Islands (Indonesia) with New Records, Diversity. 14 (2022) 398, doi: /10.3390/d14050398

[33] Rahayu S. M. and Andini A. S., Ethnobotanical study on medicinal plants in sesaot forest, narmada, West Lombok, Indonesia, Biosaintifika: Journal of Biology & Biology Education. 11 (2019) 234–242, doi: 10.15294/biosaintifika.v11i2.19314

[34] Andila P. S., Tirta I. G., Warseno T., and Sutomo S., Medicinal Plants Diversity Used by Balinese in Buleleng Regency, Bali, Journal of Tropical Biodiversity and Biotechnology. 8 (2023) 73303, doi: 10.22146/jtbb.73303

[35] da Silva P. M., et al., Honey: Chemical composition, stability and authenticity, Food chemistry. 196 (2016) 309–323, doi: 10.1016/j.foodchem.2015.09.051 26593496

[36] Kamal M. A. and Klein P., Determination of sugars in honey by liquid chromatography, Saudi journal of biological sciences. 18 (2011) 17–21, doi: 10.1016/j.sjbs.2010.09.003 23961099 PMC3730891

[37] Fakhlaei R., et al., The toxic impact of honey adulteration: A review, Foods. 9 (2020) 1538, doi: 10.3390/foods9111538 33114468 PMC7692231

[38] Rachineni K., et al., Identifying type of sugar adulterants in honey: Combined application of NMR spectroscopy and supervised machine learning classification, Current research in food science. 5 (2022) 272–277, doi: 10.1016/j.crfs.2022.01.008 35141528 PMC8816647

[39] Zae T., et al., Comparison of selected local honey with Manuka honey based on their nutritional and antioxidant properties, Food Res. 4 (2020) 205–213, doi: 10.26656/fr.2017.4(S1).S12

[40] Bertoncelj J., Doberšek U., Jamnik M., and Golob T., Evaluation of the phenolic content, antioxidant activity and colour of Slovenian honey, Food chemistry. 105 (2007) 822–828, doi: 10.1016/j.foodchem.2007.01.060

[41] Young G.-W. Z. and Blundell R., A review on the phytochemical composition and health applications of honey, Heliyon. (2023), doi: 10.1016/j.heliyon.2022.e12507 36755588 PMC9900486

[42] Cianciosi D., et al., Phenolic compounds in honey and their associated health benefits: A review, Molecules. 23 (2018) 2322, doi: 10.3390/molecules23092322 30208664 PMC6225430

[43] Panche A. N., Diwan A. D., and Chandra S. R., Flavonoids: an overview, Journal of nutritional science. 5 (2016) e47, doi: 10.1017/jns.2016.41 28620474 PMC5465813

[44] Wang J., et al., Antitumor, antioxidant and anti-inflammatory activities of kaempferol and its corresponding glycosides and the enzymatic preparation of kaempferol, PLoS One. 13 (2018) e0197563, doi: 10.1371/journal.pone.0197563 29771951 PMC5957424

[45] Enogieru A. B., et al., Rutin as a potent antioxidant: Implications for neurodegenerative disorders, Oxidative Medicine and Cellular Longevity. 2018 (2018) doi: 10.1155/2018/6241017 30050657 PMC6040293

[46] Ferreres F., Andrade P., and Tomás-Barberán F. A., Natural occurrence of abscisic acid in heather honey and floral nectar, Journal of Agricultural and Food Chemistry. 44 (1996) 2053–2056, doi: 10.1021/jf9507553

[47] Cheung Y., Meenu M., Yu X., and Xu B., Phenolic acids and flavonoids profiles of commercial honey from different floral sources and geographic sources, International Journal of Food Properties. 22 (2019) 290–308, doi: 10.1080/10942912.2019.1579835

[48] Kaškonienė V. and Venskutonis P. R., Floral markers in honey of various botanical and geographic origins: a review, Comprehensive reviews in food science and food safety. 9 (2010) 620–634, doi: 10.1111/j.1541-4337.2010.00130.x 33467823

[49] Saxena S., Gautam S., and Sharma A., Physical, biochemical and antioxidant properties of some Indian honeys, Food chemistry. 118 (2010) 391–397, doi: 10.1016/j.foodchem.2009.05.001

[50] Pyrzynska K. and Biesaga M., Analysis of phenolic acids and flavonoids in honey, TrAC trends in analytical chemistry. 28 (2009) 893–902, doi: 10.1016/j.trac.2009.03.015

