# Supplementary material for: Physicochemical and antioxidant properties of Apis cerana honey from Lombok and Bali Islands
Source: PLoS One. 2024 Apr 5;19(4):e0301213. doi: 10.1371/journal.pone.0301213 (PMC10997079; doi:10.1371/journal.pone.0301213)
Supplement: S1 Appendix — (PDF) [file pone.0301213.s001.pdf]

Target Screening Report

Agilent | Trusted Answers

Sample Information

**Name**	Sample R-B1-201123	**Data File Path**	D:\MassHunter\Data\Saeed Ullah\R-B1-201123.d
**Sample ID**	Lombok honey	**Acq. Time (Local)**	11/20/2023 11:48:13 AM (UTC+08:00)
**Instrument**	Instrument 1	**Method Path (Acq)**	D:\MassHunter\Methods\Training Method 210822.m
**MS Type**	QTOF	**Version (Acq SW)**	6200 series TOF/6500 series Q-TOF B.09.00 (B9044.1 SP1)
**Inj. Vol. (ul)**	1	**IRM Status**	All ions missed
**Position**	P1-A1	**Method Path (DA)**	D:\MassHunter\Methods\10.0\Default-LCMS.m
**Plate Pos.**		**Target Source Path**	
**Operator**		**Result Summary**	18 qualified (30 targets)

Sample Chromatograms

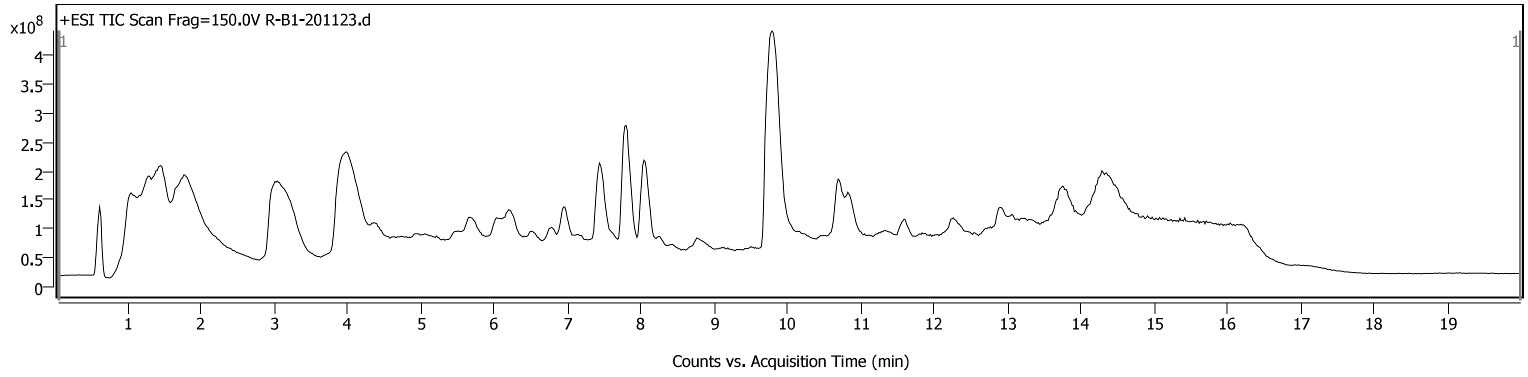

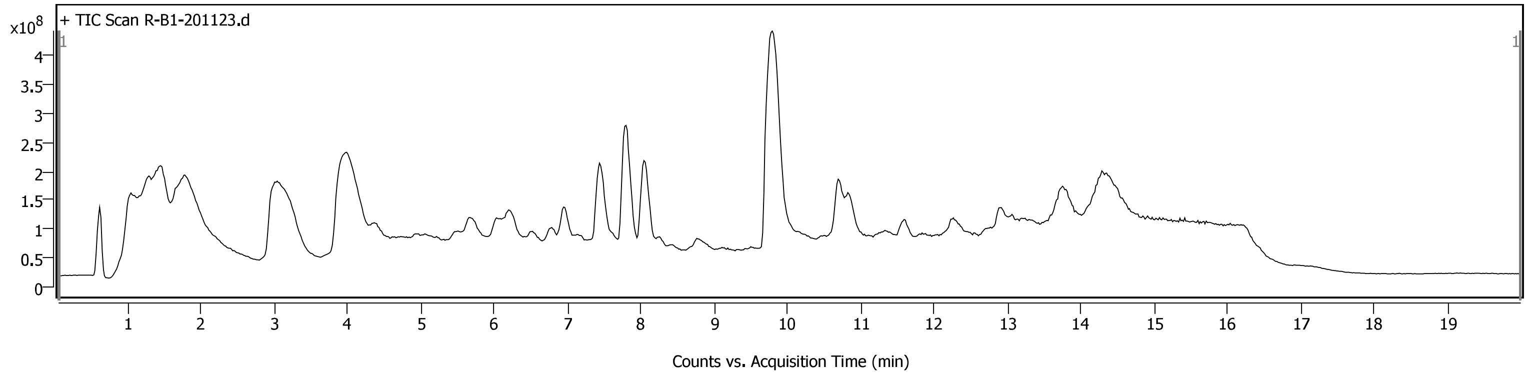

Compound Summary

Cpd	Name	Formula	CAS	RT	Mass	Mass (Tgt)	Diff (Tgt, ppm)	Score	Algorithm
1	Kaempferol	C15 H10 O6		1.220	286.0444	286.0477	-11.79	54.57	FBF
2		C15 H10 O6		1.220	286.0444	286.0477	-11.79	54.57	FBF
3	Galangin	C15 H10 O5		5.733	270.0535	270.0528	2.34	46.18	FBF
4	Isorhamnetin	C16 H12 O7		7.123	316.0575	316.0583	-2.40	83.41	FBF
5	chrysin	C15 H10 O4		10.311	254.0570	254.0579	-3.52	78.91	FBF
6		C9 H8 O2		3.035	148.0517	148.0524	-4.86	96.51	FBF
7	Gallic acid	C7 H6 O5		4.294	170.0212	170.0215	-2.16	46.95	FBF
8		C7 H7 N O2		1.188	137.0475	137.0477	-1.16	78.86	FBF
9	Chorogenic acid	C16 H18 O9		5.749	354.0915	354.0951	-10.05	21.47	FBF
10	(+)catechin	C15 H14 O6		1.466	290.0786	290.0790	-1.56	95.97	FBF
11	Naringenin	C15 H12 O5		6.354	272.0685	272.0685	0.07	93.59	FBF
12	Rutin	C27 H30 O16		3.084	610.1599	610.1534	10.71	18.87	FBF
13		C9 H10 O4		2.496	182.0599	182.0579	11.17	79.17	FBF
14	Sinapic acid	C11 H12 O5		4.605	224.0672	224.0685	-5.61	80.89	FBF
15		C16 H12 O7		7.123	316.0575	316.0583	-2.40	83.41	FBF
16		C15 H10 O4		10.311	254.0570	254.0579	-3.52	78.91	FBF
17	Pincocarbin	C15 H12 O4		6.861	256.0731	256.0736	-1.87	59.38	FBF
18	Caffeic acid	C9 H8 O4		1.106	180.0425	180.0423	1.24	78.57	FBF
19		C16 H18 O9		5.749	354.0915	354.0951	-10.05	21.47	FBF
20	Vanallic acid	C8 H8 O4		1.891	168.0420	168.0423	-1.34	83.02	FBF
21	Benzoic acid	C7 H5 O2		7.482	121.0328	121.0290	32.03	7.06	FBF
22	Elagic acid	C14 H6 O8		0.616	302.0068	302.0063	1.92	46.10	FBF
23	Syringic acid	C9 H10 O5		4.736	198.0535	198.0528	3.27	79.75	FBF
24	Ferulic acid	C10 H10 O4		2.610	194.0577	194.0579	-0.92	78.45	FBF
25	3,4-dihydroxybenzoic acid	C7 H6 O4		0.975	154.0264	154.0266	-1.35	83.63	FBF
26	Hesperdin	C16 H14 O6		6.632	302.0774	302.0790	-5.56	58.98	FBF
27	Apigenin	C15 H10 O5		5.733	270.0535	270.0528	2.34	46.18	FBF
28	epicatchin	C15 H14 O6		1.466	290.0786	290.0790	-1.56	95.97	FBF
29		C15 H10 O6		1.220	286.0444	286.0477	-11.79	54.57	FBF
30	Quercetin	C15 H10 O7		4.572	302.0440	302.0427	4.38	67.67	FBF

Compound Details

Target Screening Report

Cpd. 1: C15 H10 O6

Name	Formula	RT	RI	Mass	Diff (Tgt, ppm)	CAS	ID Source	Score	Algorithm
	C15 H10 O6	1.220		286.0444	-11.79		FBF	54.57	FBF

Species	m/z	Score (Tgt)	Score (Lib)	Score (DB)	Score (MFG)	Score (RT)
(M+H)+ (M+NH4)+ (M+Na)+	287.0519 304.0732 309.0409	54.57				

Compound Chromatograms (overlaid)

Structure

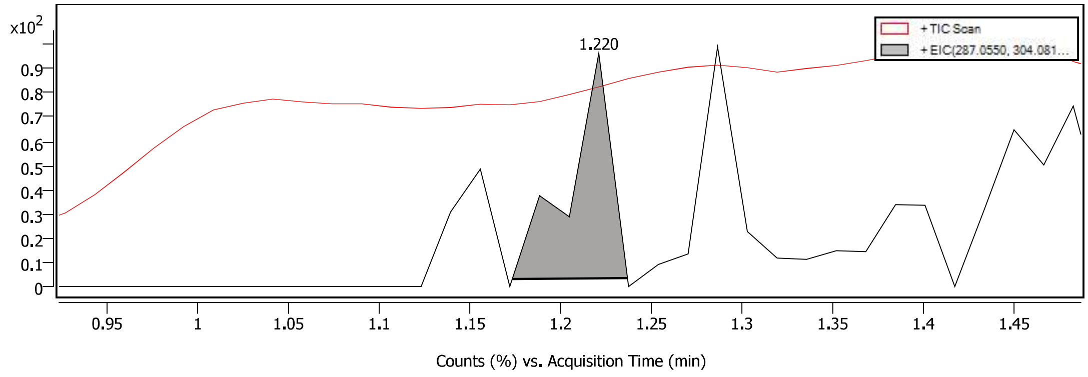

Compound Spectra (overlaid)

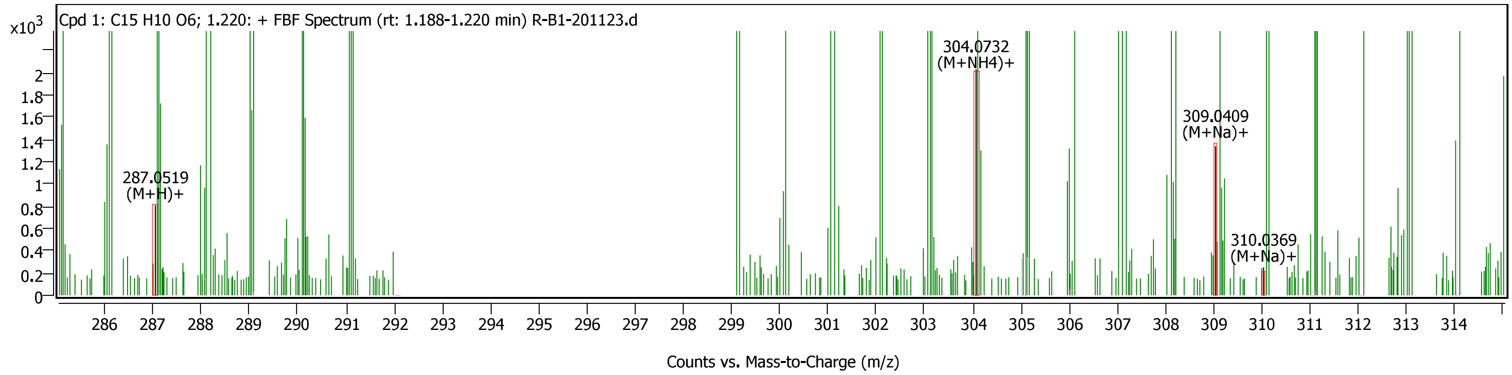

Compound ID Table

Name	Formula	Species	RT	RT Diff	Mass	CAS	ID Source	Score	Score (Lib)	Score (Tgt)
	C15 H10 O6	(M+H)+ (M+NH4)+ (M+Na)+	1.220		286.0444		FBF	54.57		54.57

Cpd. 2: C15 H10 O6

Name	Formula	RT	RI	Mass	Diff (Tgt, ppm)	CAS	ID Source	Score	Algorithm
	C15 H10 O6	1.220		286.0444	-11.79		FBF	54.57	FBF

Species	m/z	Score (Tgt)	Score (Lib)	Score (DB)	Score (MFG)	Score (RT)
(M+H)+ (M+NH4)+ (M+Na)+	287.0519 304.0732 309.0409	54.57				

Compound Chromatograms (overlaid)

Structure

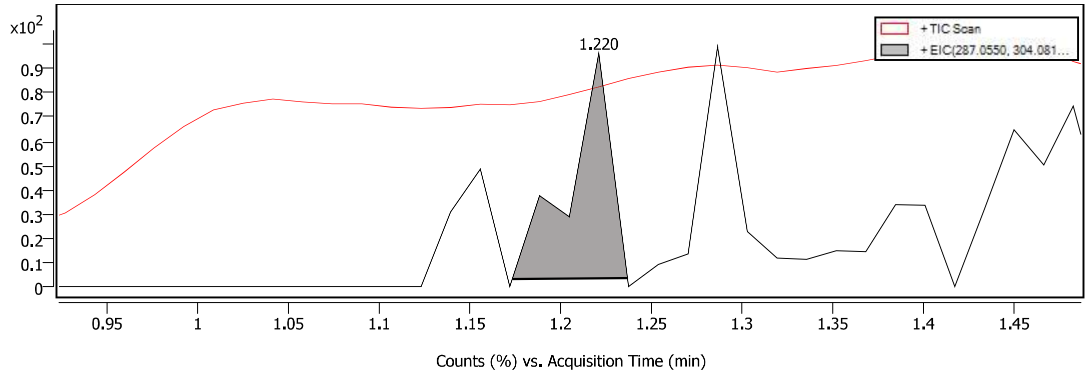

Compound Spectra (overlaid)

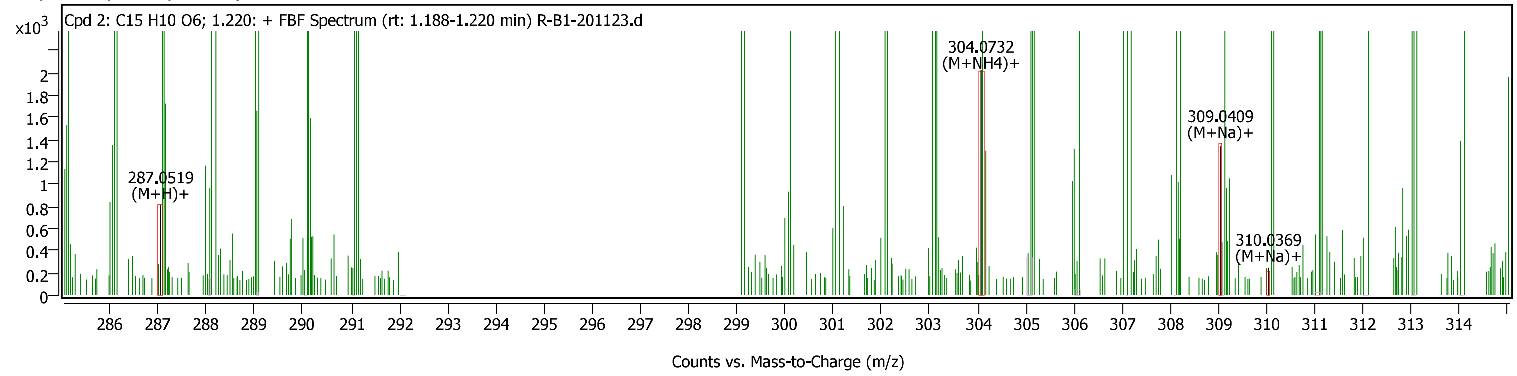

Compound ID Table

Name	Formula	Species	RT	RT Diff	Mass	CAS	ID Source	Score	Score (Lib)	Score (Tgt)
	C15 H10 O6	(M+H)+ (M+NH4)+ (M+Na)+	1.220		286.0444		FBF	54.57		54.57

Cpd. 3: C15 H10 O5

Name	Formula	RT	RI	Mass	Diff (Tgt, ppm)	CAS	ID Source	Score	Algorithm
	C15 H10 O5	5.733		270.0535	2.34		FBF	46.18	FBF

Species	m/z	Score (Tgt)	Score (Lib)	Score (DB)	Score (MFG)	Score (RT)
(M+NH4)+	288.0873	46.18				

Compound Chromatograms (overlaid)

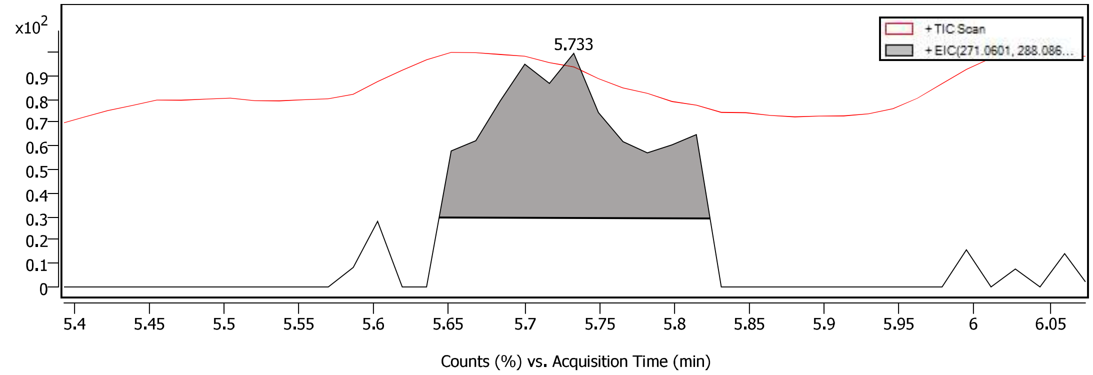

Structure

Compound Spectra (overlaid)

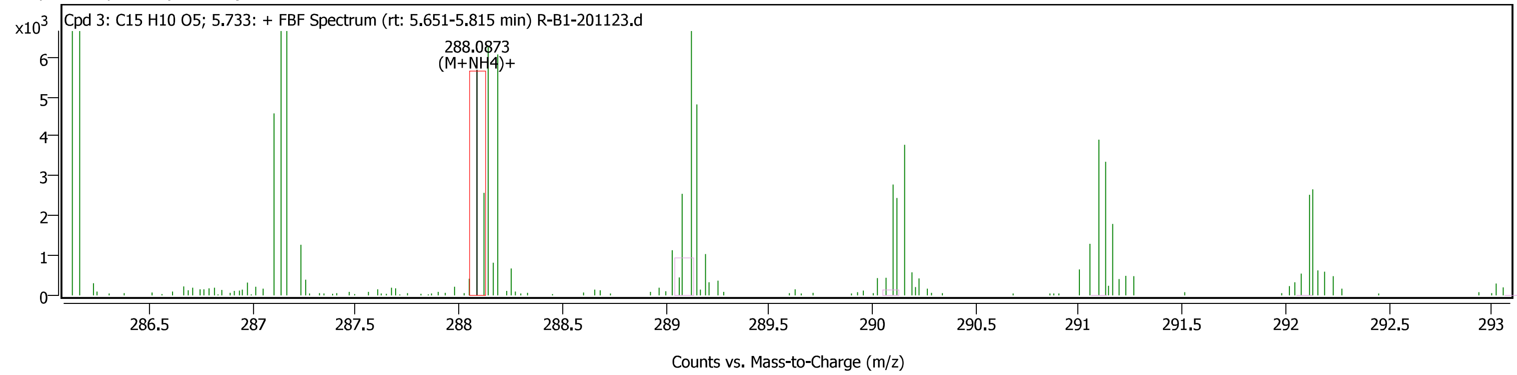

Compound ID Table

Name	Formula	Species	RT	RT Diff	Mass	CAS	ID Source	Score	Score (Lib)	Score (Tgt)
	C15 H10 O5	(M+NH4)+	5.733		270.0535		FBF	46.18		46.18

Cpd. 4: C16 H12 O7

Name	Formula	RT	RI	Mass	Diff (Tgt, ppm)	CAS	ID Source	Score	Algorithm
	C16 H12 O7	7.123		316.0575	-2.40		FBF	83.41	FBF

Species	m/z	Score (Tgt)	Score (Lib)	Score (DB)	Score (MFG)	Score (RT)
(M+H)+ (M+NH4)+ (M+Na)+	317.0651 334.0886 339.0438	83.41				

Compound Chromatograms (overlaid)

Structure

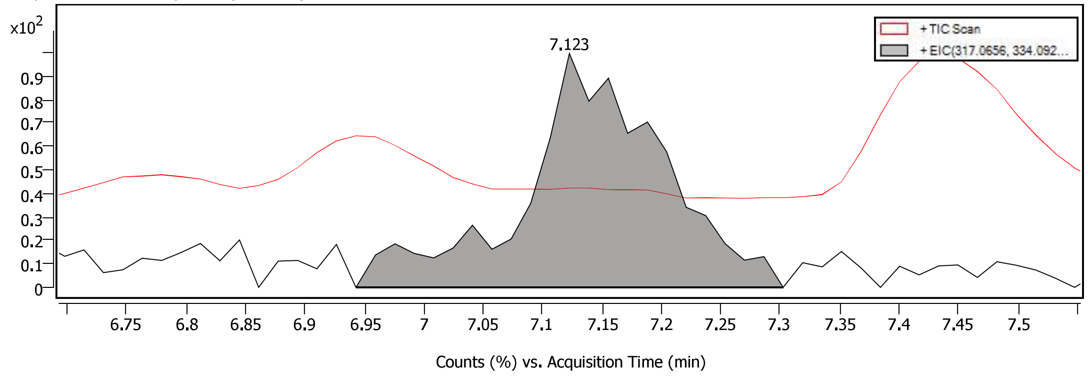

Compound Spectra (overlaid)

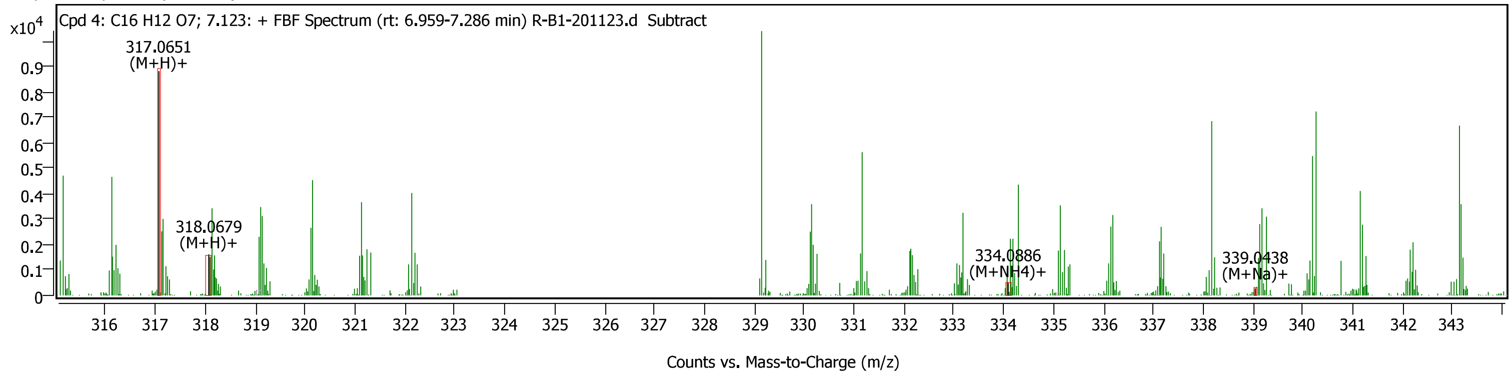

Compound ID Table

Name	Formula	Species	RT	RT Diff	Mass	CAS	ID Source	Score	Score (Lib)	Score (Tgt)
	C16 H12 O7	(M+H)+ (M+NH4)+ (M+Na)+	7.123		316.0575		FBF	83.41		83.41

Cpd. 5: C15 H10 O4

Name	Formula	RT	RI	Mass	Diff (Tgt, ppm)	CAS	ID Source	Score	Algorithm
	C15 H10 O4	10.311		254.0570	-3.52		FBF	78.91	FBF

Species	m/z	Score (Tgt)	Score (Lib)	Score (DB)	Score (MFG)	Score (RT)
(M+H)+ (M+NH4)+	255.0643 272.0909	78.91				

Compound Chromatograms (overlaid)

Structure

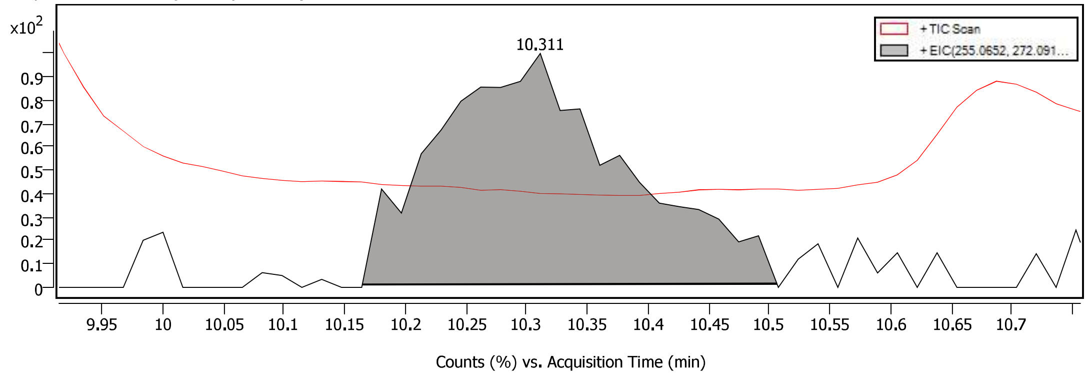

Compound Spectra (overlaid)

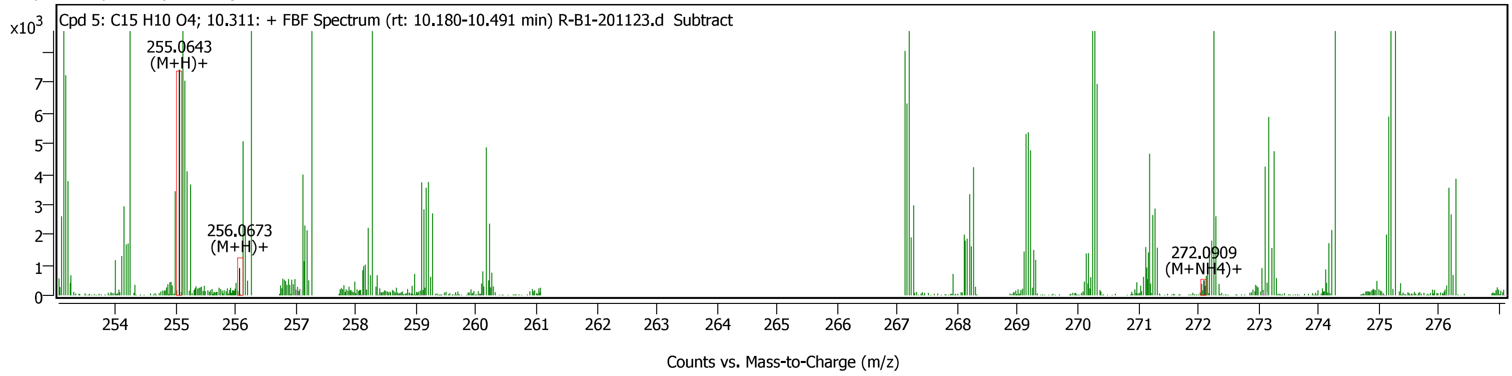

Compound ID Table

Name	Formula	Species	RT	RT Diff	Mass	CAS	ID Source	Score	Score (Lib)	Score (Tgt)
	C15 H10 O4	(M+H)+ (M+NH4)+	10.311		254.0570		FBF	78.91		78.91

Cpd. 6: C9 H8 O2

Name	Formula	RT	RI	Mass	Diff (Tgt, ppm)	CAS	ID Source	Score	Algorithm
	C9 H8 O2	3.035		148.0517	-4.86		FBF	96.51	FBF

Species	m/z	Score (Tgt)	Score (Lib)	Score (DB)	Score (MFG)	Score (RT)
(M+H)+ (M+NH4)+ (M+Na)+	149.0593 166.0855 171.0430	96.51				

Compound Chromatograms (overlaid)

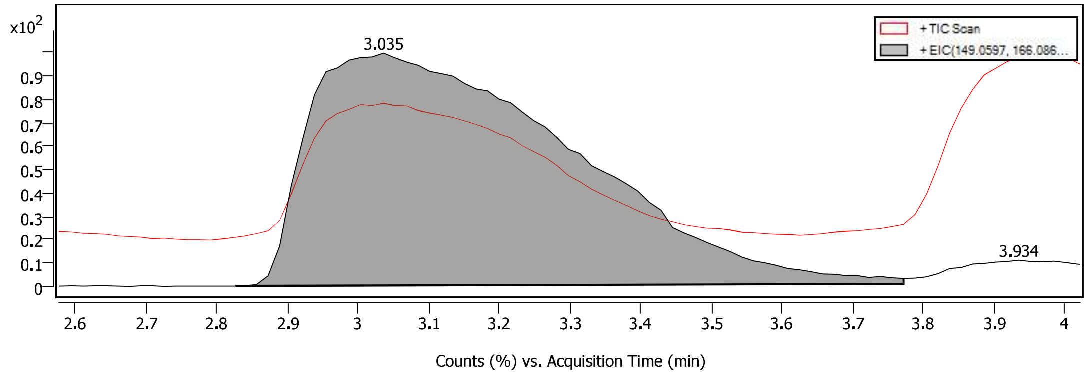

Structure

Compound Spectra (overlaid)

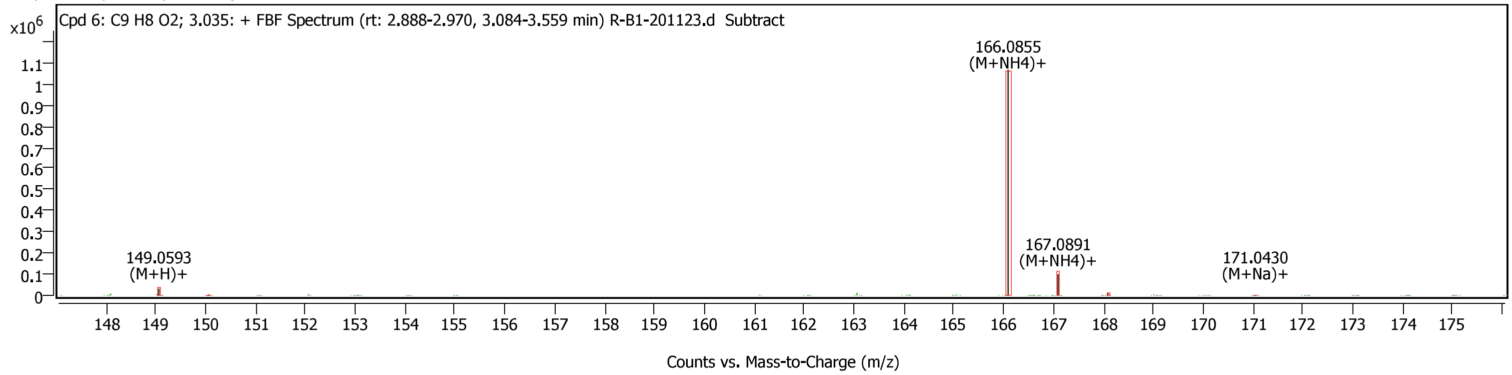

Compound ID Table

Name	Formula	Species	RT	RT Diff	Mass	CAS	ID Source	Score	Score (Lib)	Score (Tgt)
	C9 H8 O2	(M+H)+ (M+NH4)+ (M+Na)+	3.035		148.0517		FBF	96.51		96.51

Cpd. 7: C7 H6 O5

Name	Formula	RT	RI	Mass	Diff (Tgt, ppm)	CAS	ID Source	Score	Algorithm
	C7 H6 O5	4.294		170.0212	-2.16		FBF	46.95	FBF

Species	m/z	Score (Tgt)	Score (Lib)	Score (DB)	Score (MFG)	Score (RT)
(M+Na)+	193.0104	46.95				

Compound Chromatograms (overlaid)

Structure

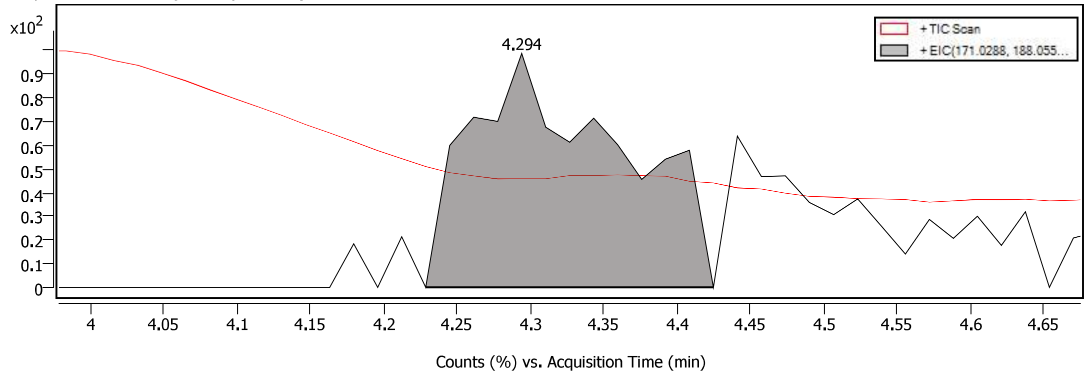

Compound Spectra (overlaid)

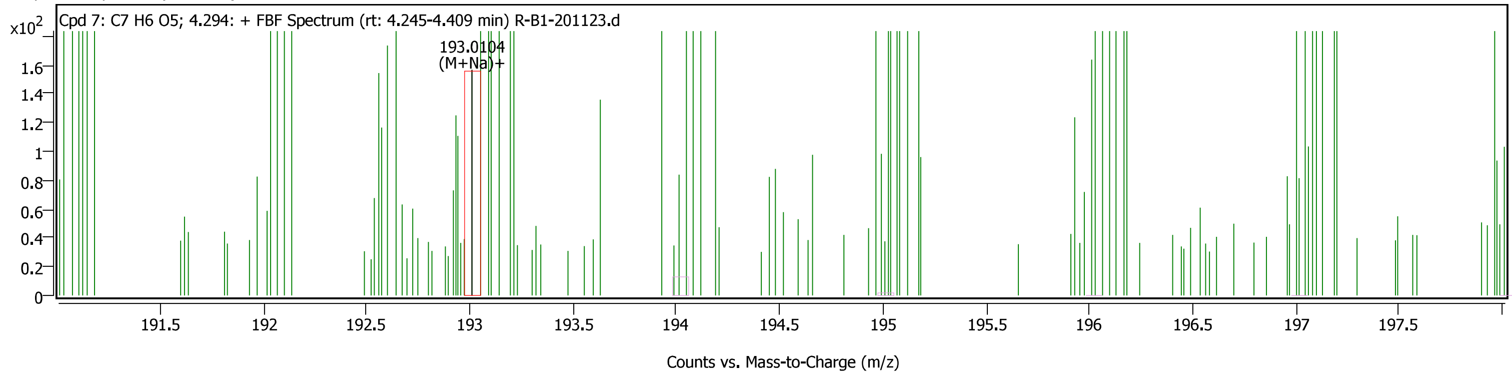

Compound ID Table

Name	Formula	Species	RT	RT Diff	Mass	CAS	ID Source	Score	Score (Lib)	Score (Tgt)
	C7 H6 O5	(M+Na)+	4.294		170.0212		FBF	46.95		46.95

Cpd. 8: C7 H7 N O2

Name	Formula	RT	RI	Mass	Diff (Tgt, ppm)	CAS	ID Source	Score	Algorithm
	C7 H7 N O2	1.188		137.0475	-1.16		FBF	78.86	FBF

Species	m/z	Score (Tgt)	Score (Lib)	Score (DB)	Score (MFG)	Score (RT)
(M+H)+ (M+NH4)+ (M+Na)+	138.0551 155.0805 160.0416	78.86				

Compound Chromatograms (overlaid)

Structure

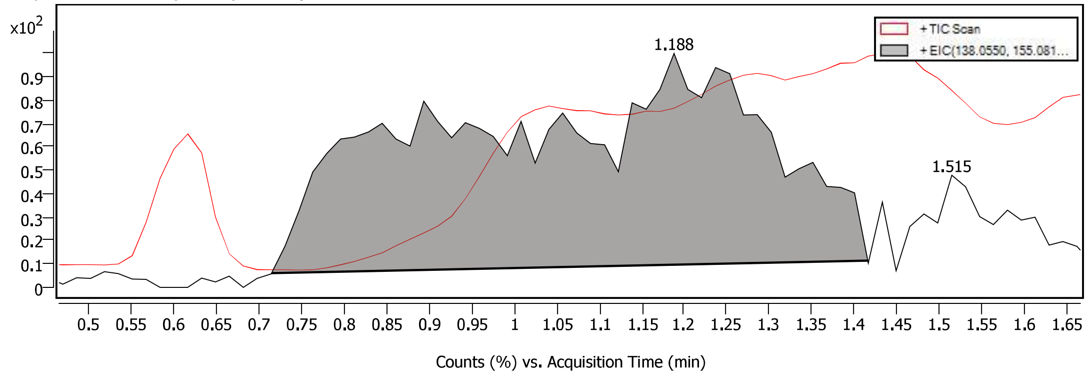

Compound Spectra (overlaid)

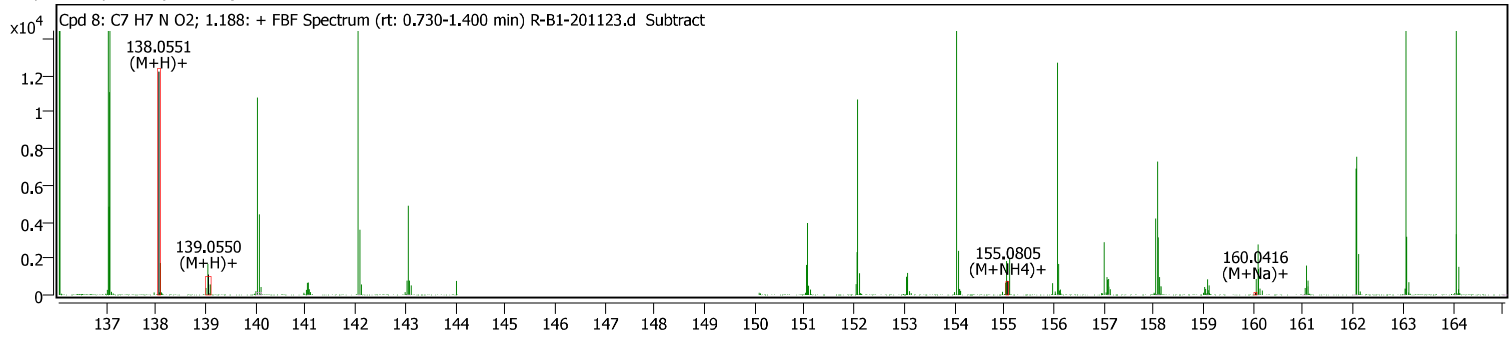

Compound ID Table

Name	Formula	Species	RT	RT Diff	Mass	CAS	ID Source	Score	Score (Lib)	Score (Tgt)
	C7 H7 N O2	(M+H)+ (M+NH4)+ (M+Na)+	1.188		137.0475		FBF	78.86		78.86

Cpd. 9: C16 H18 O9

Name	Formula	RT	RI	Mass	Diff (Tgt, ppm)	CAS	ID Source	Score	Algorithm
	C16 H18 O9	5.749		354.0915	-10.05		FBF	21.47	FBF

Species	m/z	Score (Tgt)	Score (Lib)	Score (DB)	Score (MFG)	Score (RT)
(M+H)+	355.0988	21.47				

Compound Chromatograms (overlaid)

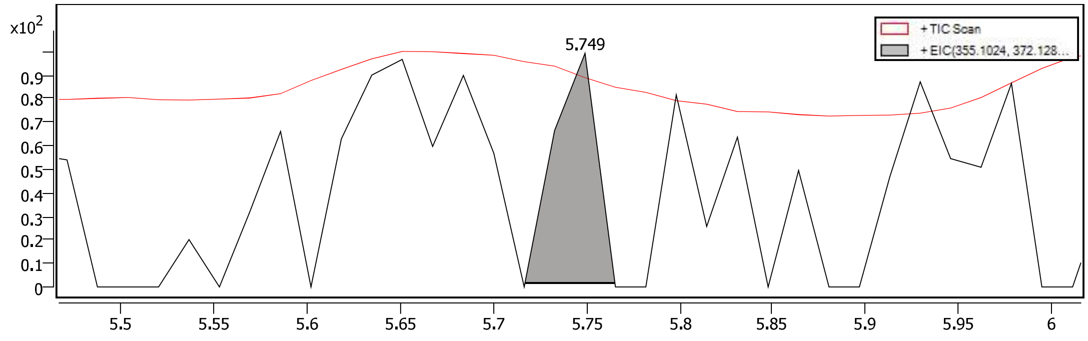

Structure

Compound Spectra (overlaid)

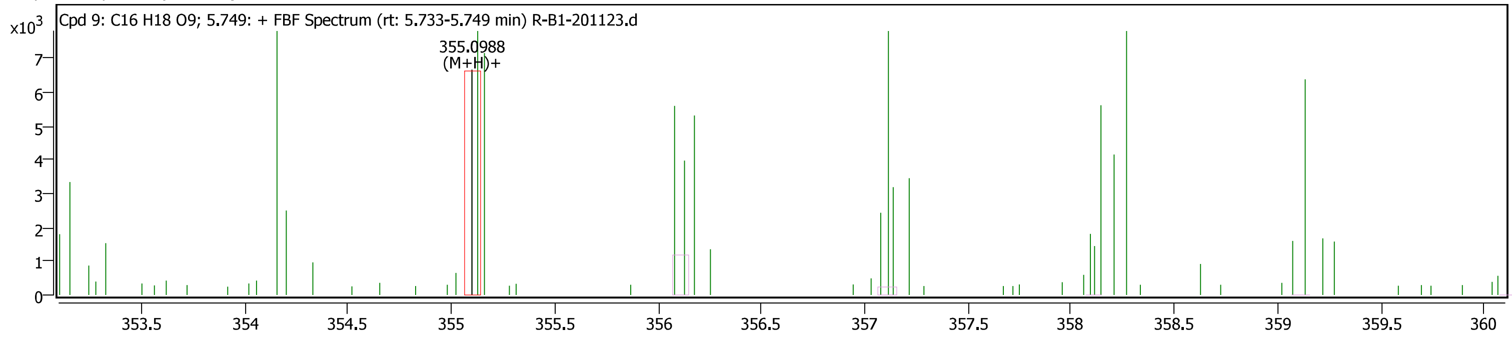

Compound ID Table

Name	Formula	Species	RT	RT Diff	Mass	CAS	ID Source	Score	Score (Lib)	Score (Tgt)
	C16 H18 O9	(M+H)+	5.749		354.0915		FBF	21.47		21.47

Cpd. 10: C15 H14 O6

Name	Formula	RT	RI	Mass	Diff (Tgt, ppm)	CAS	ID Source	Score	Algorithm
	C15 H14 O6	1.466		290.0786	-1.56		FBF	95.97	FBF

Species	m/z	Score (Tgt)	Score (Lib)	Score (DB)	Score (MFG)	Score (RT)
(M+H)+ (M+NH4)+	291.0903 308.1121	95.97				

Compound Chromatograms (overlaid)

Structure

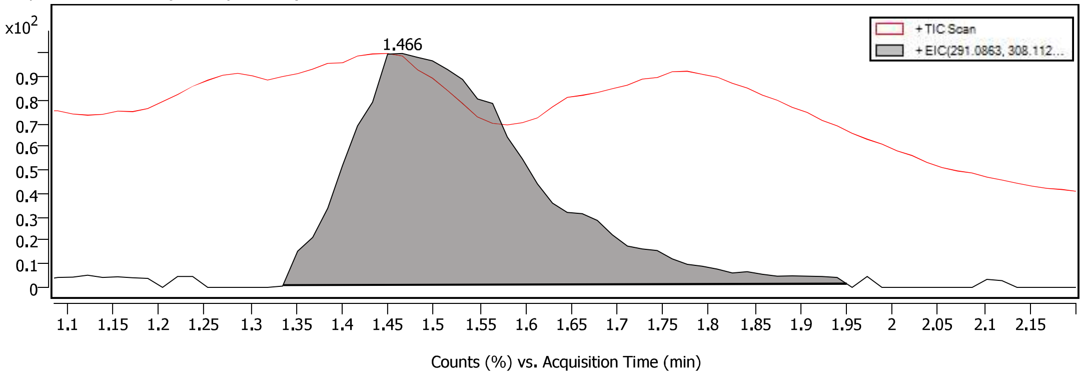

Compound Spectra (overlaid)

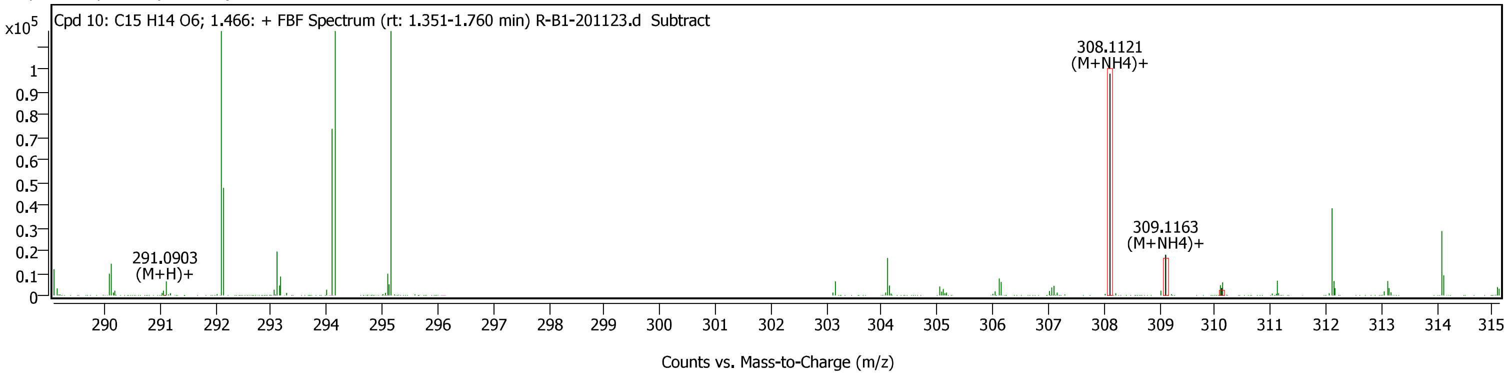

Compound ID Table

Name	Formula	Species	RT	RT Diff	Mass	CAS	ID Source	Score	Score (Lib)	Score (Tgt)
	C15 H14 O6	(M+H)+ (M+NH4)+	1.466		290.0786		FBF	95.97		95.97

Cpd. 11: C15 H12 O5

Name	Formula	RT	RI	Mass	Diff (Tgt, ppm)	CAS	ID Source	Score	Algorithm
	C15 H12 O5	6.354		272.0685	0.07		FBF	93.59	FBF

Species	m/z	Score (Tgt)	Score (Lib)	Score (DB)	Score (MFG)	Score (RT)
(M+NH4)+	290.1020	93.59				

Compound Chromatograms (overlaid)

Structure

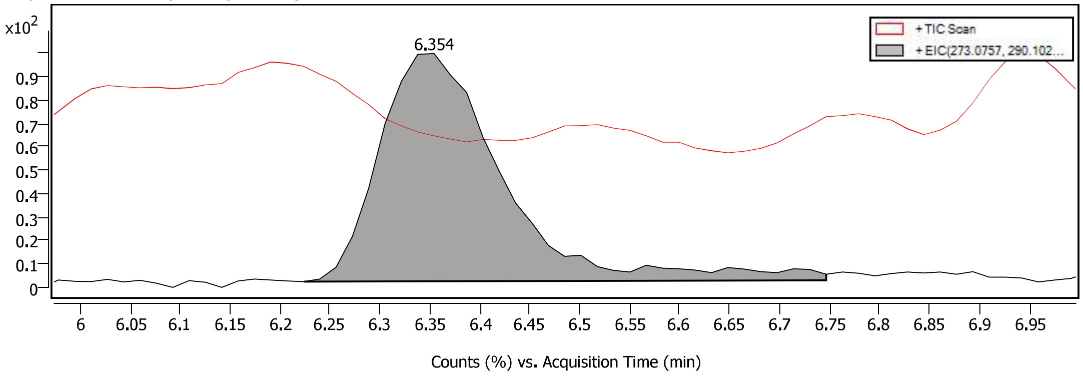

Compound Spectra (overlaid)

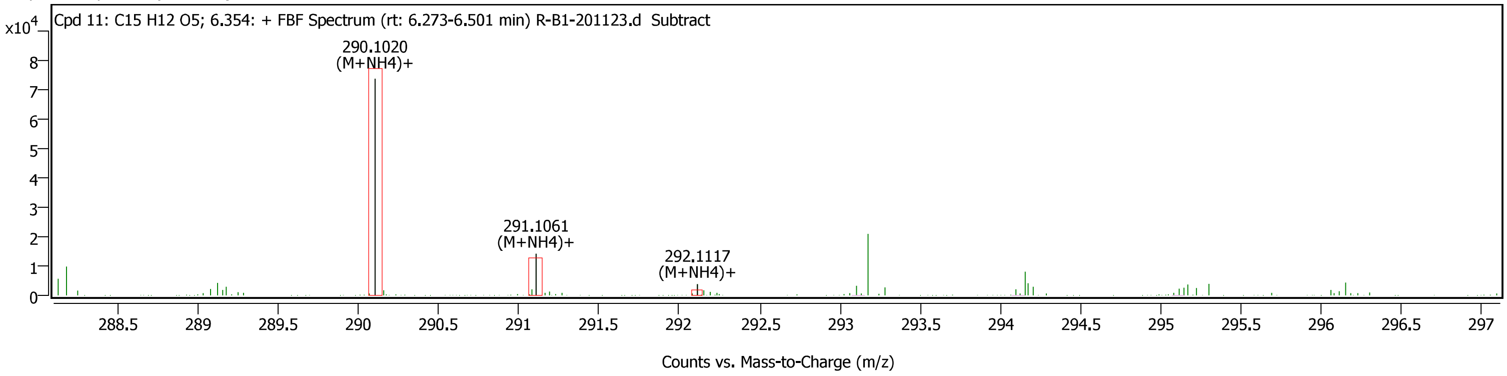

Compound ID Table

Name	Formula	Species	RT	RT Diff	Mass	CAS	ID Source	Score	Score (Lib)	Score (Tgt)
	C15 H12 O5	(M+NH4)+	6.354		272.0685		FBF	93.59		93.59

Cpd. 12: C27 H30 O16

Name	Formula	RT	RI	Mass	Diff (Tgt, ppm)	CAS	ID Source	Score	Algorithm
	C27 H30 O16	3.084		610.1599	10.71		FBF	18.87	FBF

Species	m/z	Score (Tgt)	Score (Lib)	Score (DB)	Score (MFG)	Score (RT)
(M+H)+ (M+NH4)+ (M+Na)+	611.1723 628.1971 633.1299	18.87				

Compound Chromatograms (overlaid)

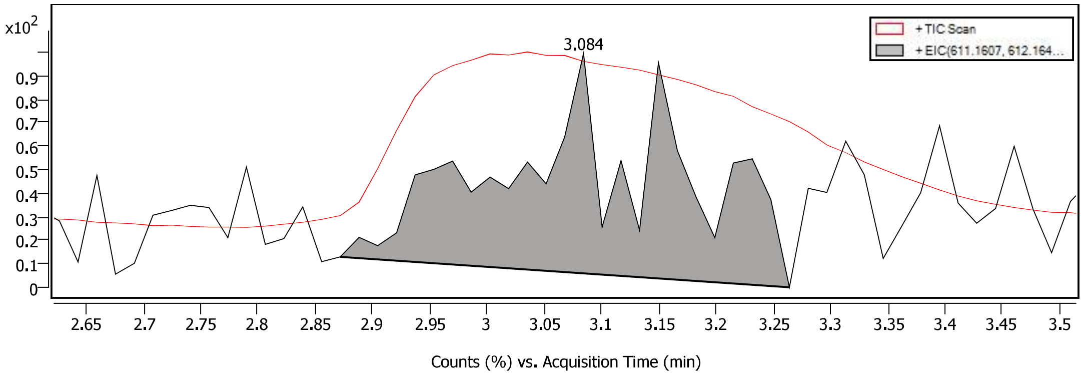

Structure

Compound Spectra (overlaid)

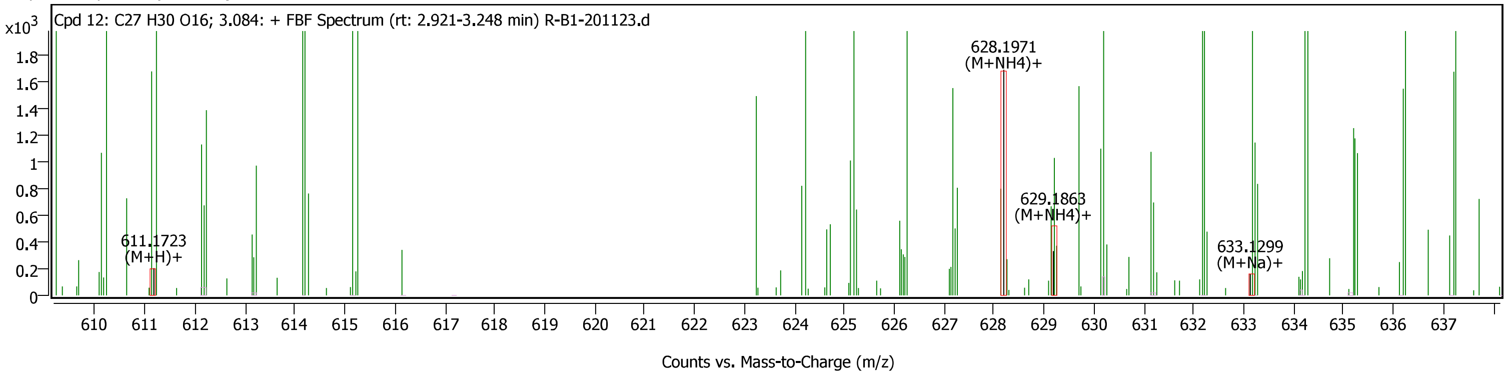

Compound ID Table

Name	Formula	Species	RT	RT Diff	Mass	CAS	ID Source	Score	Score (Lib)	Score (Tgt)
	C27 H30 O16	(M+H)+ (M+NH4)+ (M+Na)+	3.084		610.1599		FBF	18.87		18.87

Cpd. 13: C9 H10 O4

Name	Formula	RT	RI	Mass	Diff (Tgt, ppm)	CAS	ID Source	Score	Algorithm
	C9 H10 O4	2.496		182.0599	11.17		FBF	79.17	FBF

Species	m/z	Score (Tgt)	Score (Lib)	Score (DB)	Score (MFG)	Score (RT)
(M+H)+ (M+NH4)+ (M+Na)+	183.0626 200.0915 205.0495	79.17				

Compound Chromatograms (overlaid)

Structure

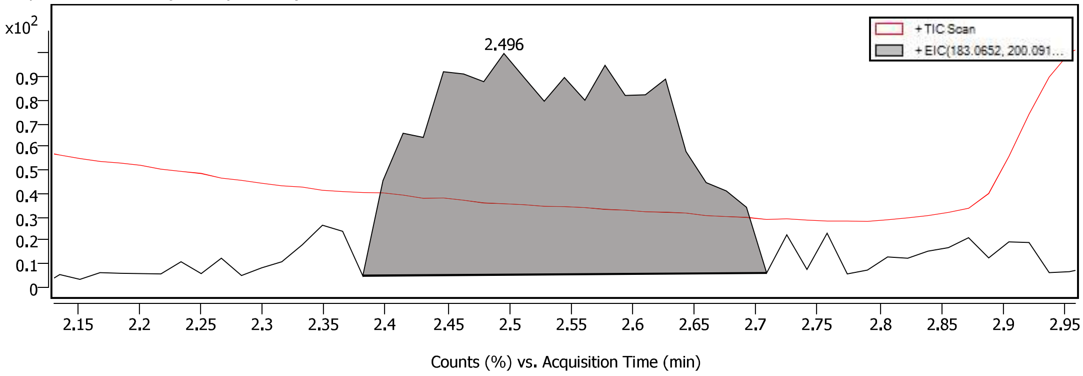

Compound Spectra (overlaid)

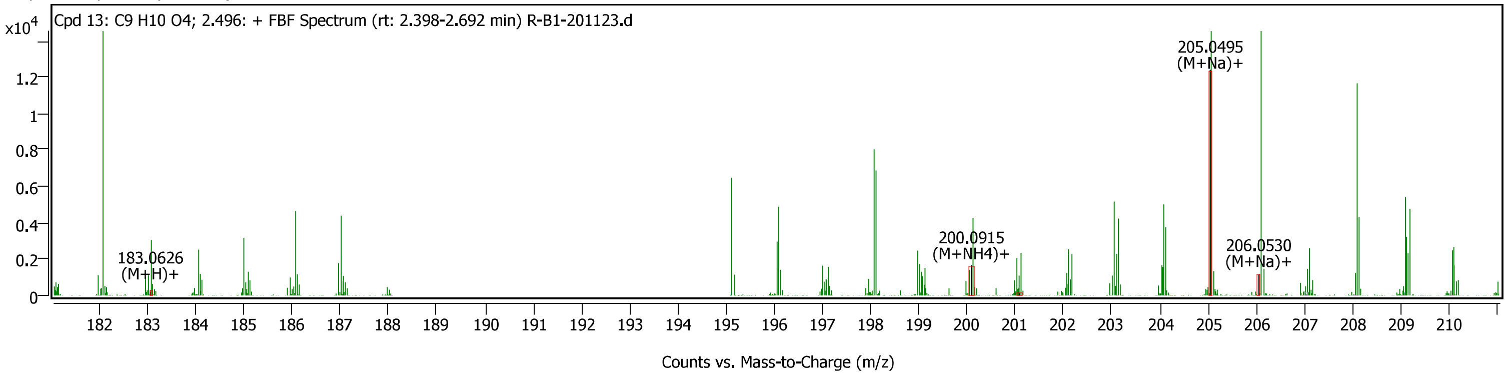

Compound ID Table

Name	Formula	Species	RT	RT Diff	Mass	CAS	ID Source	Score	Score (Lib)	Score (Tgt)
	C9 H10 O4	(M+H)+<br>(M+NH4)+<br>(M+Na)+	2.496		182.0599		FBF	79.17		79.17

Cpd. 14: C11 H12 O5

Name	Formula	RT	RI	Mass	Diff (Tgt, ppm)	CAS	ID Source	Score	Algorithm
	C11 H12 O5	4.605		224.0672	-5.61		FBF	80.89	FBF

Species	m/z	Score (Tgt)	Score (Lib)	Score (DB)	Score (MFG)	Score (RT)
(M+H)+ (M+NH4)+	225.0741 242.1012	80.89				

Compound Chromatograms (overlaid)

Structure

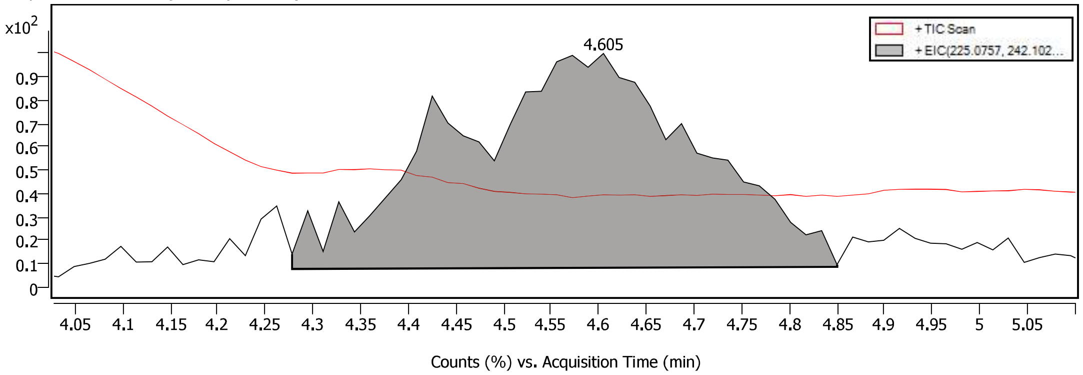

Target Screening Report

Compound Spectra (overlaid)

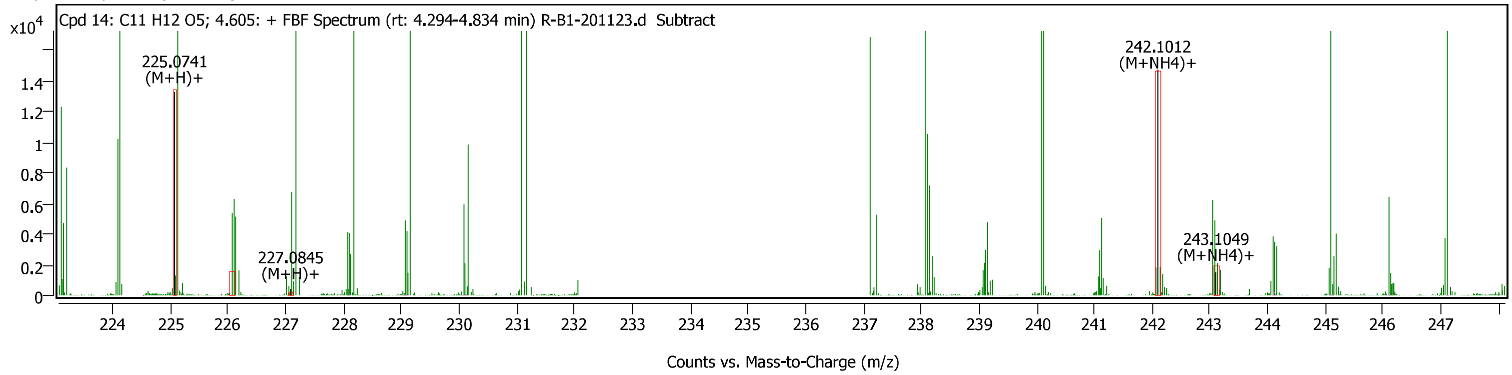

Compound ID Table

Name	Formula	Species	RT	RT Diff	Mass	CAS	ID Source	Score	Score (Lib)	Score (Tgt)
	C11 H12 O5	(M+H)+ (M+NH4)+	4.605		224.0672		FBF	80.89		80.89

Cpd. 15: C16 H12 O7

Name	Formula	RT	RI	Mass	Diff (Tgt, ppm)	CAS	ID Source	Score	Algorithm
	C16 H12 O7	7.123		316.0575	-2.40		FBF	83.41	FBF

Species	m/z	Score (Tgt)	Score (Lib)	Score (DB)	Score (MFG)	Score (RT)
(M+H)+ (M+NH4)+ (M+Na)+	317.0651 334.0886 339.0438	83.41				

Compound Chromatograms (overlaid)

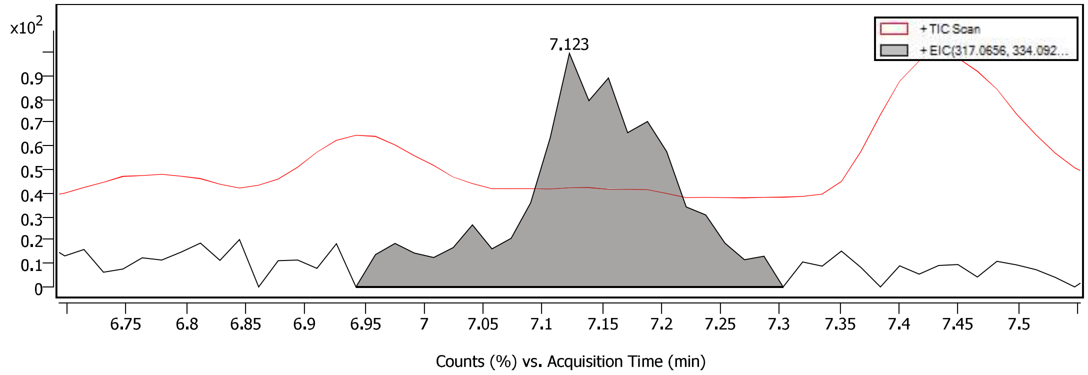

Structure

Compound Spectra (overlaid)

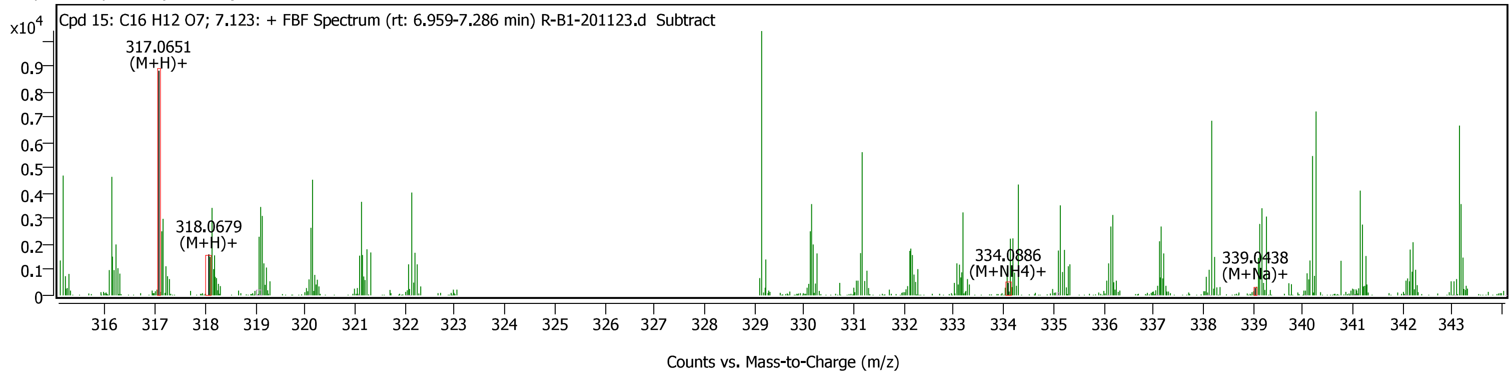

Compound ID Table

Name	Formula	Species	RT	RT Diff	Mass	CAS	ID Source	Score	Score (Lib)	Score (Tgt)
	C16 H12 O7	(M+H)+ (M+NH4)+ (M+Na)+	7.123		316.0575		FBF	83.41		83.41

Cpd. 16: C15 H10 O4

Name	Formula	RT	RI	Mass	Diff (Tgt, ppm)	CAS	ID Source	Score	Algorithm
	C15 H10 O4	10.311		254.0570	-3.52		FBF	78.91	FBF

Species	m/z	Score (Tgt)	Score (Lib)	Score (DB)	Score (MFG)	Score (RT)
(M+H)+ (M+NH4)+	255.0643 272.0909	78.91				

Target Screening Report

Compound Chromatograms (overlaid)

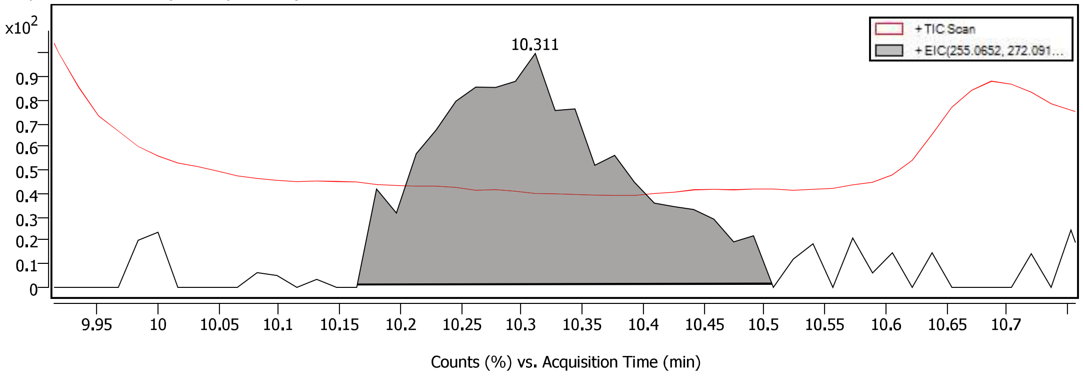

Structure

Compound Spectra (overlaid)

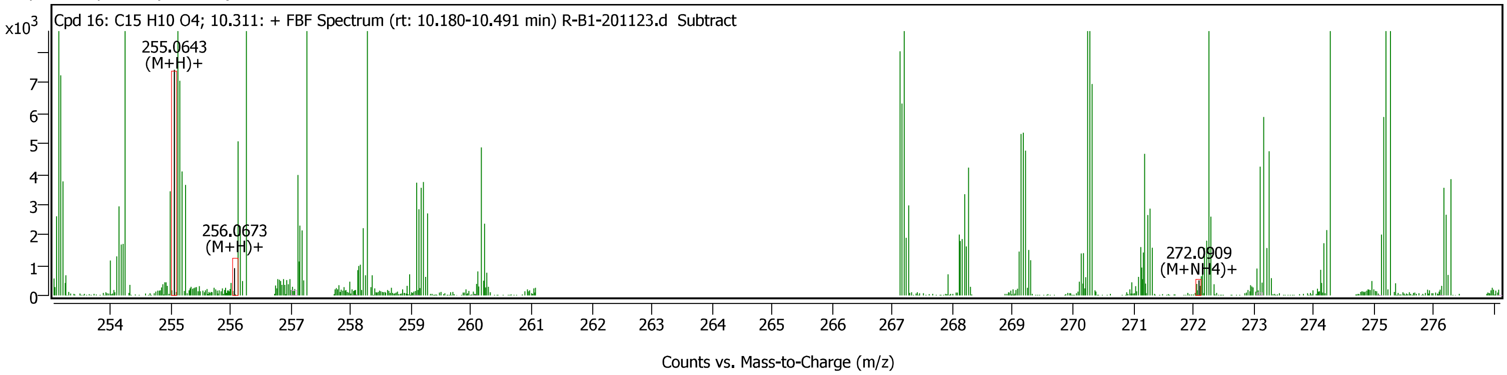

Compound ID Table

Name	Formula	Species	RT	RT Diff	Mass	CAS	ID Source	Score	Score (Lib)	Score (Tgt)
	C15 H10 O4	(M+H)+ (M+NH4)+	10.311		254.0570		FBF	78.91		78.91

Cpd. 17: C15 H12 O4

Name	Formula	RT	RI	Mass	Diff (Tgt, ppm)	CAS	ID Source	Score	Algorithm
	C15 H12 O4	6.861		256.0731	-1.87		FBF	59.38	FBF

Species	m/z	Score (Tgt)	Score (Lib)	Score (DB)	Score (MFG)	Score (RT)
(M+H)+ (M+NH4)+	257.0802 274.1069	59.38				

Compound Chromatograms (overlaid)

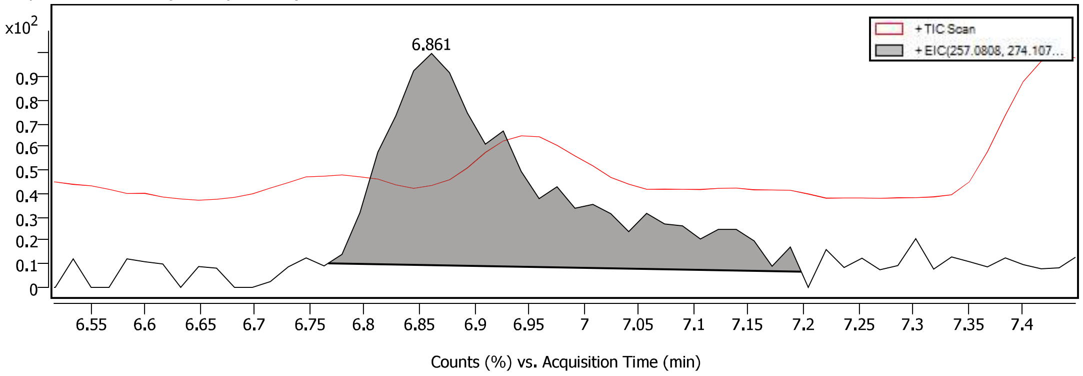

Structure

Compound Spectra (overlaid)

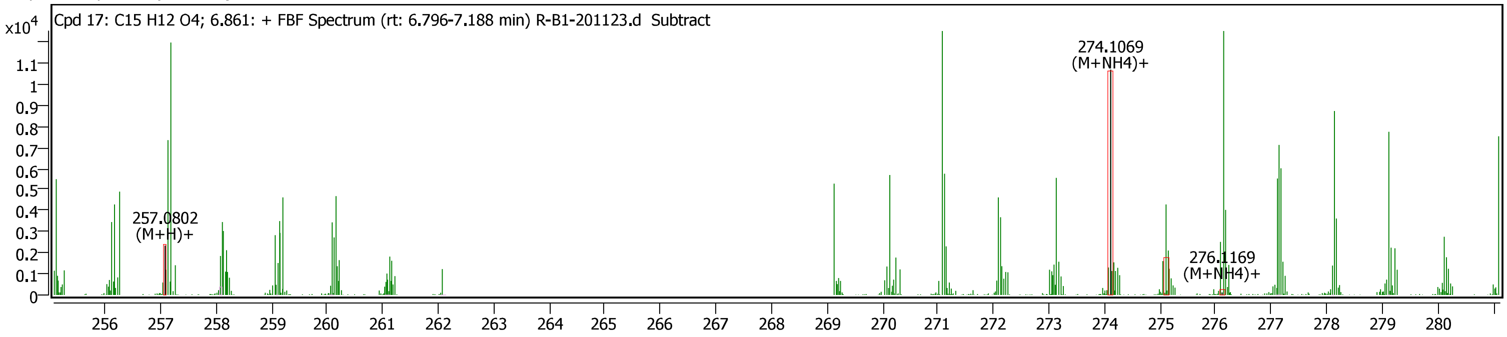

Compound ID Table

Name	Formula	Species	RT	RT Diff	Mass	CAS	ID Source	Score	Score (Lib)	Score (Tgt)
	C15 H12 O4	(M+H)+ (M+NH4)+	6.861		256.0731		FBF	59.38		59.38

Cpd. 18: C9 H8 O4

Name	Formula	RT	RI	Mass	Diff (Tgt, ppm)	CAS	ID Source	Score	Algorithm
	C9 H8 O4	1.106		180.0425	1.24		FBF	78.57	FBF

Species	m/z	Score (Tgt)	Score (Lib)	Score (DB)	Score (MFG)	Score (RT)
(M+H)+ (M+NH4)+	181.0495 198.0766	78.57				

Compound Chromatograms (overlaid)

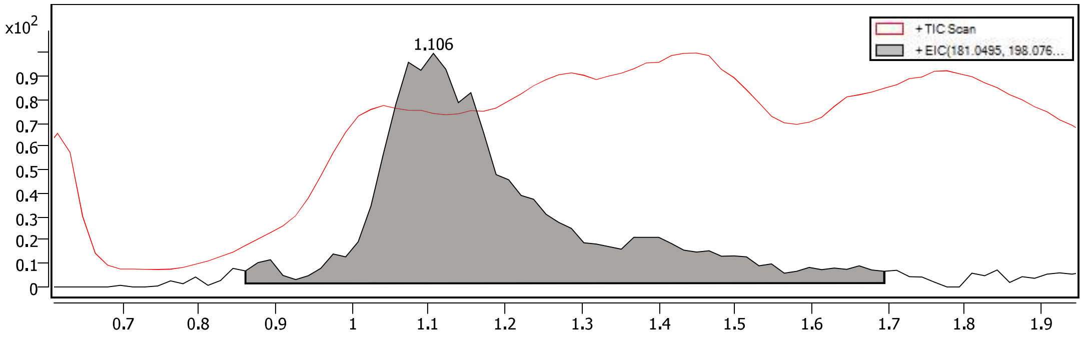

Structure

Compound Spectra (overlaid)

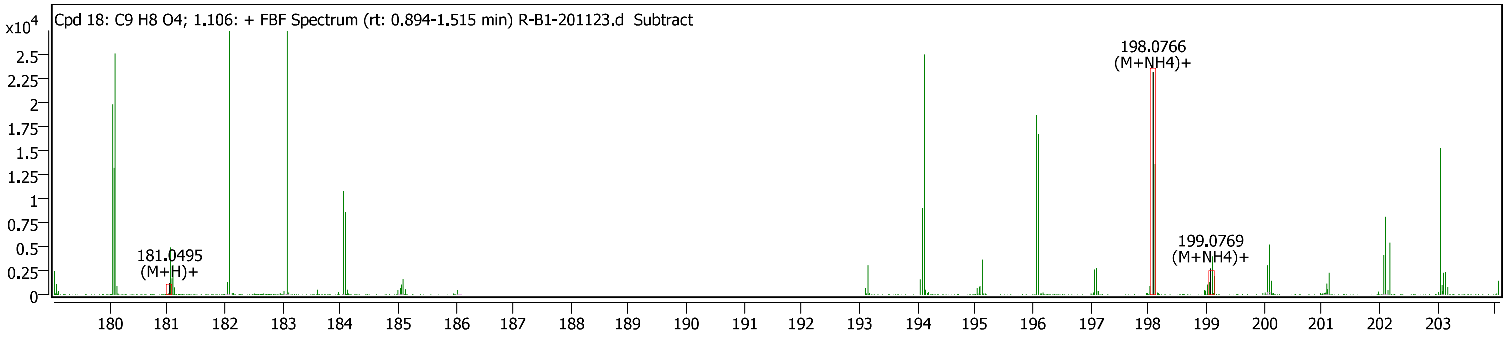

Compound ID Table

Name	Formula	Species	RT	RT Diff	Mass	CAS	ID Source	Score	Score (Lib)	Score (Tgt)
	C9 H8 O4	(M+H)+ (M+NH4)+	1.106		180.0425		FBF	78.57		78.57

Cpd. 19: C16 H18 O9

Name	Formula	RT	RI	Mass	Diff (Tgt, ppm)	CAS	ID Source	Score	Algorithm
	C16 H18 O9	5.749		354.0915	-10.05		FBF	21.47	FBF

Species	m/z	Score (Tgt)	Score (Lib)	Score (DB)	Score (MFG)	Score (RT)
(M+H)+	355.0988	21.47				

Compound Chromatograms (overlaid)

Structure

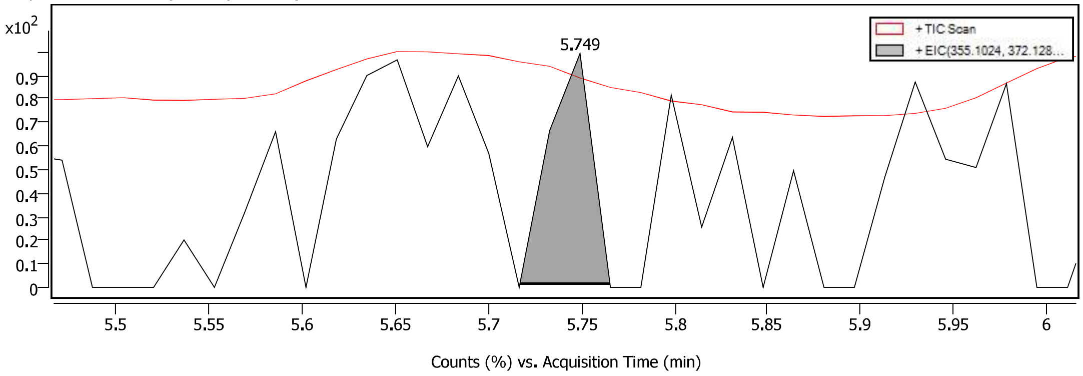

Compound Spectra (overlaid)

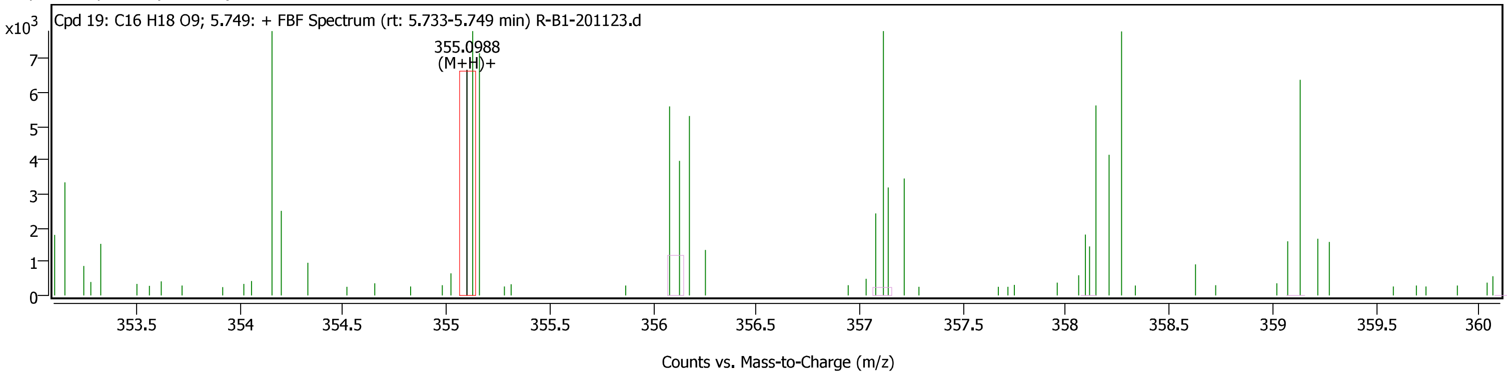

Compound ID Table

Name	Formula	Species	RT	RT Diff	Mass	CAS	ID Source	Score	Score (Lib)	Score (Tgt)
	C16 H18 O9	(M+H)+	5.749		354.0915		FBF	21.47		21.47

Cpd. 20: C8 H8 O4

Name	Formula	RT	RI	Mass	Diff (Tgt, ppm)	CAS	ID Source	Score	Algorithm
	C8 H8 O4	1.891		168.0420	-1.34		FBF	83.02	FBF

Species	m/z	Score (Tgt)	Score (Lib)	Score (DB)	Score (MFG)	Score (RT)
(M+H)+ (M+NH4)+	169.0482 186.0760	83.02				

Compound Chromatograms (overlaid)

Structure

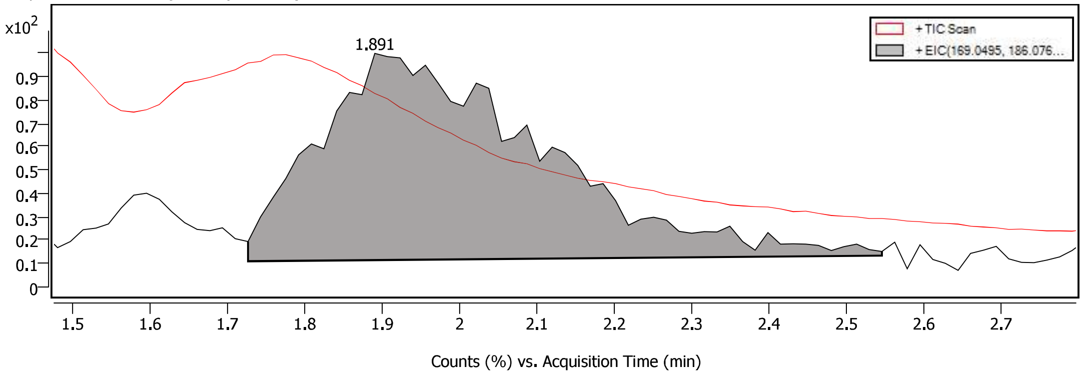

Compound Spectra (overlaid)

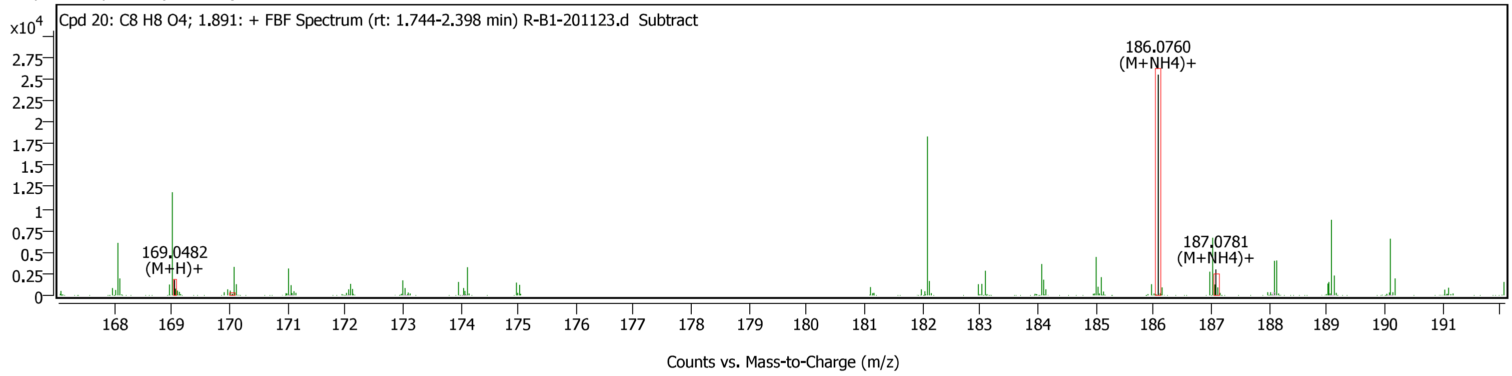

Compound ID Table

Name	Formula	Species	RT	RT Diff	Mass	CAS	ID Source	Score	Score (Lib)	Score (Tgt)
	C8 H8 O4	(M+H)+ (M+NH4)+	1.891		168.0420		FBF	83.02		83.02

Cpd. 21: C7 H5 O2

Name	Formula	RT	RI	Mass	Diff (Tgt, ppm)	CAS	ID Source	Score	Algorithm
	C7 H5 O2	7.482		121.0328	32.03		FBF	7.06	FBF

Species	m/z	Score (Tgt)	Score (Lib)	Score (DB)	Score (MFG)	Score (RT)
(M+Na)+	144.0221	7.06				

Compound Chromatograms (overlaid)

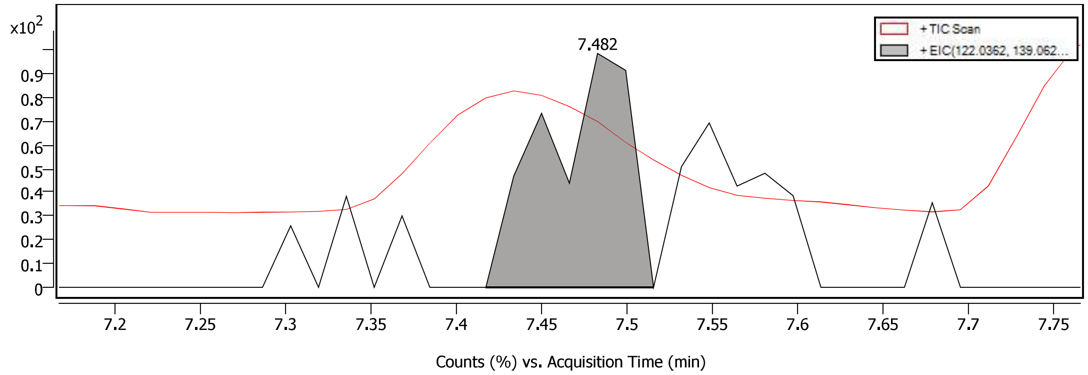

Structure

Compound Spectra (overlaid)

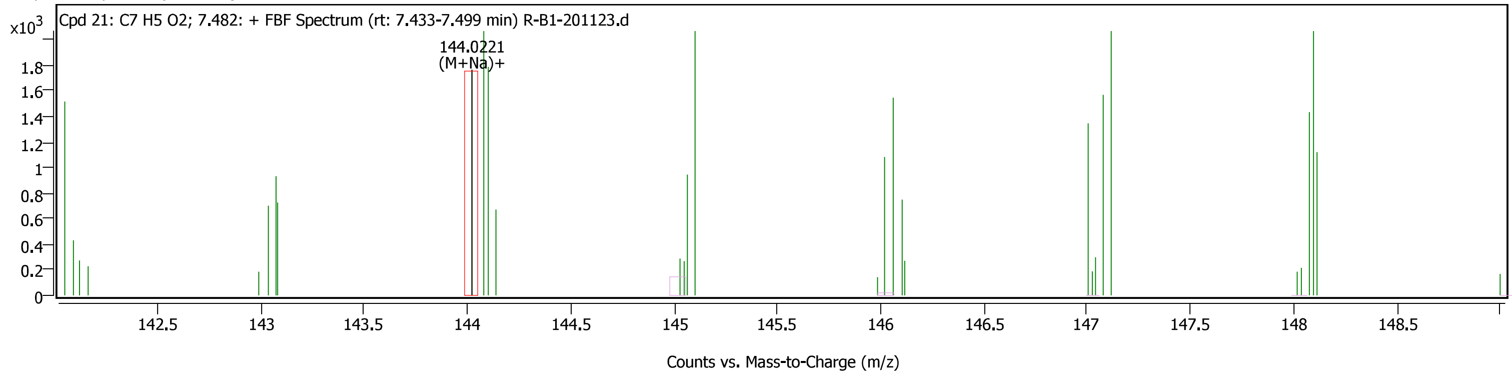

Compound ID Table

Name	Formula	Species	RT	RT Diff	Mass	CAS	ID Source	Score	Score (Lib)	Score (Tgt)
	C7 H5 O2	(M+Na)+	7.482		121.0328		FBF	7.06		7.06

Cpd. 22: C14 H6 O8

Name	Formula	RT	RI	Mass	Diff (Tgt, ppm)	CAS	ID Source	Score	Algorithm
	C14 H6 O8	0.616		302.0068	1.92		FBF	46.10	FBF

Species	m/z	Score (Tgt)	Score (Lib)	Score (DB)	Score (MFG)	Score (RT)
(M+H)+ (M+NH4)+	303.0177 320.0317	46.10				

Compound Chromatograms (overlaid)

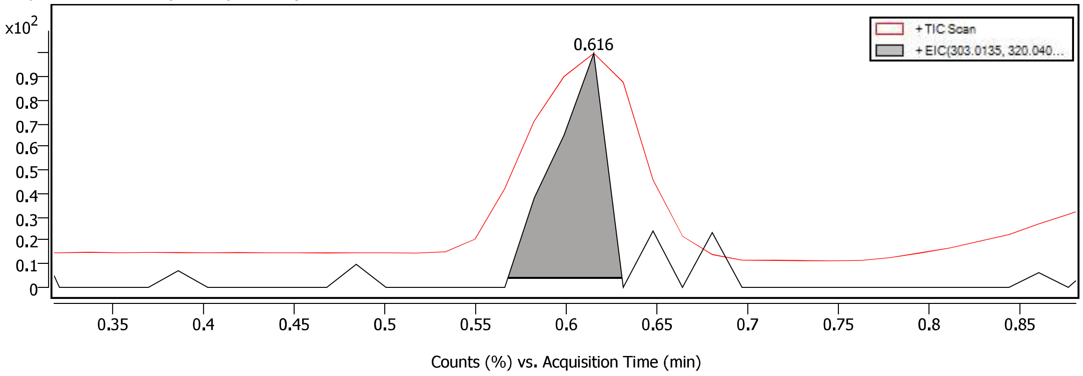

Structure

Compound Spectra (overlaid)

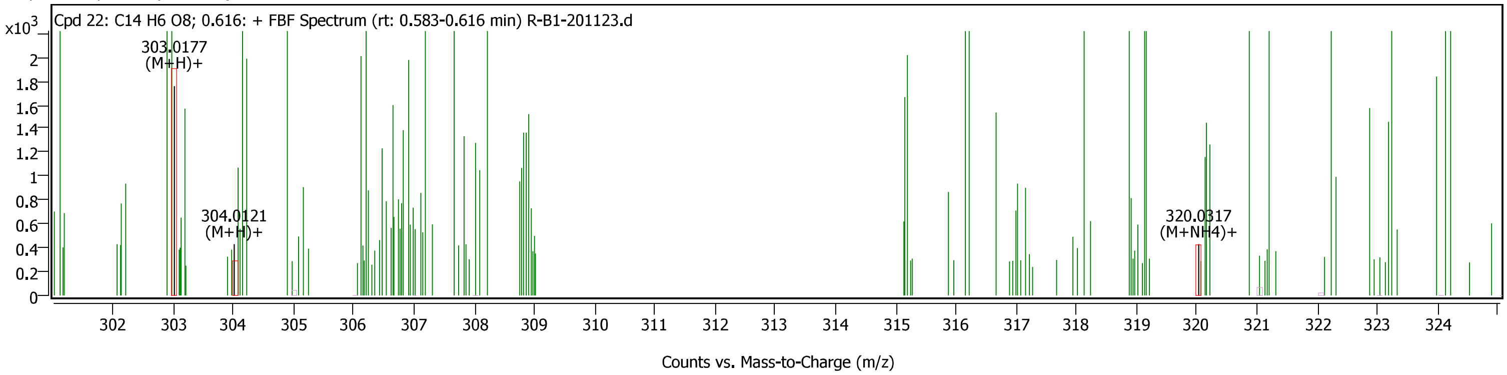

Compound ID Table

Name	Formula	Species	RT	RT Diff	Mass	CAS	ID Source	Score	Score (Lib)	Score (Tgt)
	C14 H6 O8	(M+H)+ (M+NH4)+	0.616		302.0068		FBF	46.10		46.10

Cpd. 23: C9 H10 O5

Name	Formula	RT	RI	Mass	Diff (Tgt, ppm)	CAS	ID Source	Score	Algorithm
	C9 H10 O5	4.736		198.0535	3.27		FBF	79.75	FBF

Species	m/z	Score (Tgt)	Score (Lib)	Score (DB)	Score (MFG)	Score (RT)
(M+H)+ (M+NH4)+ (M+Na)+	199.0599 216.0877 221.0437	79.75				

Compound Chromatograms (overlaid)

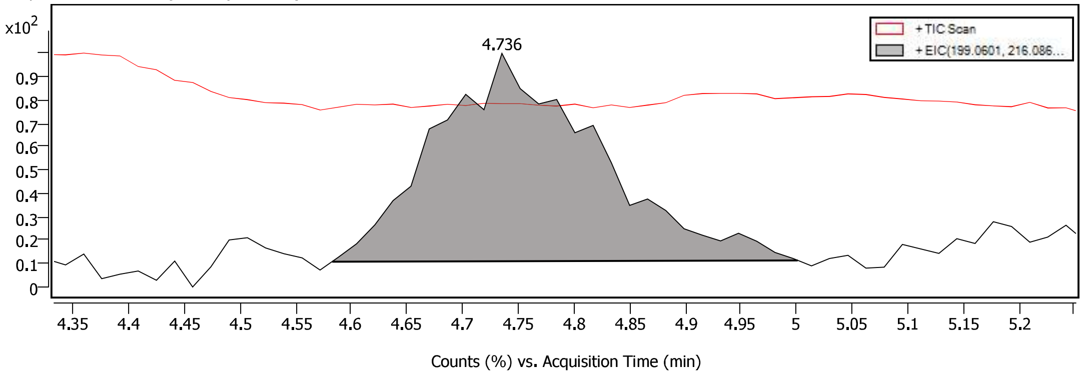

Structure

Compound Spectra (overlaid)

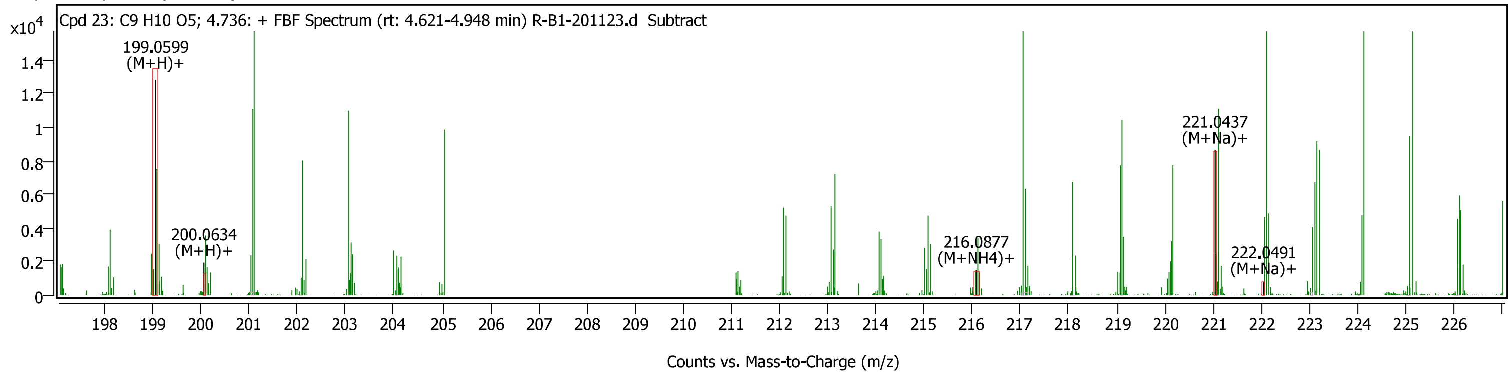

Compound ID Table

Name	Formula	Species	RT	RT Diff	Mass	CAS	ID Source	Score	Score (Lib)	Score (Tgt)
	C9 H10 O5	(M+H)+ (M+NH4)+ (M+Na)+	4.736		198.0535		FBF	79.75		79.75

Cpd. 24: C10 H10 O4

Name	Formula	RT	RI	Mass	Diff (Tgt, ppm)	CAS	ID Source	Score	Algorithm
	C10 H10 O4	2.610		194.0577	-0.92		FBF	78.45	FBF

Species	m/z	Score (Tgt)	Score (Lib)	Score (DB)	Score (MFG)	Score (RT)
(M+H)+ (M+NH4)+ (M+Na)+	195.0667 212.0917 217.0428	78.45				

Compound Chromatograms (overlaid)

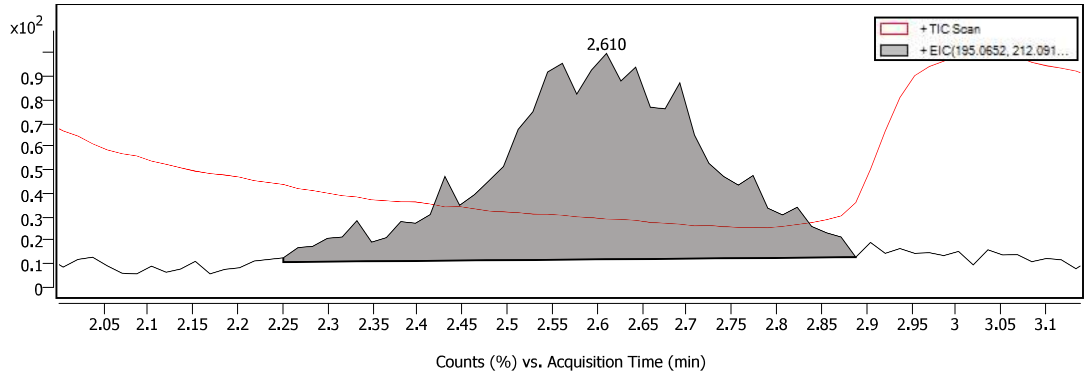

Structure

Compound Spectra (overlaid)

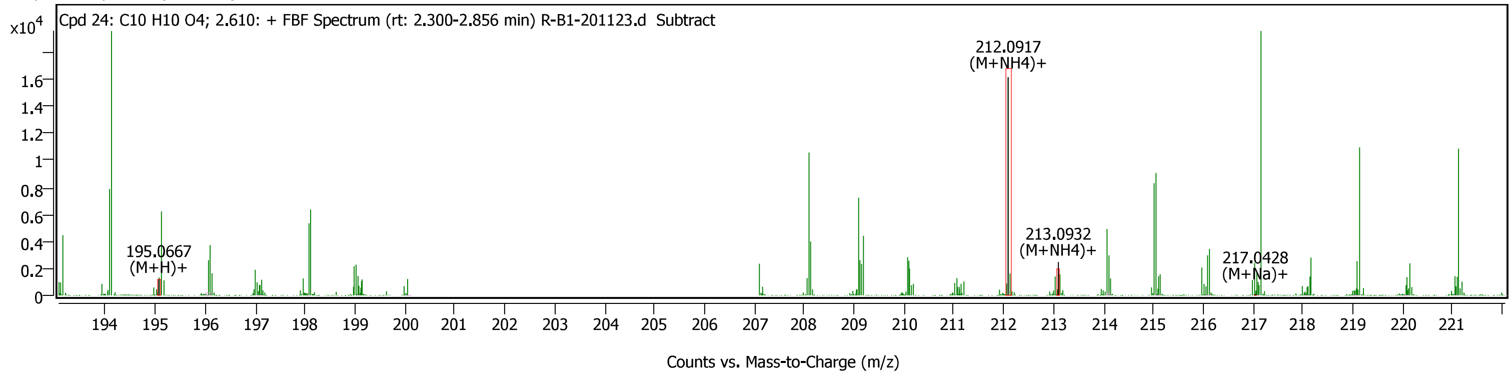

Compound ID Table

Name	Formula	Species	RT	RT Diff	Mass	CAS	ID Source	Score	Score (Lib)	Score (Tgt)
	C10 H10 O4	(M+H)+ (M+NH4)+ (M+Na)+	2.610		194.0577		FBF	78.45		78.45

Cpd. 25: C7 H6 O4

Name	Formula	RT	RI	Mass	Diff (Tgt, ppm)	CAS	ID Source	Score	Algorithm
	C7 H6 O4	0.975		154.0264	-1.35		FBF	83.63	FBF

Species	m/z	Score (Tgt)	Score (Lib)	Score (DB)	Score (MFG)	Score (RT)
(M+H)+ (M+NH4)+	155.0330 172.0604	83.63				

Target Screening Report

Compound Chromatograms (overlaid)

Structure

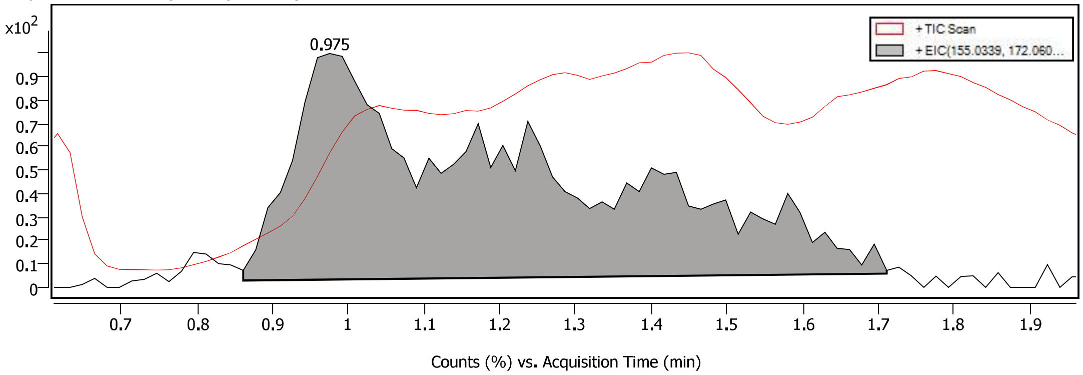

Compound Spectra (overlaid)

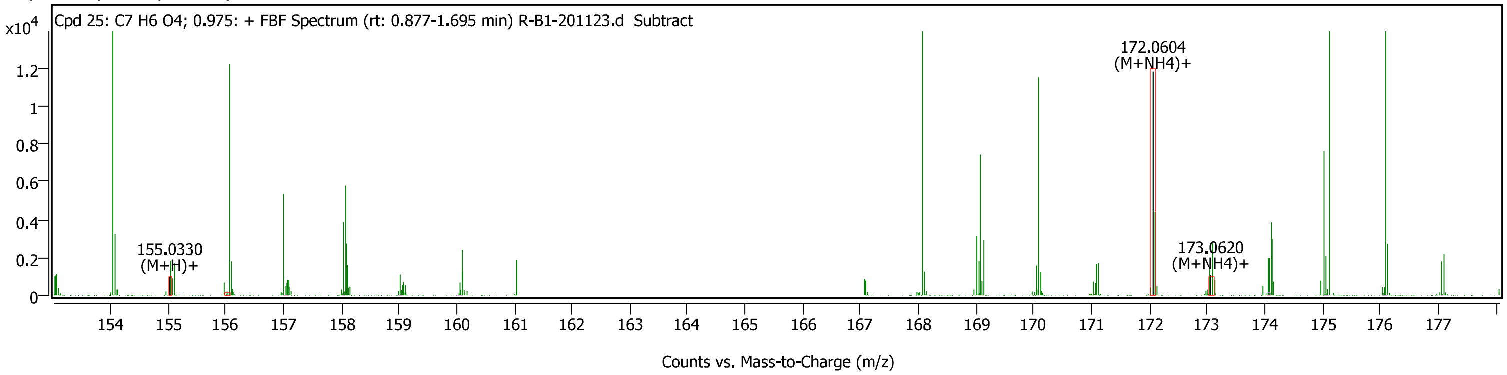

Compound ID Table

Name	Formula	Species	RT	RT Diff	Mass	CAS	ID Source	Score	Score (Lib)	Score (Tgt)
	C7 H6 O4	(M+H)+ (M+NH4)+	0.975		154.0264		FBF	83.63		83.63

Cpd. 26: C16 H14 O6

Name	Formula	RT	RI	Mass	Diff (Tgt, ppm)	CAS	ID Source	Score	Algorithm
	C16 H14 O6	6.632		302.0774	-5.56		FBF	58.98	FBF

Species	m/z	Score (Tgt)	Score (Lib)	Score (DB)	Score (MFG)	Score (RT)
(M+H)+ (M+NH4)+	303.0858 320.1115	58.98				

Compound Chromatograms (overlaid)

Structure

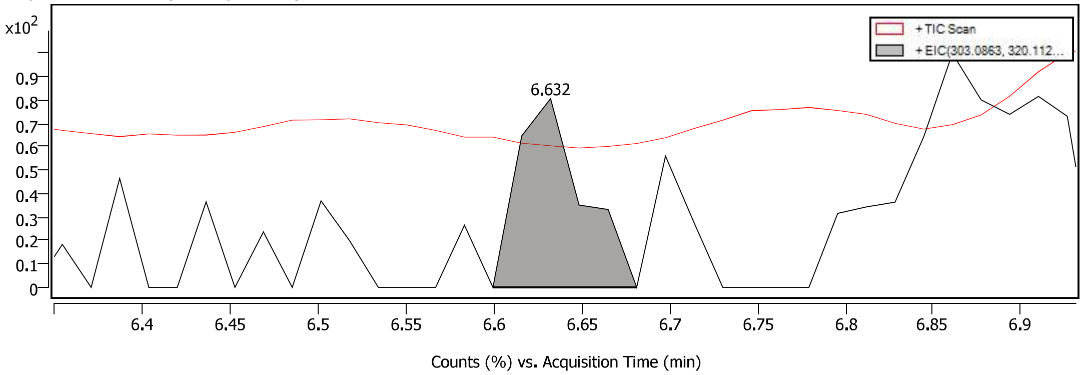

Compound Spectra (overlaid)

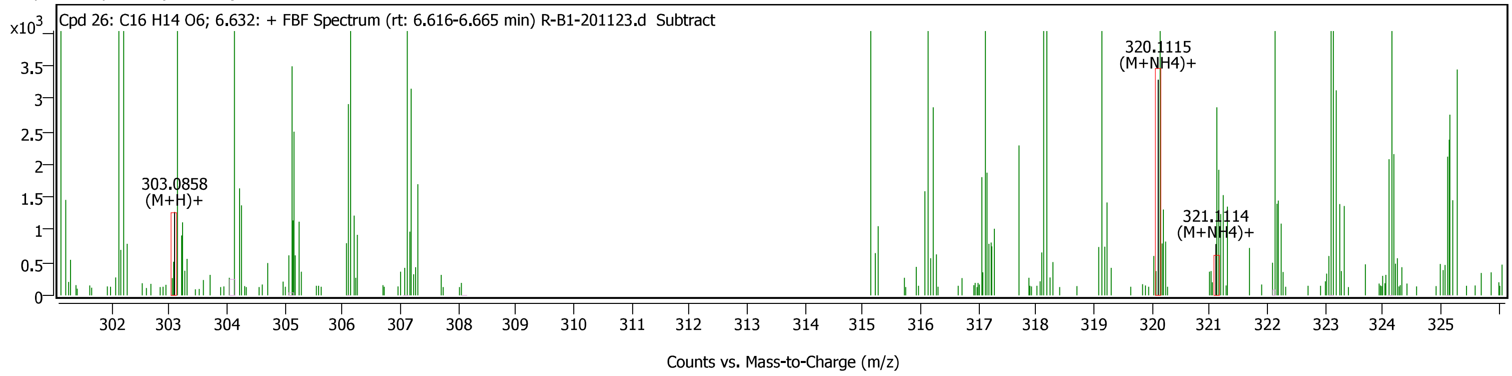

Compound ID Table

Name	Formula	Species	RT	RT Diff	Mass	CAS	ID Source	Score	Score (Lib)	Score (Tgt)
	C16 H14 O6	(M+H)+ (M+NH4)+	6.632		302.0774		FBF	58.98		58.98

Cpd. 27: C15 H10 O5

Name	Formula	RT	RI	Mass	Diff (Tgt, ppm)	CAS	ID Source	Score	Algorithm
	C15 H10 O5	5.733		270.0535	2.34		FBF	46.18	FBF

Species	m/z	Score (Tgt)	Score (Lib)	Score (DB)	Score (MFG)	Score (RT)
(M+NH4)+	288.0873	46.18				

Compound Chromatograms (overlaid)

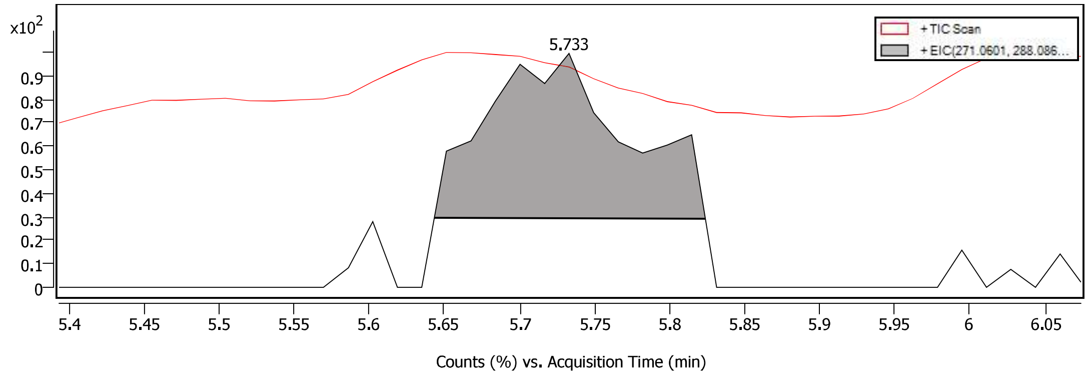

Structure

Compound Spectra (overlaid)

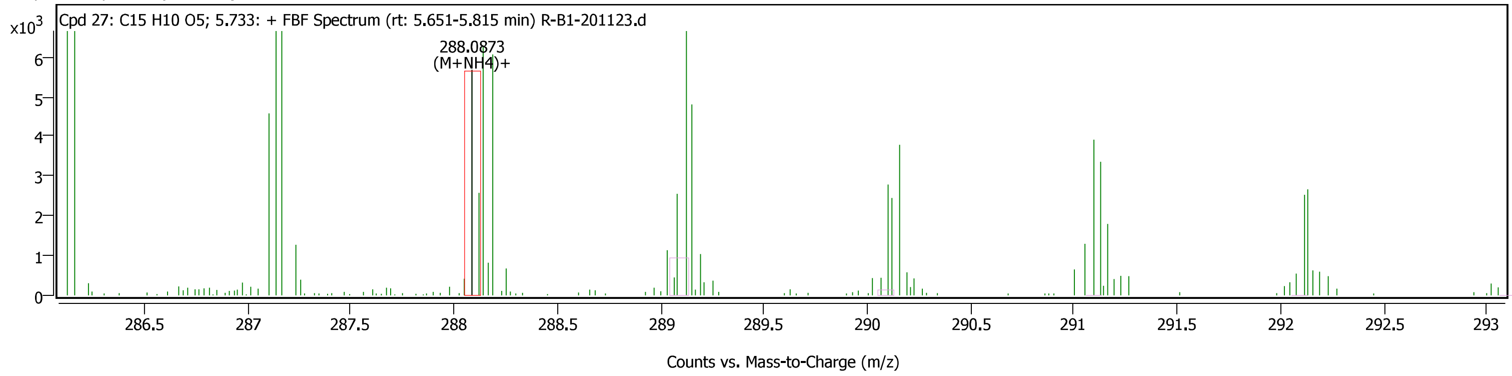

Compound ID Table

Name	Formula	Species	RT	RT Diff	Mass	CAS	ID Source	Score	Score (Lib)	Score (Tgt)
	C15 H10 O5	(M+NH4)+	5.733		270.0535		FBF	46.18		46.18

Cpd. 28: C15 H14 O6

Name	Formula	RT	RI	Mass	Diff (Tgt, ppm)	CAS	ID Source	Score	Algorithm
	C15 H14 O6	1.466		290.0786	-1.56		FBF	95.97	FBF

Species	m/z	Score (Tgt)	Score (Lib)	Score (DB)	Score (MFG)	Score (RT)
(M+H)+ (M+NH4)+	291.0903 308.1121	95.97				

Target Screening Report

Compound Chromatograms (overlaid)

Structure

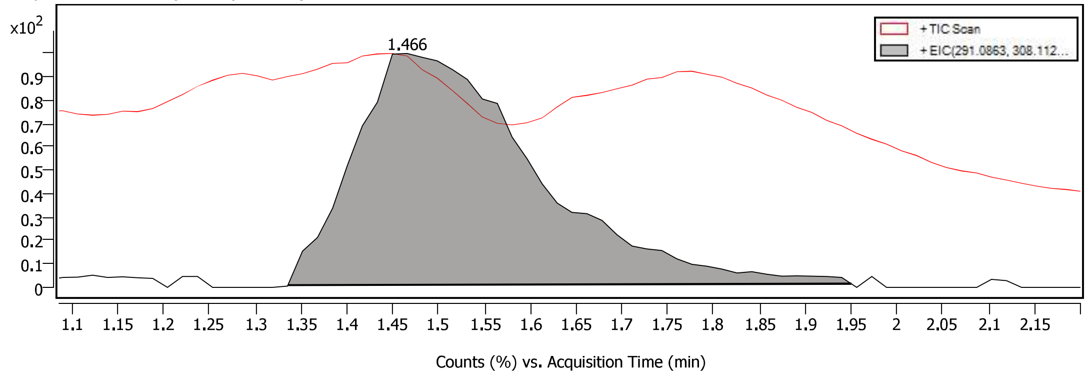

Compound Spectra (overlaid)

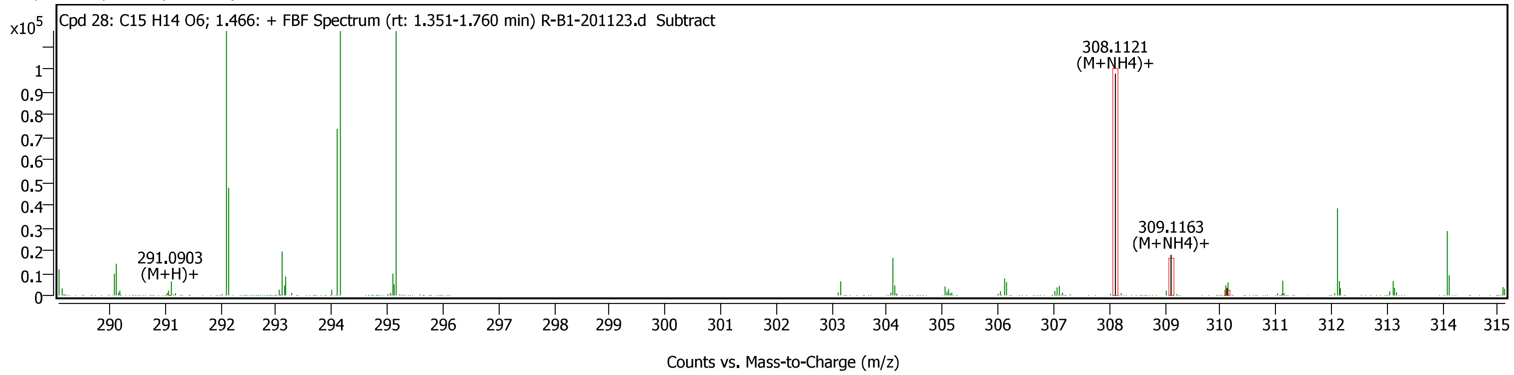

Compound ID Table

Name	Formula	Species	RT	RT Diff	Mass	CAS	ID Source	Score	Score (Lib)	Score (Tgt)
	C15 H14 O6	(M+H)+ (M+NH4)+	1.466		290.0786		FBF	95.97		95.97

Cpd. 29: C15 H10 O6

Name	Formula	RT	RI	Mass	Diff (Tgt, ppm)	CAS	ID Source	Score	Algorithm
	C15 H10 O6	1.220		286.0444	-11.79		FBF	54.57	FBF

Species	m/z	Score (Tgt)	Score (Lib)	Score (DB)	Score (MFG)	Score (RT)
(M+H)+ (M+NH4)+ (M+Na)+	287.0519 304.0732 309.0409	54.57				

Compound Chromatograms (overlaid)

Structure

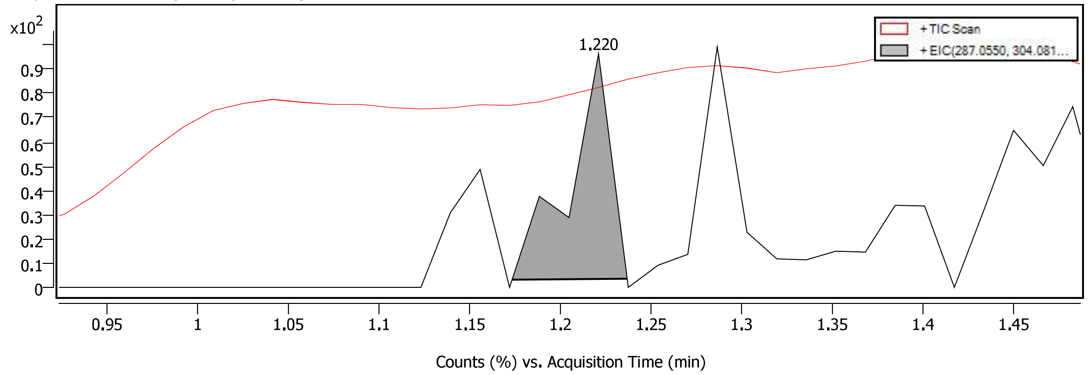

Compound Spectra (overlaid)

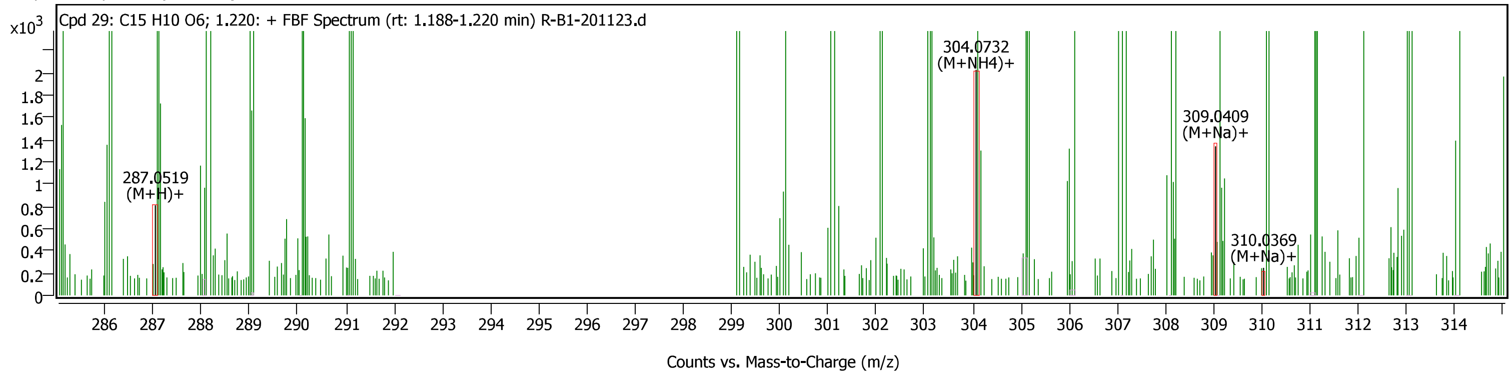

Compound ID Table

Name	Formula	Species	RT	RT Diff	Mass	CAS	ID Source	Score	Score (Lib)	Score (Tgt)
	C15 H10 O6	(M+H)+ (M+NH4)+ (M+Na)+	1.220		286.0444		FBF	54.57		54.57

Cpd. 30: C15 H10 O7

Name	Formula	RT	RI	Mass	Diff (Tgt, ppm)	CAS	ID Source	Score	Algorithm
	C15 H10 O7	4.572		302.0440	4.38		FBF	67.67	FBF

Species	m/z	Score (Tgt)	Score (Lib)	Score (DB)	Score (MFG)	Score (RT)
(M+H)+ (M+NH4)+ (M+Na)+	303.0409 320.0772 325.0337	67.67				

Compound Chromatograms (overlaid)

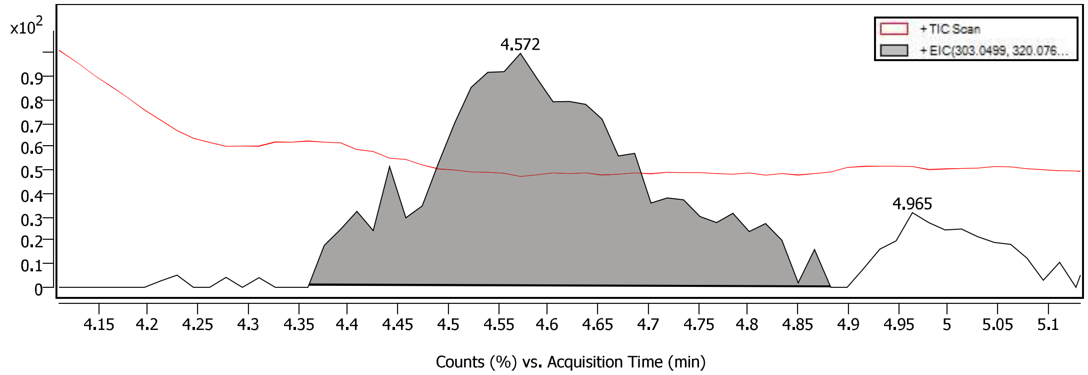

Structure

Compound Spectra (overlaid)

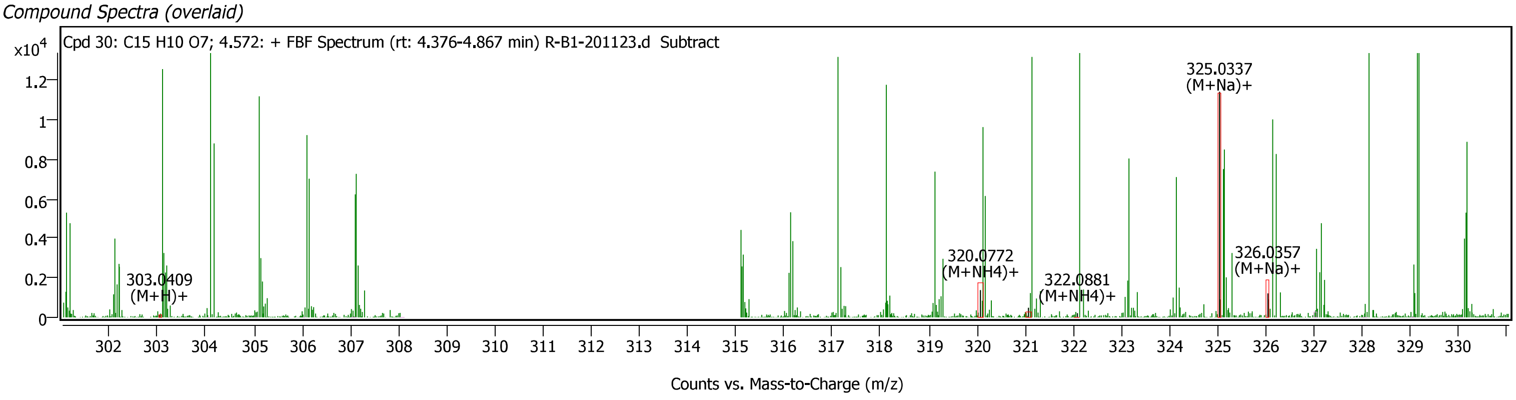

Compound ID Table

Name	Formula	Species	RT	RT Diff	Mass	CAS	ID Source	Score	Score (Lib)	Score (Tgt)
	C15 H10 O7	(M+H)+ (M+NH4)+ (M+Na)+	4.572		302.0440		FBF	67.67		67.67

MassHunter Qual 10.0
(End of Report)
