# Supplementary material for: Physicochemical and antioxidant properties of Apis cerana honey from Lombok and Bali Islands
Source: PLoS One. 2024 Apr 5;19(4):e0301213. doi: 10.1371/journal.pone.0301213 (PMC10997079; doi:10.1371/journal.pone.0301213)
Supplement: S2 Appendix — (PDF) [file pone.0301213.s002.pdf]

Target Screening Report

Sample Information

**Name**	Sample R-B3-201123	**Data File Path**	D:\MassHunter\Data\Saeed Ullah\R-B3-201123.d
**Sample ID**	Bali honey	**Acq. Time (Local)**	11/20/2023 12:30:07 PM (UTC+08:00)
**Instrument**	Instrument 1	**Method Path (Acq)**	D:\MassHunter\Methods\Training Method 210822.m
**MS Type**	QTOF	**Version (Acq SW)**	6200 series TOF/6500 series Q-TOF B.09.00 (B9044.1 SP1)
**Inj. Vol. (ul)**	1	**IRM Status**	All ions missed
**Position**	P1-A3	**Method Path (DA)**	D:\MassHunter\Methods\10.0\Default-LCMS.m
**Plate Pos.**		**Target Source Path**	
**Operator**		**Result Summary**	21 qualified (30 targets)

Sample Chromatograms

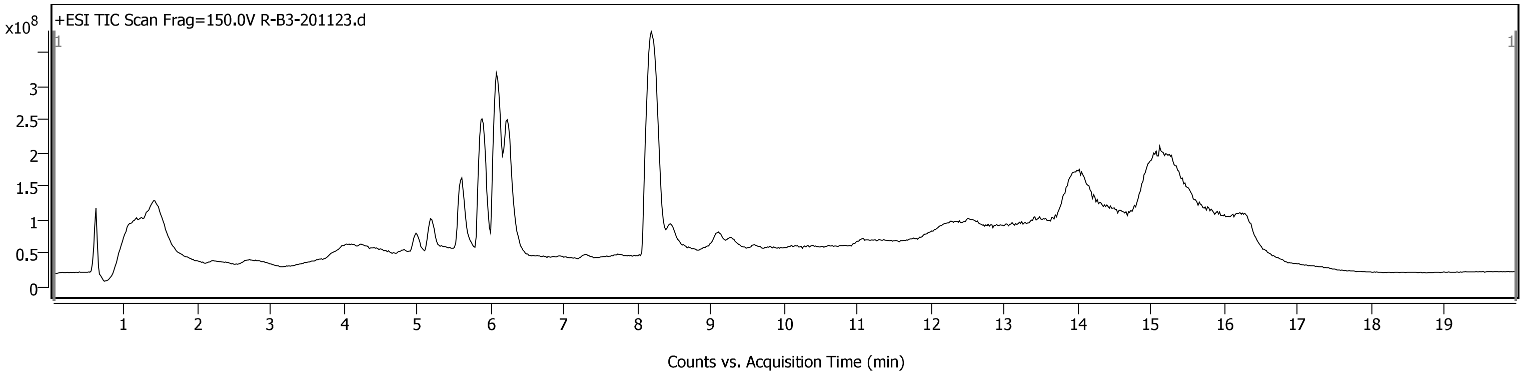

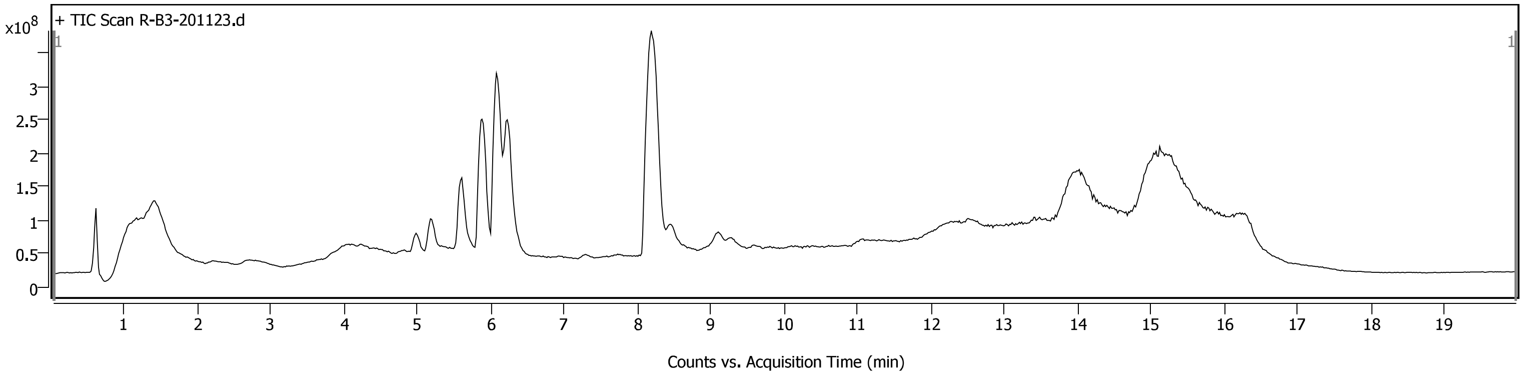

Compound Summary

Cpd	Name	Formula	CAS	RT	Mass	Mass (Tgt)	Diff (Tgt, ppm)	Score	Algorithm
1	Kaempferol	C15 H10 O6		5.806	286.0474	286.0477	-1.18	98.82	FBF
2		C15 H10 O6		5.806	286.0474	286.0477	-1.18	98.82	FBF
3	Galangin	C15 H10 O5		6.640	270.0472	270.0528	-20.90	47.61	FBF
4	Isorhamnetin	C16 H12 O7		5.708	316.0580	316.0583	-0.86	99.53	FBF
5	Chrysin	C15 H10 O4		1.015	254.0598	254.0579	7.30	67.40	FBF
6		C9 H8 O2		2.715	148.0520	148.0524	-2.80	89.65	FBF
7	Gallic acid	C7 H6 O5		1.849	170.0211	170.0215	-2.74	46.54	FBF
8		C7 H7 N O2		1.604	137.0474	137.0477	-1.79	80.08	FBF
9	Chorogenic acid	C16 H18 O9		1.358	354.0958	354.0951	2.12	53.02	FBF
10	(+)catechin	C15 H14 O6		5.675	290.0749	290.0790	-14.25	44.05	FBF
11	Naringenin	C15 H12 O5		1.031	272.0624	272.0685	-22.42	48.81	FBF
12	Rutin	C27 H30 O16		5.724	610.1520	610.1534	-2.22	95.88	FBF
13		C9 H10 O4		3.582	182.0583	182.0579	2.11	66.03	FBF
14	Sinapic acid	C11 H12 O5		5.152	224.0675	224.0685	-4.29	76.45	FBF
15		C16 H12 O7		5.708	316.0580	316.0583	-0.86	99.53	FBF
16		C15 H10 O4		1.015	254.0598	254.0579	7.30	67.40	FBF
17	Pincocarbin	C15 H12 O4		8.798	256.0798	256.0736	24.29	49.17	FBF
18	Caffeic acid	C9 H8 O4		4.792	180.0424	180.0423	0.95	85.06	FBF
19		C16 H18 O9		1.358	354.0958	354.0951	2.12	53.02	FBF
20	Vanallic acid	C8 H8 O4		1.277	168.0422	168.0423	-0.44	69.95	FBF
21	Benzoic acid	C7 H5 O2		0.623	121.0323	121.0290	27.25	11.97	FBF
22	Elagic acid	C14 H6 O8		0.623	302.0037	302.0063	-8.53	42.56	FBF
23	Syringic acid	C9 H10 O5		5.806	198.0522	198.0528	-3.10	73.45	FBF
24	Ferulic acid	C10 H10 O4		5.299	194.0595	194.0579	8.29	75.40	FBF
25	3,4-dihydroxybenzoic acid	C7 H6 O4		3.549	154.0256	154.0266	-6.63	79.18	FBF
26	Hesperdin	C16 H14 O6		0.623	302.0819	302.0790	9.39	67.76	FBF
27	Apigenin	C15 H10 O5		6.640	270.0472	270.0528	-20.90	47.61	FBF
28	epicatchin	C15 H14 O6		5.675	290.0749	290.0790	-14.25	44.05	FBF
29		C15 H10 O6		5.806	286.0474	286.0477	-1.18	98.82	FBF
30	Quercetin	C15 H10 O7		5.593	302.0424	302.0427	-0.94	85.20	FBF

Compound Details

Cpd. 1: C15 H10 O6

Name	Formula	RT	RI	Mass	Diff (Tgt, ppm)	CAS	ID Source	Score	Algorithm
	C15 H10 O6	5.806		286.0474	-1.18		FBF	98.82	FBF

Species	m/z	Score (Tgt)	Score (Lib)	Score (DB)	Score (MFG)	Score (RT)
(M+H)+ (M+NH4)+	287.0547 304.0765	98.82				

Compound Chromatograms (overlaid)

Structure

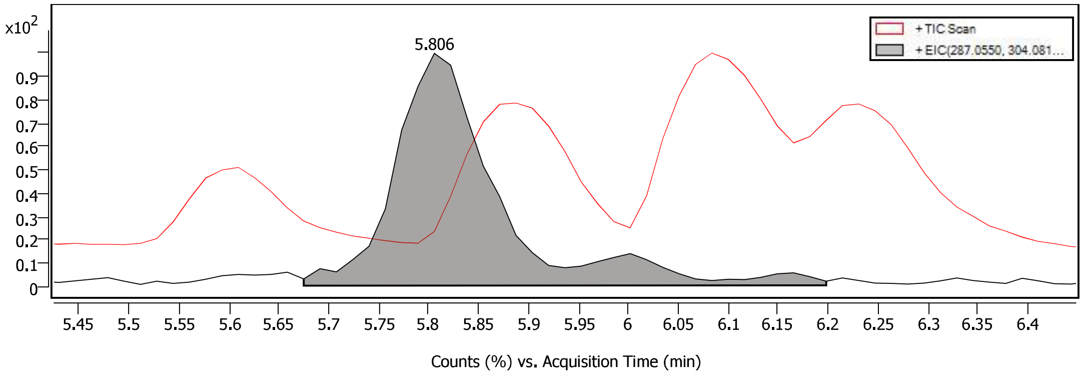

Compound Spectra (overlaid)

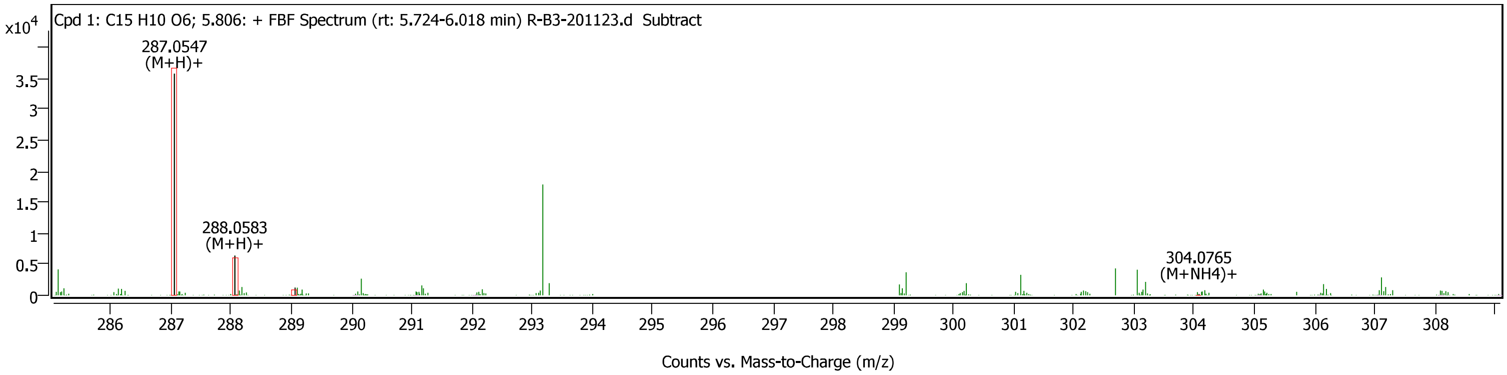

Compound ID Table

Name	Formula	Species	RT	RT Diff	Mass	CAS	ID Source	Score	Score (Lib)	Score (Tgt)
	C15 H10 O6	(M+H)+ (M+NH4)+	5.806		286.0474		FBF	98.82		98.82

Cpd. 2: C15 H10 O6

Name	Formula	RT	RI	Mass	Diff (Tgt, ppm)	CAS	ID Source	Score	Algorithm
	C15 H10 O6	5.806		286.0474	-1.18		FBF	98.82	FBF

Species	m/z	Score (Tgt)	Score (Lib)	Score (DB)	Score (MFG)	Score (RT)
(M+H)+ (M+NH4)+	287.0547 304.0765	98.82				

Compound Chromatograms (overlaid)

Structure

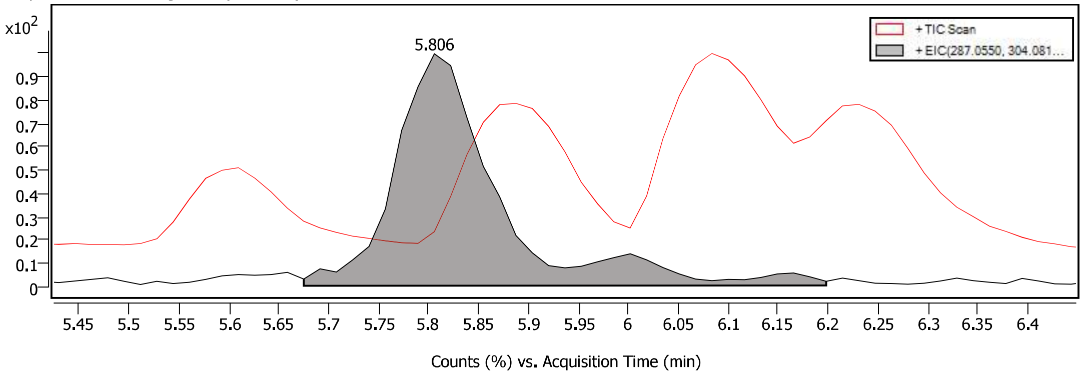

Compound Spectra (overlaid)

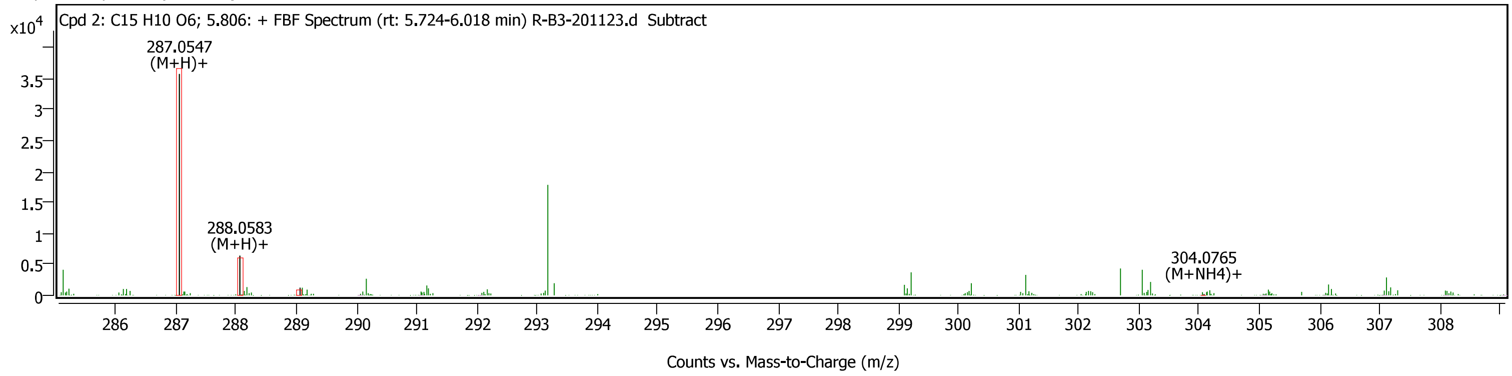

Compound ID Table

Name	Formula	Species	RT	RT Diff	Mass	CAS	ID Source	Score	Score (Lib)	Score (Tgt)
	C15 H10 O6	(M+H)+ (M+NH4)+	5.806		286.0474		FBF	98.82		98.82

Cpd. 3: C15 H10 O5

Name	Formula	RT	RI	Mass	Diff (Tgt, ppm)	CAS	ID Source	Score	Algorithm
	C15 H10 O5	6.640		270.0472	-20.90		FBF	47.61	FBF

Species	m/z	Score (Tgt)	Score (Lib)	Score (DB)	Score (MFG)	Score (RT)
(M+H)+ (M+NH4)+ (M+Na)+	271.0532 288.0862 293.0421	47.61				

Compound Chromatograms (overlaid)

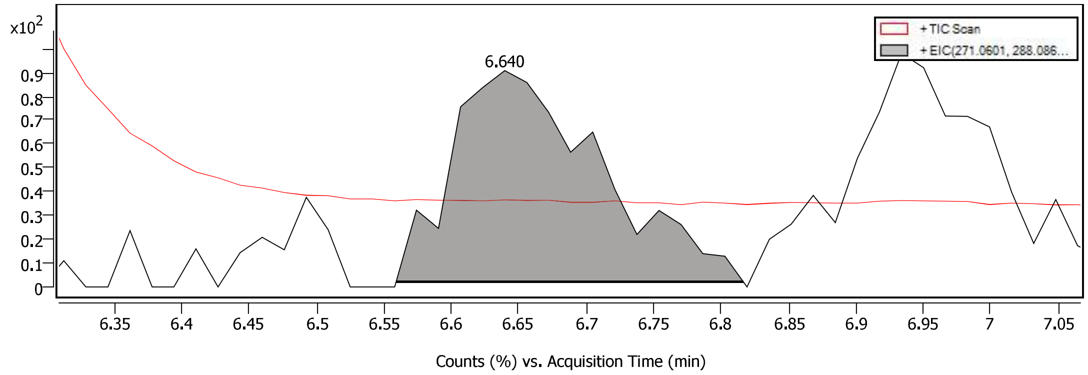

Structure

Compound Spectra (overlaid)

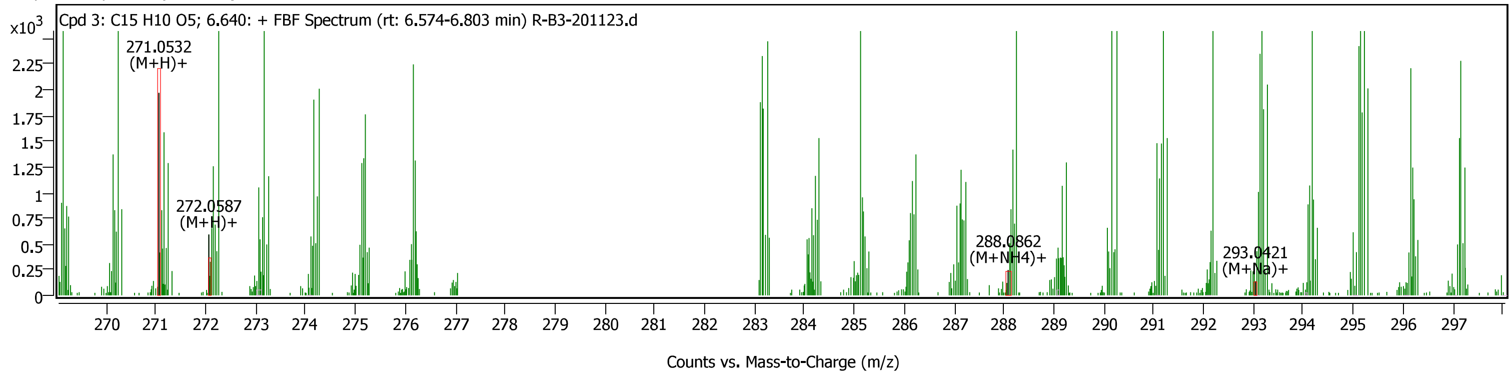

Compound ID Table

Name	Formula	Species	RT	RT Diff	Mass	CAS	ID Source	Score	Score (Lib)	Score (Tgt)
	C15 H10 O5	(M+H)+ (M+NH4)+ (M+Na)+	6.640		270.0472		FBF	47.61		47.61

Cpd. 4: C16 H12 O7

Name	Formula	RT	RI	Mass	Diff (Tgt, ppm)	CAS	ID Source	Score	Algorithm
	C16 H12 O7	5.708		316.0580	-0.86		FBF	99.53	FBF

Species	m/z	Score (Tgt)	Score (Lib)	Score (DB)	Score (MFG)	Score (RT)
(M+H)+ (M+NH4)+ (M+Na)+	317.0653 334.0900 339.0561	99.53				

Compound Chromatograms (overlaid)

Structure

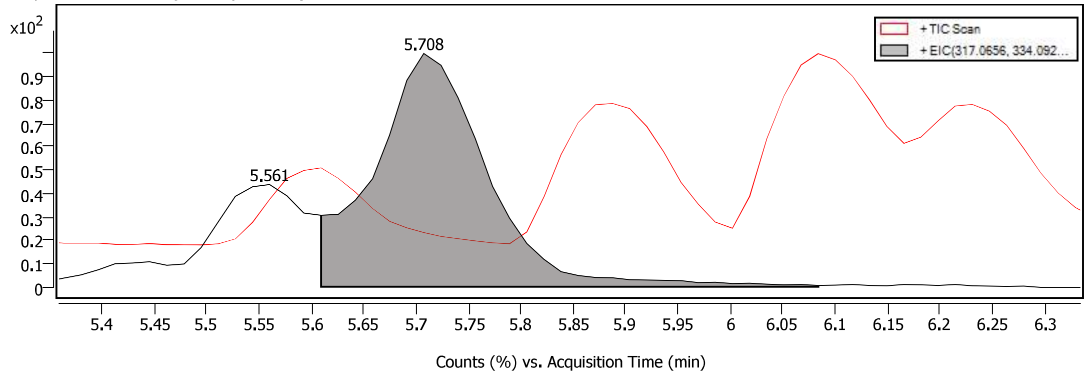

Compound Spectra (overlaid)

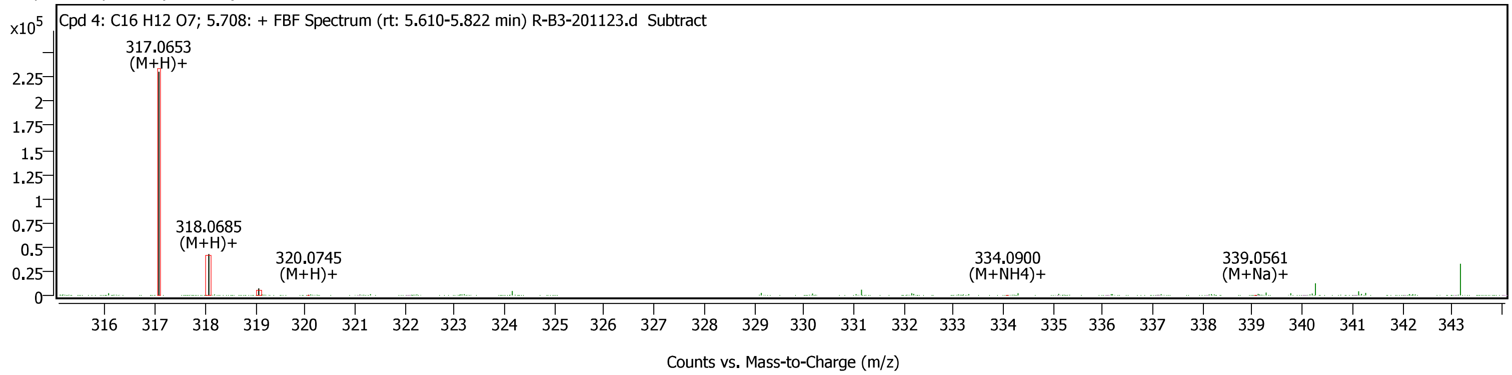

Compound ID Table

Name	Formula	Species	RT	RT Diff	Mass	CAS	ID Source	Score	Score (Lib)	Score (Tgt)
	C16 H12 O7	(M+H)+<br>(M+NH4)+<br>(M+Na)+	5.708		316.0580		FBF	99.53		99.53

Cpd. 5: C15 H10 O4

Name	Formula	RT	RI	Mass	Diff (Tgt, ppm)	CAS	ID Source	Score	Algorithm
	C15 H10 O4	1.015		254.0598	7.30		FBF	67.40	FBF

Species	m/z	Score (Tgt)	Score (Lib)	Score (DB)	Score (MFG)	Score (RT)
(M+Na)+	277.0494	67.40				

Compound Chromatograms (overlaid)

Structure

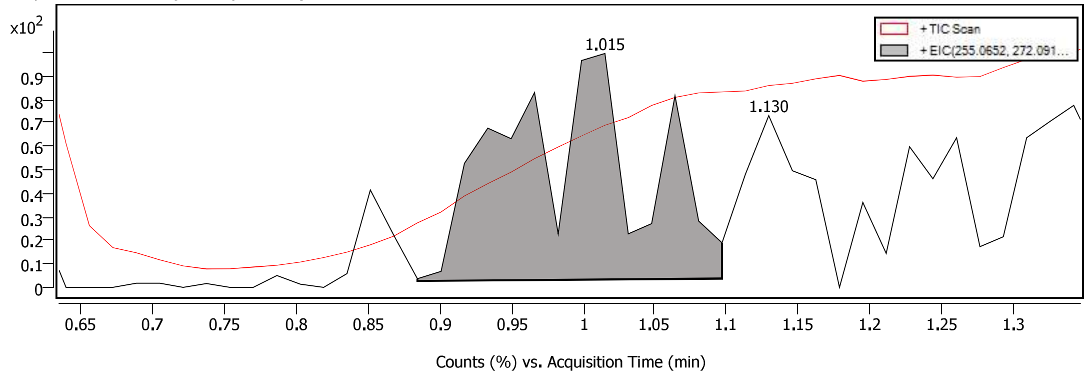

Compound Spectra (overlaid)

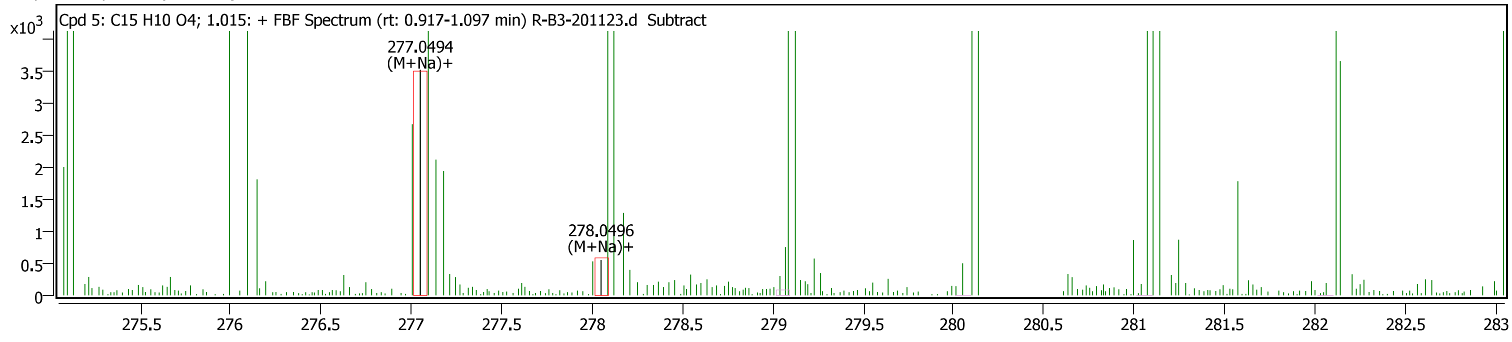

Compound ID Table

Name	Formula	Species	RT	RT Diff	Mass	CAS	ID Source	Score	Score (Lib)	Score (Tgt)
	C15 H10 O4	(M+Na)+	1.015		254.0598		FBF	67.40		67.40

Cpd. 6: C9 H8 O2

Name	Formula	RT	RI	Mass	Diff (Tgt, ppm)	CAS	ID Source	Score	Algorithm
	C9 H8 O2	2.715		148.0520	-2.80		FBF	89.65	FBF

Species	m/z	Score (Tgt)	Score (Lib)	Score (DB)	Score (MFG)	Score (RT)
(M+H)+ (M+NH4)+ (M+Na)+	149.0594 166.0860 171.0440	89.65				

Compound Chromatograms (overlaid)

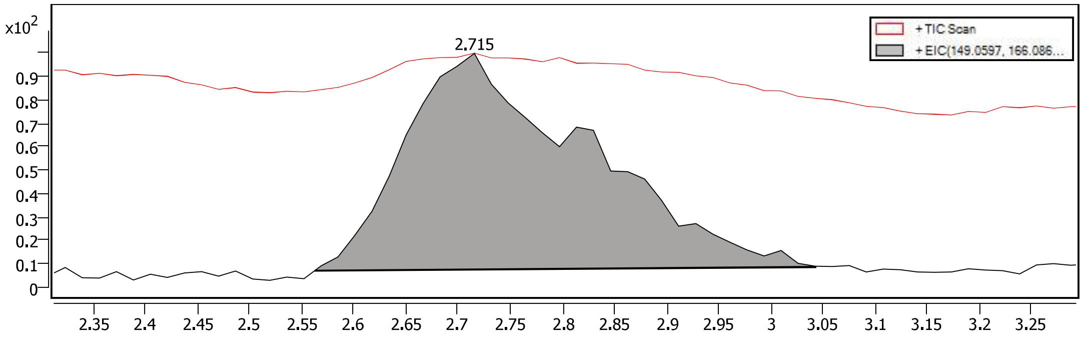

Structure

Compound Spectra (overlaid)

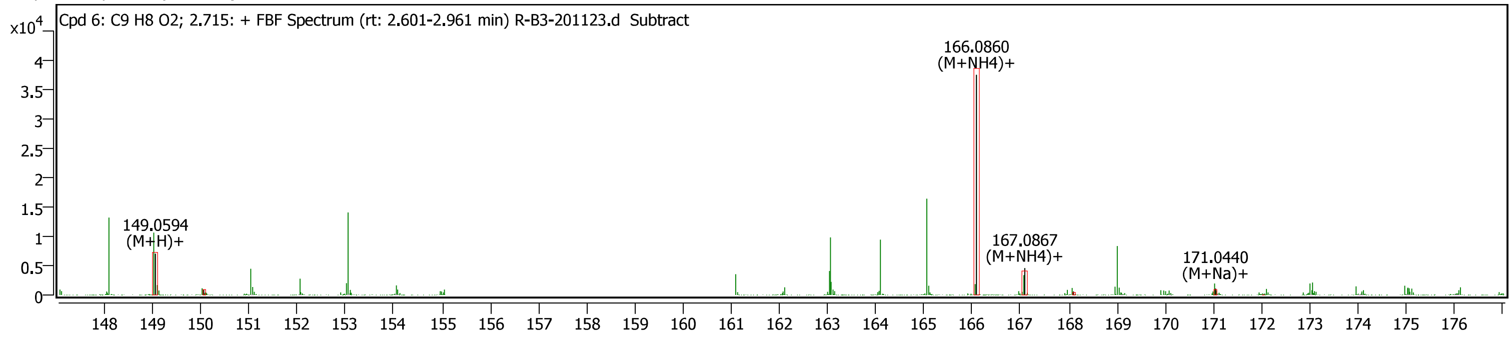

Compound ID Table

Name	Formula	Species	RT	RT Diff	Mass	CAS	ID Source	Score	Score (Lib)	Score (Tgt)
	C9 H8 O2	(M+H)+ (M+NH4)+ (M+Na)+	2.715		148.0520		FBF	89.65		89.65

Cpd. 7: C7 H6 O5

Name	Formula	RT	RI	Mass	Diff (Tgt, ppm)	CAS	ID Source	Score	Algorithm
	C7 H6 O5	1.849		170.0211	-2.74		FBF	46.54	FBF

Species	m/z	Score (Tgt)	Score (Lib)	Score (DB)	Score (MFG)	Score (RT)
(M+Na)+	193.0103	46.54				

Target Screening Report

Compound Chromatograms (overlaid)

Structure

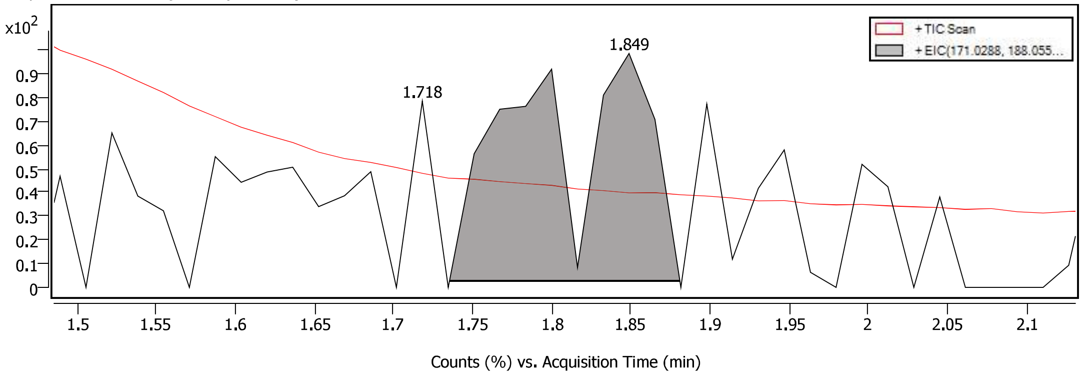

Compound Spectra (overlaid)

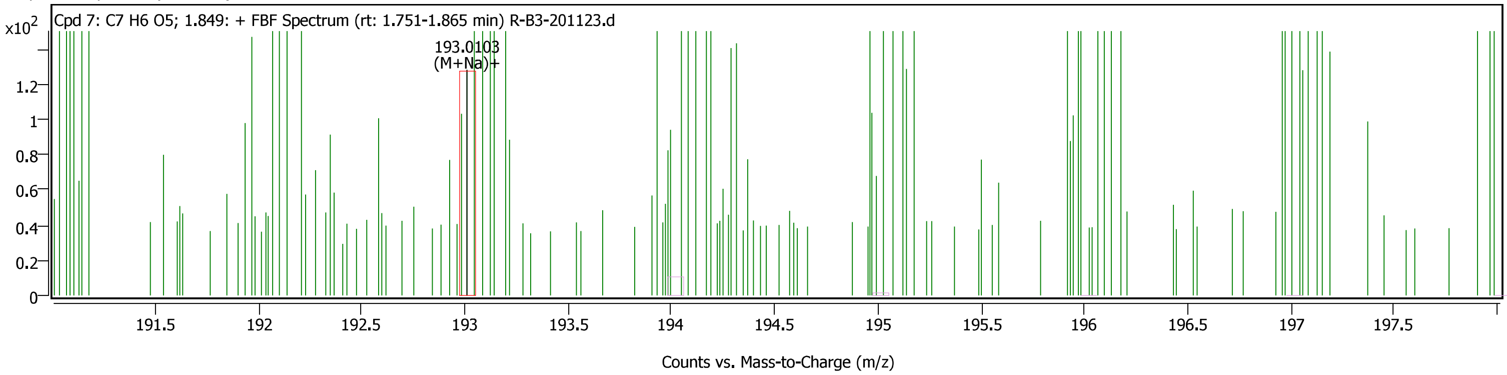

Compound ID Table

Name	Formula	Species	RT	RT Diff	Mass	CAS	ID Source	Score	Score (Lib)	Score (Tgt)
	C7 H6 O5	(M+Na)+	1.849		170.0211		FBF	46.54		46.54

Cpd. 8: C7 H7 N O2

Name	Formula	RT	RI	Mass	Diff (Tgt, ppm)	CAS	ID Source	Score	Algorithm
	C7 H7 N O2	1.604		137.0474	-1.79		FBF	80.08	FBF

Species	m/z	Score (Tgt)	Score (Lib)	Score (DB)	Score (MFG)	Score (RT)
(M+H)+ (M+NH4)+ (M+Na)+	138.0554 155.0781 160.0398	80.08				

Compound Chromatograms (overlaid)

Structure

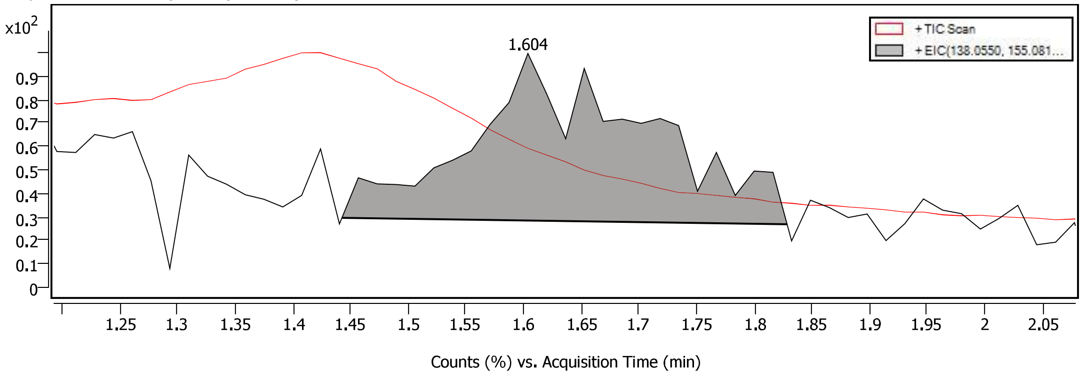

Compound Spectra (overlaid)

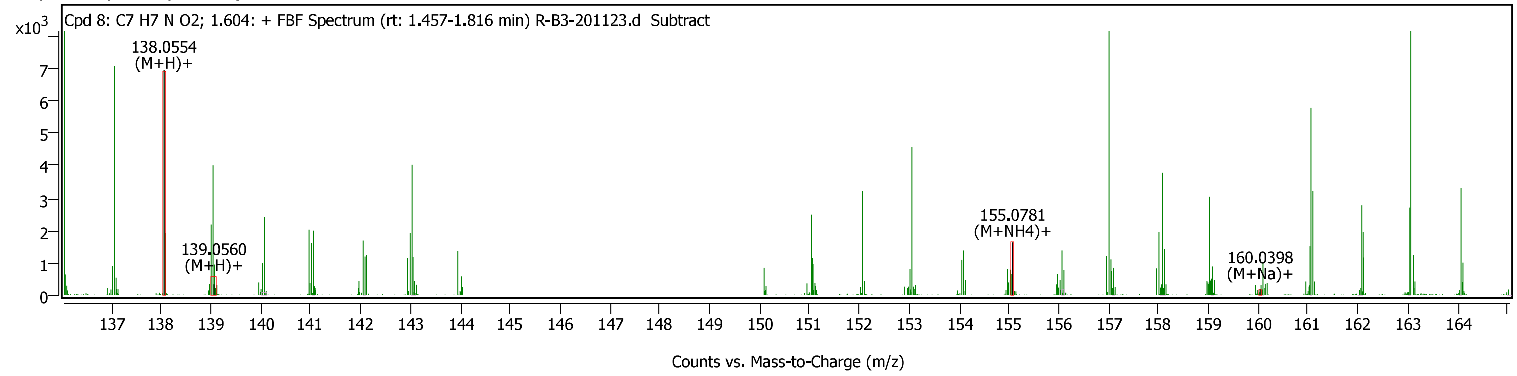

Compound ID Table

Name	Formula	Species	RT	RT Diff	Mass	CAS	ID Source	Score	Score (Lib)	Score (Tgt)
	C7 H7 N O2	(M+H)+ (M+NH4)+ (M+Na)+	1.604		137.0474		FBF	80.08		80.08

Cpd. 9: C16 H18 O9

Name	Formula	RT	RI	Mass	Diff (Tgt, ppm)	CAS	ID Source	Score	Algorithm
	C16 H18 O9	1.358		354.0958	2.12		FBF	53.02	FBF

Species	m/z	Score (Tgt)	Score (Lib)	Score (DB)	Score (MFG)	Score (RT)
(M+H)+	355.1051	53.02				

Compound Chromatograms (overlaid)

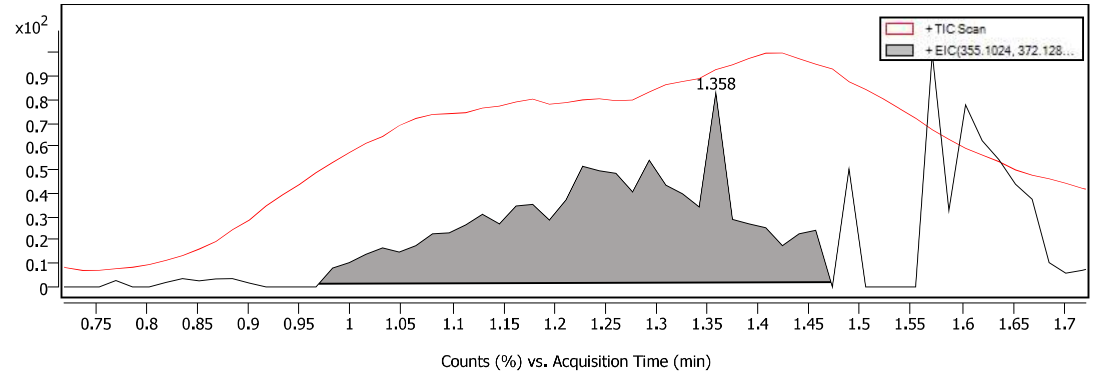

Structure

Compound Spectra (overlaid)

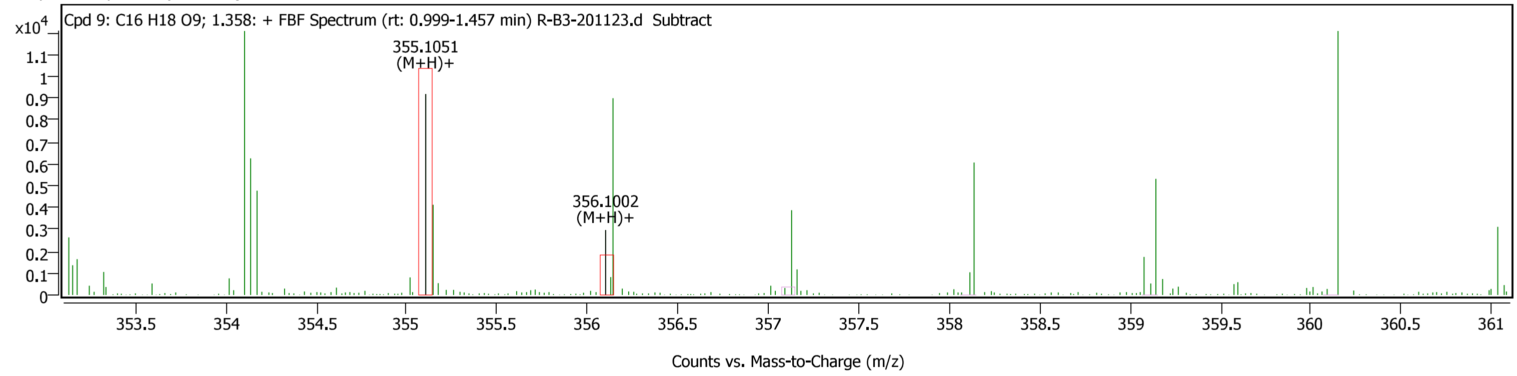

Compound ID Table

Name	Formula	Species	RT	RT Diff	Mass	CAS	ID Source	Score	Score (Lib)	Score (Tgt)
	C16 H18 O9	(M+H)+	1.358		354.0958		FBF	53.02		53.02

Cpd. 10: C15 H14 O6

Name	Formula	RT	RI	Mass	Diff (Tgt, ppm)	CAS	ID Source	Score	Algorithm
	C15 H14 O6	5.675		290.0749	-14.25		FBF	44.05	FBF

Species	m/z	Score (Tgt)	Score (Lib)	Score (DB)	Score (MFG)	Score (RT)
(M+H)+ (M+NH4)+ (M+Na)+	291.0806 308.1118 313.0593	44.05				

Compound Chromatograms (overlaid)

Structure

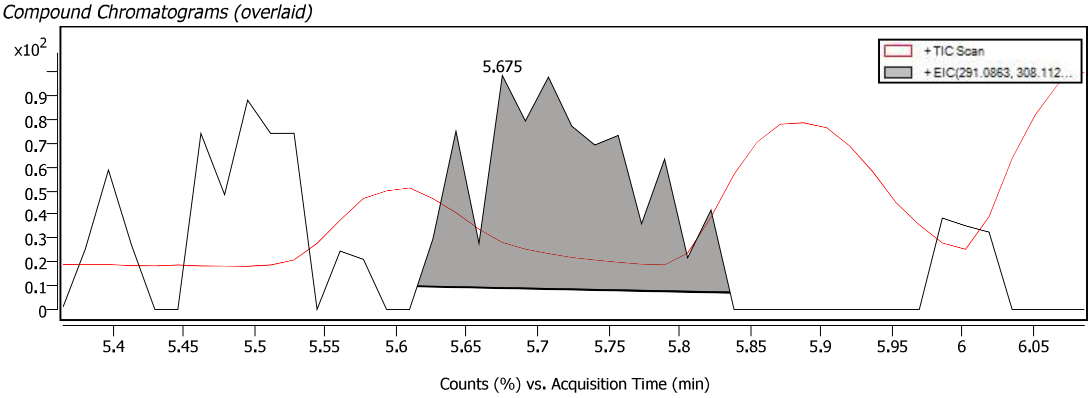

Compound Spectra (overlaid)

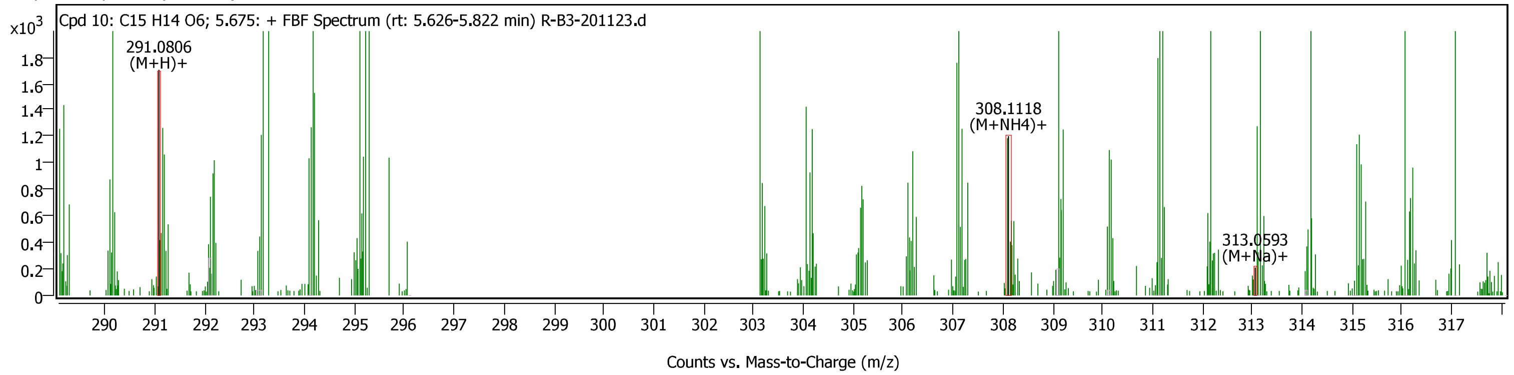

Compound ID Table

Name	Formula	Species	RT	RT Diff	Mass	CAS	ID Source	Score	Score (Lib)	Score (Tgt)
	C15 H14 O6	(M+H)+ (M+NH4)+ (M+Na)+	5.675		290.0749		FBF	44.05		44.05

Cpd. 11: C15 H12 O5

Name	Formula	RT	RI	Mass	Diff (Tgt, ppm)	CAS	ID Source	Score	Algorithm
	C15 H12 O5	1.031		272.0624	-22.42		FBF	48.81	FBF

Species	m/z	Score (Tgt)	Score (Lib)	Score (DB)	Score (MFG)	Score (RT)
(M+H)+ (M+NH4)+ (M+Na)+	273.0774 290.0952 295.0614	48.81				

Compound Chromatograms (overlaid)

Structure

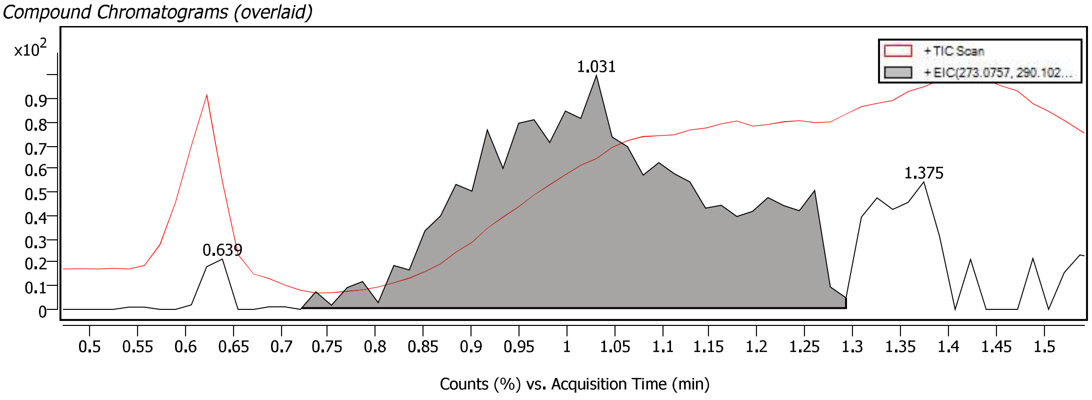

Compound Spectra (overlaid)

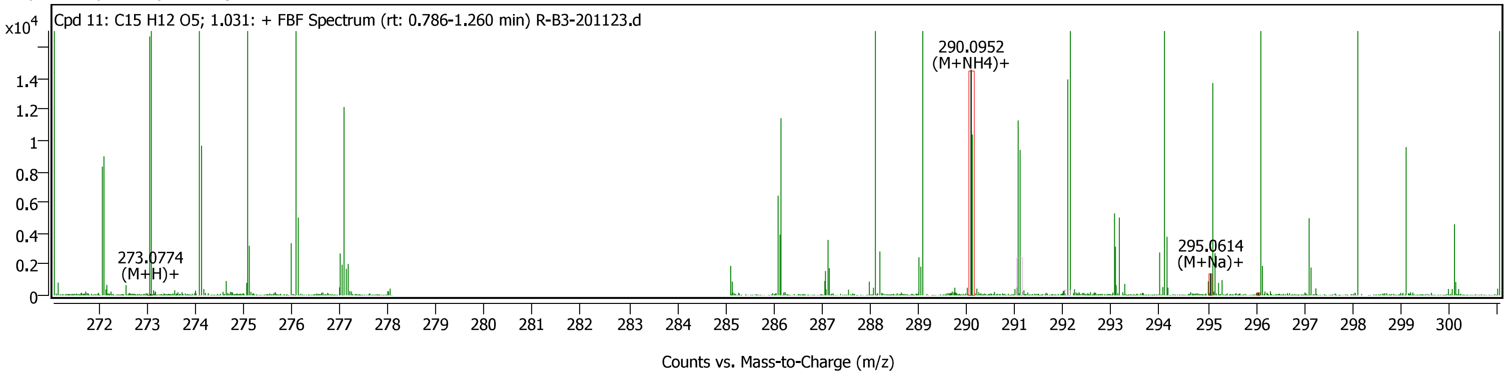

Compound ID Table

Name	Formula	Species	RT	RT Diff	Mass	CAS	ID Source	Score	Score (Lib)	Score (Tgt)
	C15 H12 O5	(M+H)+ (M+NH4)+ (M+Na)+	1.031		272.0624		FBF	48.81		48.81

Cpd. 12: C27 H30 O16

Name	Formula	RT	RI	Mass	Diff (Tgt, ppm)	CAS	ID Source	Score	Algorithm
	C27 H30 O16	5.724		610.1520	-2.22		FBF	95.88	FBF

Species	m/z	Score (Tgt)	Score (Lib)	Score (DB)	Score (MFG)	Score (RT)
(M+H)+ (M+NH4)+ (M+Na)+	611.1603 628.1819 633.1398	95.88				

Compound Chromatograms (overlaid)

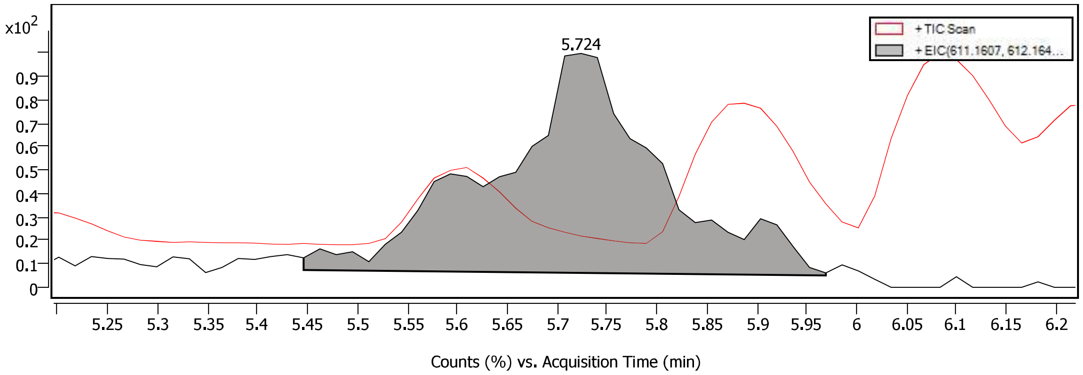

Structure

Compound Spectra (overlaid)

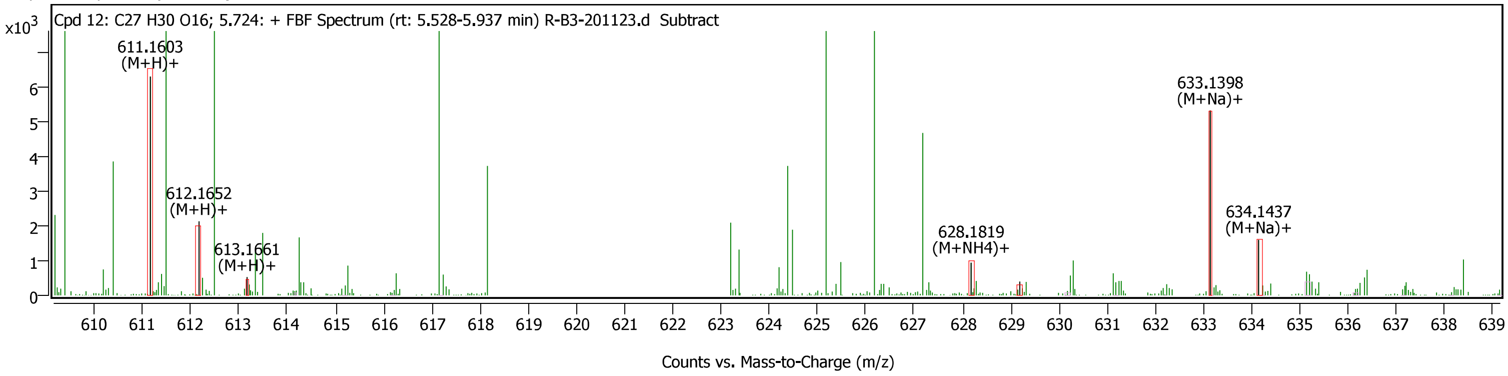

Compound ID Table

Name	Formula	Species	RT	RT Diff	Mass	CAS	ID Source	Score	Score (Lib)	Score (Tgt)
	C27 H30 O16	(M+H)+ (M+NH4)+ (M+Na)+	5.724		610.1520		FBF	95.88		95.88

Cpd. 13: C9 H10 O4

Name	Formula	RT	RI	Mass	Diff (Tgt, ppm)	CAS	ID Source	Score	Algorithm
	C9 H10 O4	3.582		182.0583	2.11		FBF	66.03	FBF

Species	m/z	Score (Tgt)	Score (Lib)	Score (DB)	Score (MFG)	Score (RT)
(M+H)+ (M+NH4)+ (M+Na)+	183.0661 200.0919 205.0482	66.03				

Compound Chromatograms (overlaid)

Structure

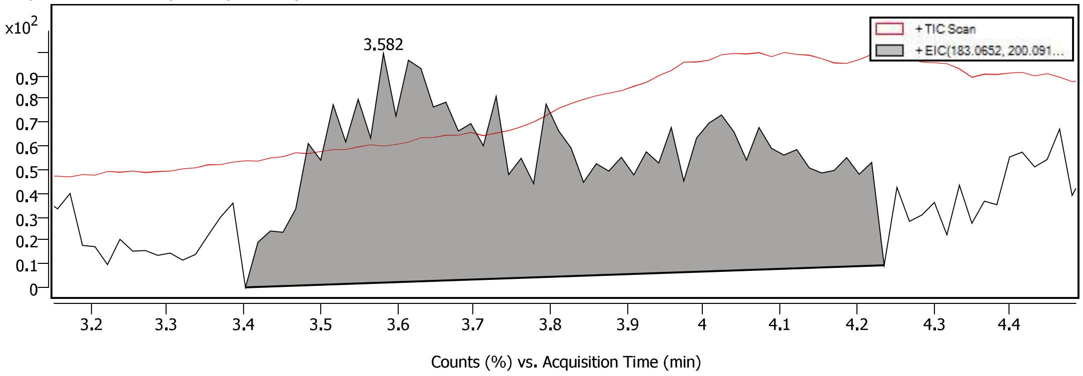

Compound Spectra (overlaid)

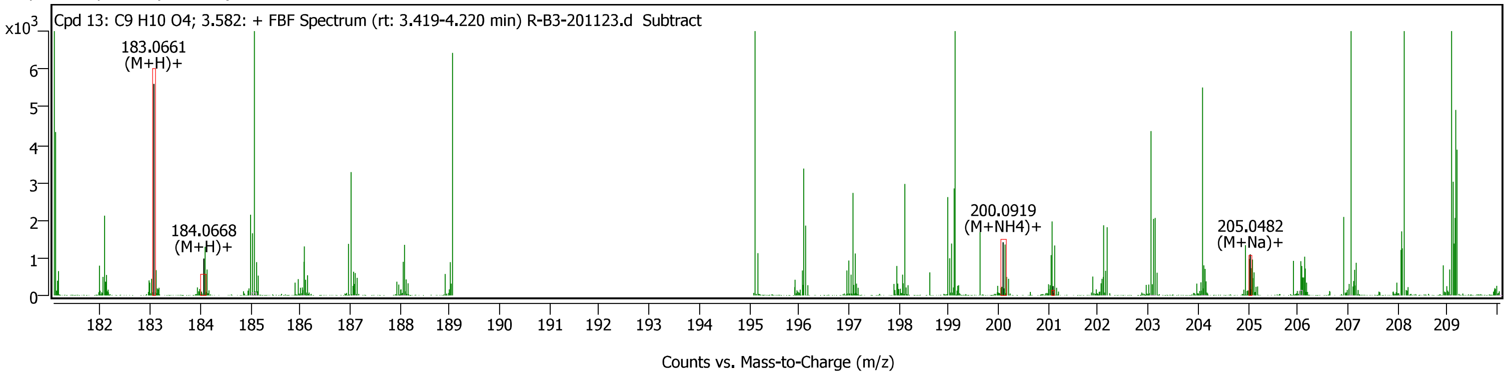

Compound ID Table

Name	Formula	Species	RT	RT Diff	Mass	CAS	ID Source	Score	Score (Lib)	Score (Tgt)
	C9 H10 O4	(M+H)+ (M+NH4)+ (M+Na)+	3.582		182.0583		FBF	66.03		66.03

Cpd. 14: C11 H12 O5

Name	Formula	RT	RI	Mass	Diff (Tgt, ppm)	CAS	ID Source	Score	Algorithm
	C11 H12 O5	5.152		224.0675	-4.29		FBF	76.45	FBF

Species	m/z	Score (Tgt)	Score (Lib)	Score (DB)	Score (MFG)	Score (RT)
(M+H)+ (M+NH4)+ (M+Na)+	225.0750 242.1006 247.0588	76.45				

Compound Chromatograms (overlaid)

Structure

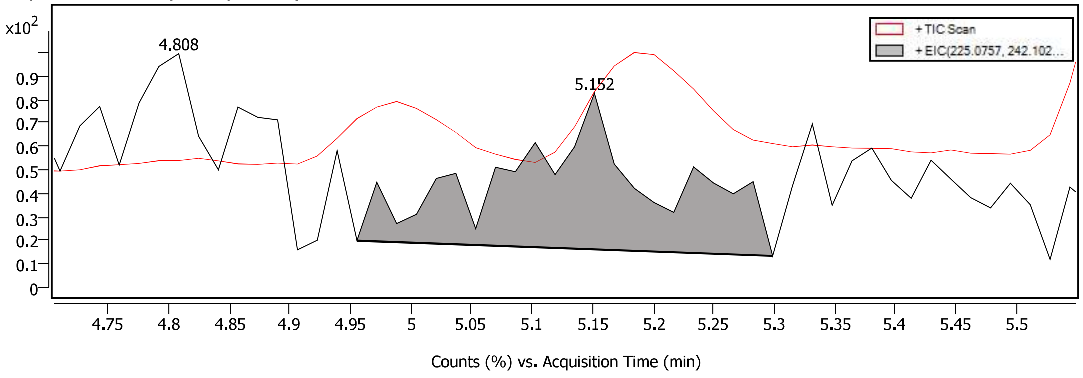

Compound Spectra (overlaid)

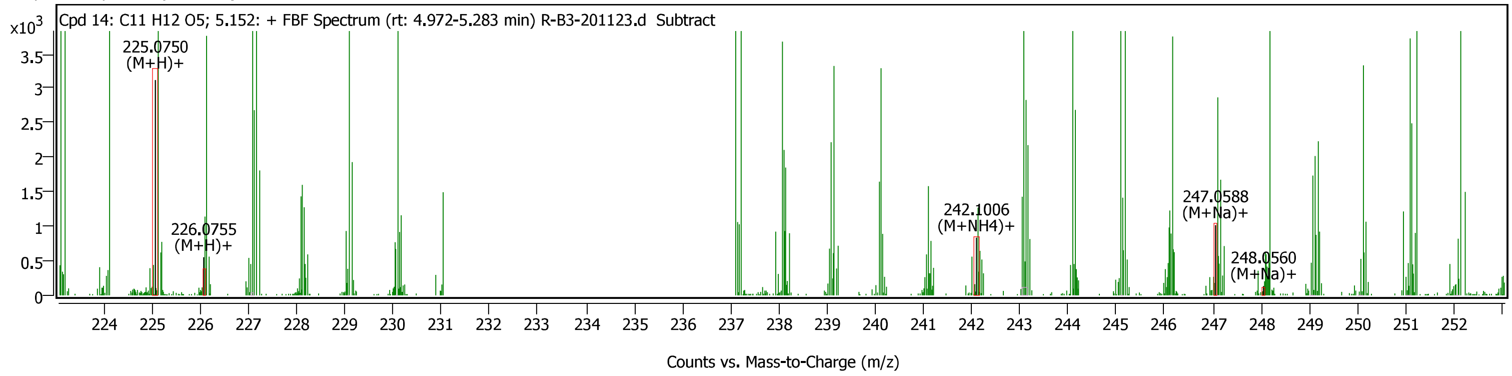

Compound ID Table

Name	Formula	Species	RT	RT Diff	Mass	CAS	ID Source	Score	Score (Lib)	Score (Tgt)
	C11 H12 O5	(M+H)+ (M+NH4)+ (M+Na)+	5.152		224.0675		FBF	76.45		76.45

Cpd. 15: C16 H12 O7

Name	Formula	RT	RI	Mass	Diff (Tgt, ppm)	CAS	ID Source	Score	Algorithm
	C16 H12 O7	5.708		316.0580	-0.86		FBF	99.53	FBF

Species	m/z	Score (Tgt)	Score (Lib)	Score (DB)	Score (MFG)	Score (RT)
(M+H)+ (M+NH4)+ (M+Na)+	317.0653 334.0900 339.0561	99.53				

Compound Chromatograms (overlaid)

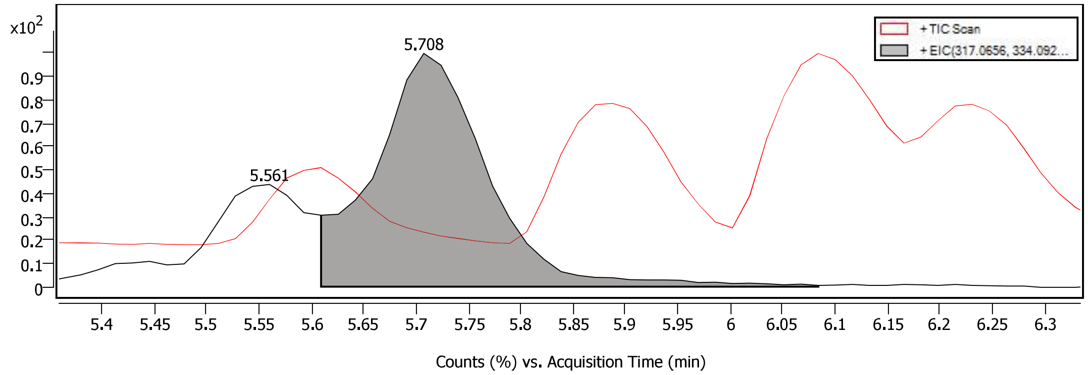

Structure

Compound Spectra (overlaid)

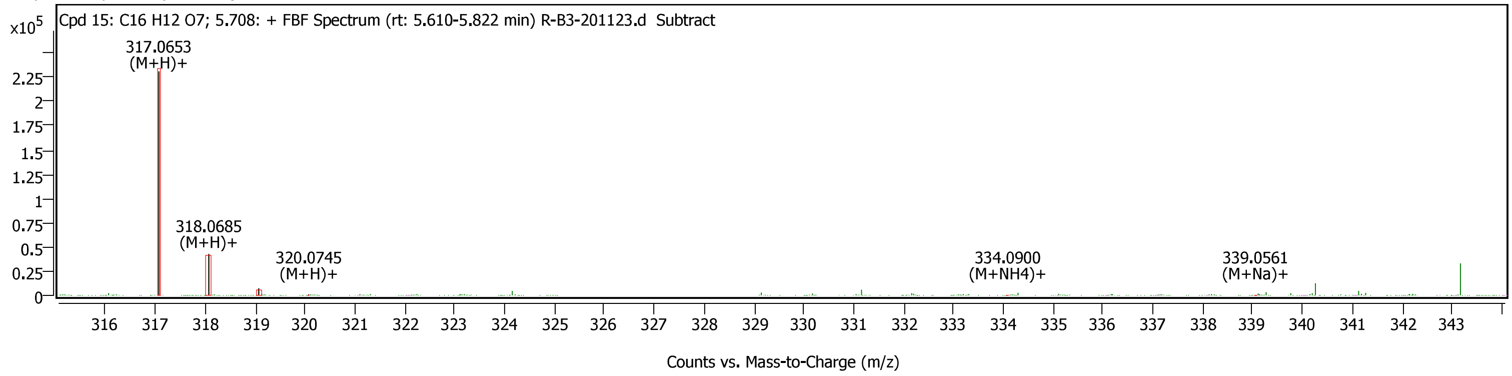

Compound ID Table

Name	Formula	Species	RT	RT Diff	Mass	CAS	ID Source	Score	Score (Lib)	Score (Tgt)
	C16 H12 O7	(M+H)+ (M+NH4)+ (M+Na)+	5.708		316.0580		FBF	99.53		99.53

Cpd. 16: C15 H10 O4

Name	Formula	RT	RI	Mass	Diff (Tgt, ppm)	CAS	ID Source	Score	Algorithm
	C15 H10 O4	1.015		254.0598	7.30		FBF	67.40	FBF

Species	m/z	Score (Tgt)	Score (Lib)	Score (DB)	Score (MFG)	Score (RT)
(M+Na)+	277.0494	67.40				

Compound Chromatograms (overlaid)

Structure

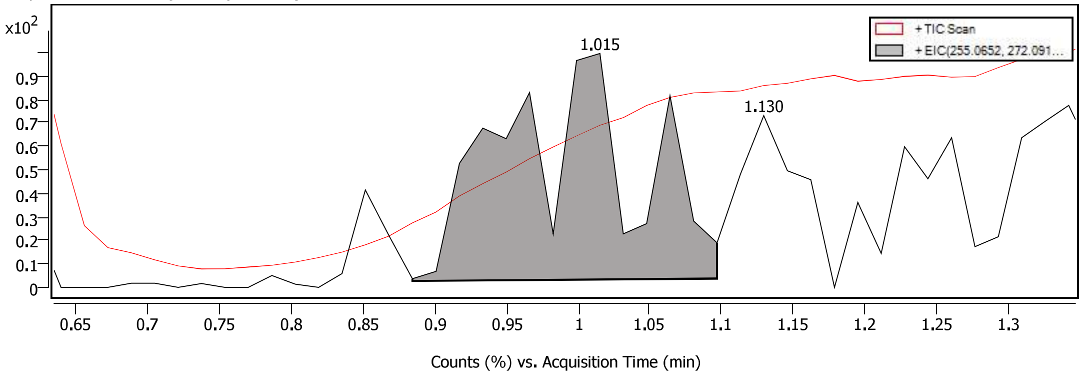

Compound Spectra (overlaid)

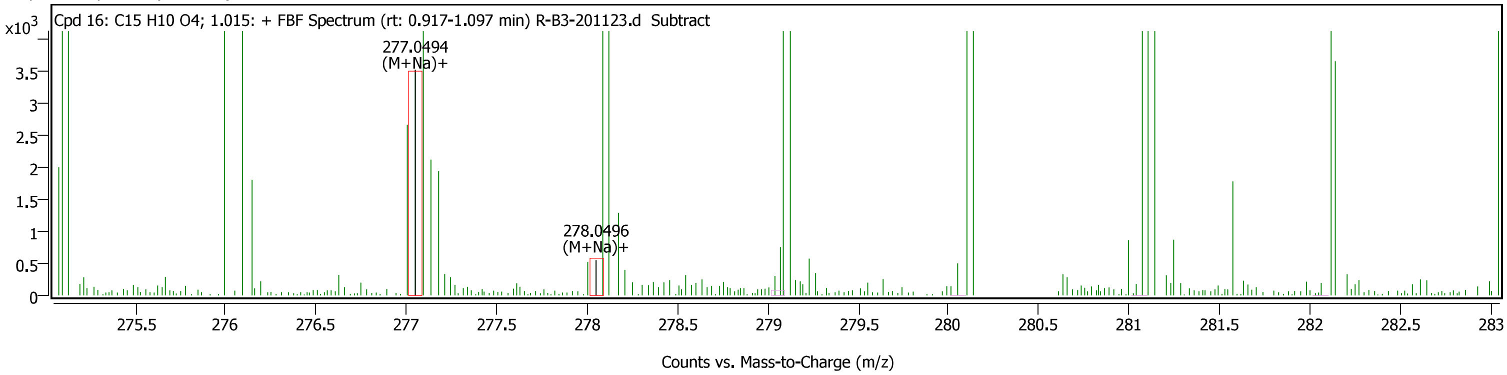

Compound ID Table

Name	Formula	Species	RT	RT Diff	Mass	CAS	ID Source	Score	Score (Lib)	Score (Tgt)
	C15 H10 O4	(M+Na)+	1.015		254.0598		FBF	67.40		67.40

Cpd. 17: C15 H12 O4

Name	Formula	RT	RI	Mass	Diff (Tgt, ppm)	CAS	ID Source	Score	Algorithm
	C15 H12 O4	8.798		256.0798	24.29		FBF	49.17	FBF

Species	m/z	Score (Tgt)	Score (Lib)	Score (DB)	Score (MFG)	Score (RT)
(M+H)+ (M+NH4)+ (M+Na)+	257.0855 274.1121 279.0693	49.17				

Compound Chromatograms (overlaid)

Structure

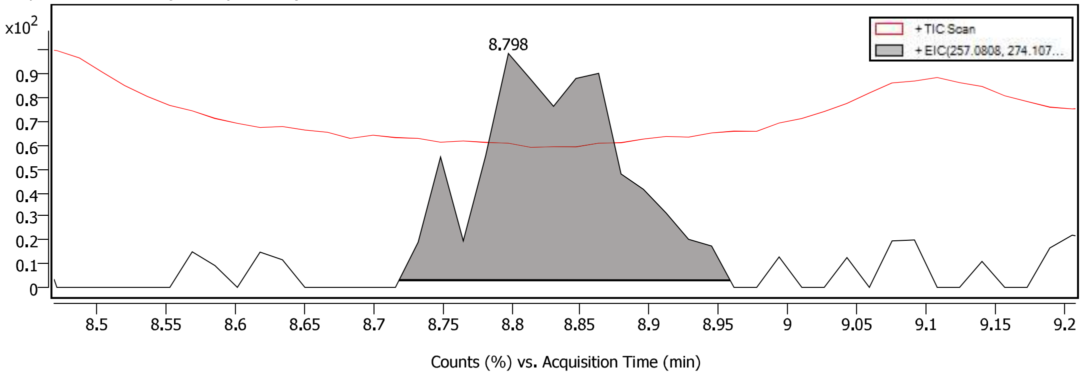

Compound Spectra (overlaid)

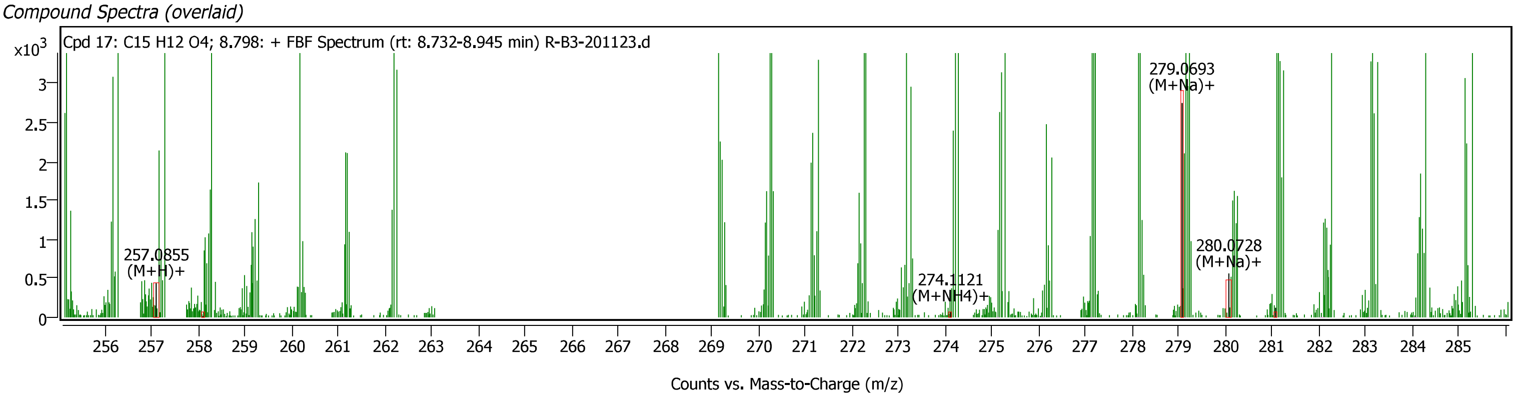

Compound ID Table

Name	Formula	Species	RT	RT Diff	Mass	CAS	ID Source	Score	Score (Lib)	Score (Tgt)
	C15 H12 O4	(M+H)+ (M+NH4)+ (M+Na)+	8.798		256.0798		FBF	49.17		49.17

Cpd. 18: C9 H8 O4

Name	Formula	RT	RI	Mass	Diff (Tgt, ppm)	CAS	ID Source	Score	Algorithm
	C9 H8 O4	4.792		180.0424	0.95		FBF	85.06	FBF

Species	m/z	Score (Tgt)	Score (Lib)	Score (DB)	Score (MFG)	Score (RT)
(M+H)+ (M+NH4)+ (M+Na)+	181.0497 198.0790 203.0278	85.06				

Compound Chromatograms (overlaid)

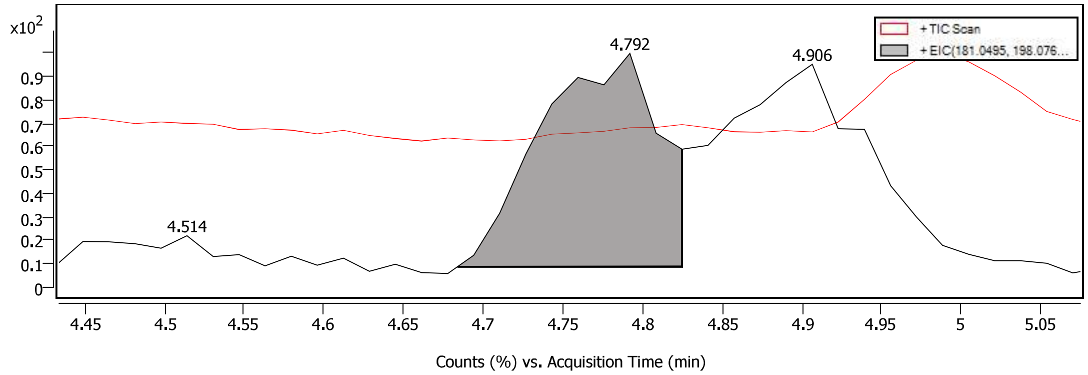

Structure

Compound Spectra (overlaid)

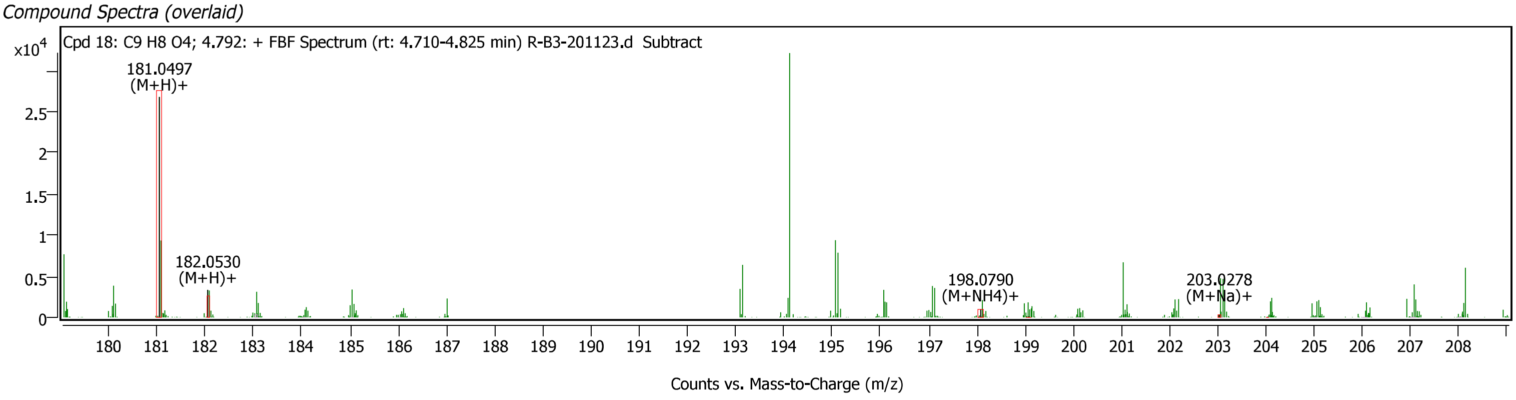

Compound ID Table

Name	Formula	Species	RT	RT Diff	Mass	CAS	ID Source	Score	Score (Lib)	Score (Tgt)
	C9 H8 O4	(M+H)+ (M+NH4)+ (M+Na)+	4.792		180.0424		FBF	85.06		85.06

Cpd. 19: C16 H18 O9

Name	Formula	RT	RI	Mass	Diff (Tgt, ppm)	CAS	ID Source	Score	Algorithm
	C16 H18 O9	1.358		354.0958	2.12		FBF	53.02	FBF

Species	m/z	Score (Tgt)	Score (Lib)	Score (DB)	Score (MFG)	Score (RT)
(M+H)+	355.1051	53.02				

Compound Chromatograms (overlaid)

Structure

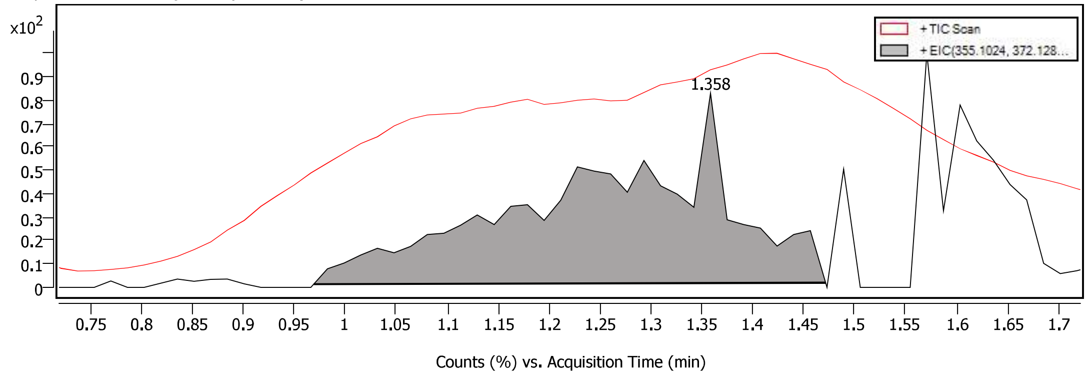

Compound Spectra (overlaid)

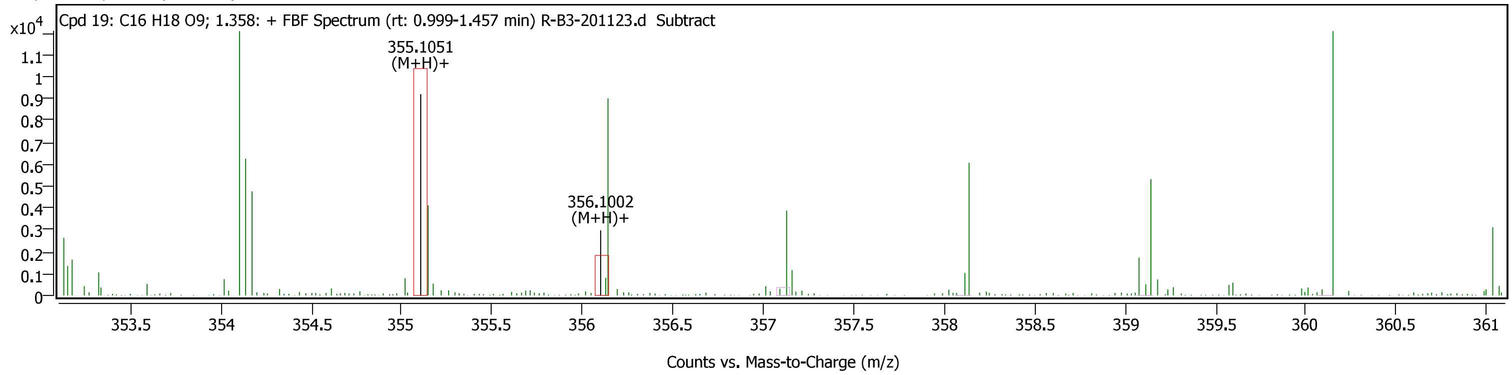

Compound ID Table

Name	Formula	Species	RT	RT Diff	Mass	CAS	ID Source	Score	Score (Lib)	Score (Tgt)
	C16 H18 O9	(M+H)+	1.358		354.0958		FBF	53.02		53.02

Cpd. 20: C8 H8 O4

Name	Formula	RT	RI	Mass	Diff (Tgt, ppm)	CAS	ID Source	Score	Algorithm
	C8 H8 O4	1.277		168.0422	-0.44		FBF	69.95	FBF

Species	m/z	Score (Tgt)	Score (Lib)	Score (DB)	Score (MFG)	Score (RT)
(M+H)+ (M+NH4)+	169.0494 186.0760	69.95				

Compound Chromatograms (overlaid)

Structure

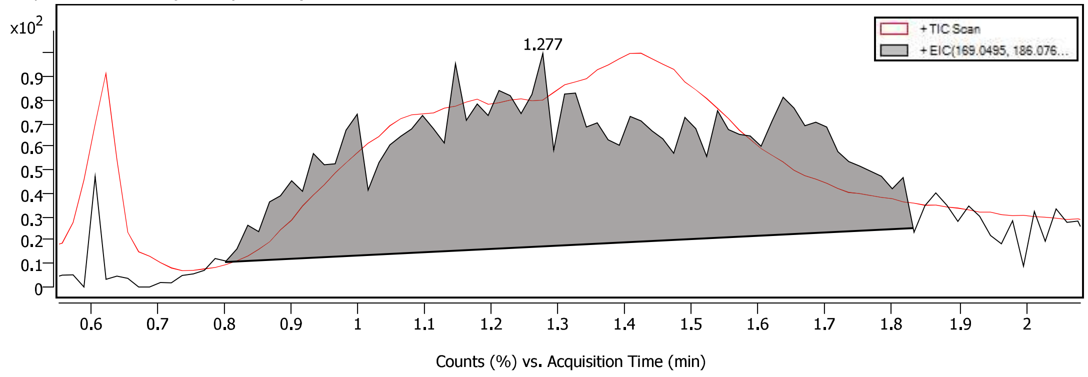

Compound Spectra (overlaid)

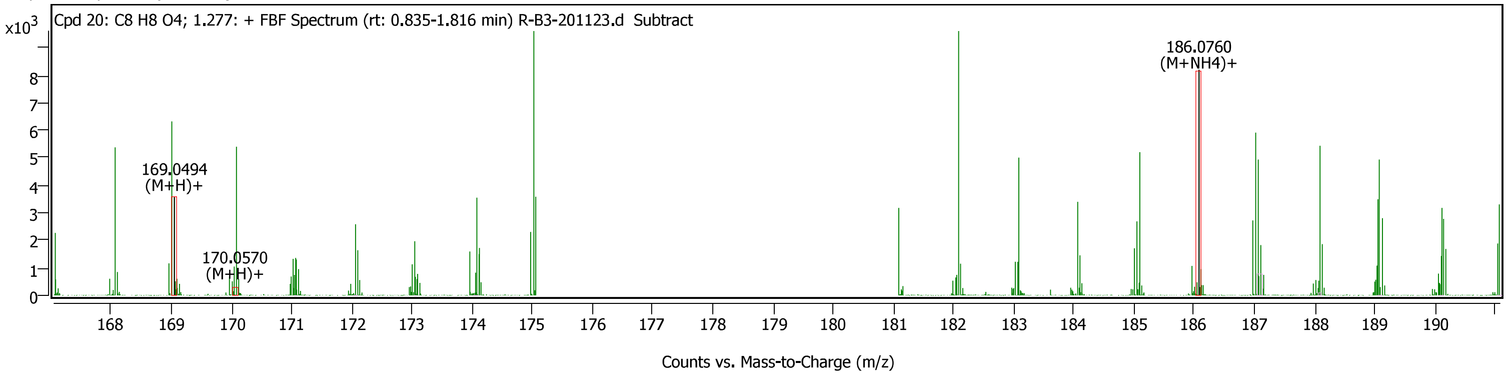

Compound ID Table

Name	Formula	Species	RT	RT Diff	Mass	CAS	ID Source	Score	Score (Lib)	Score (Tgt)
	C8 H8 O4	(M+H)+<br>(M+NH4)+	1.277		168.0422		FBF	69.95		69.95

Cpd. 21: C7 H5 O2

Name	Formula	RT	RI	Mass	Diff (Tgt, ppm)	CAS	ID Source	Score	Algorithm
	C7 H5 O2	0.623		121.0323	27.25		FBF	11.97	FBF

Species	m/z	Score (Tgt)	Score (Lib)	Score (DB)	Score (MFG)	Score (RT)
(M+Na)+	144.0215	11.97				

Compound Chromatograms (overlaid)

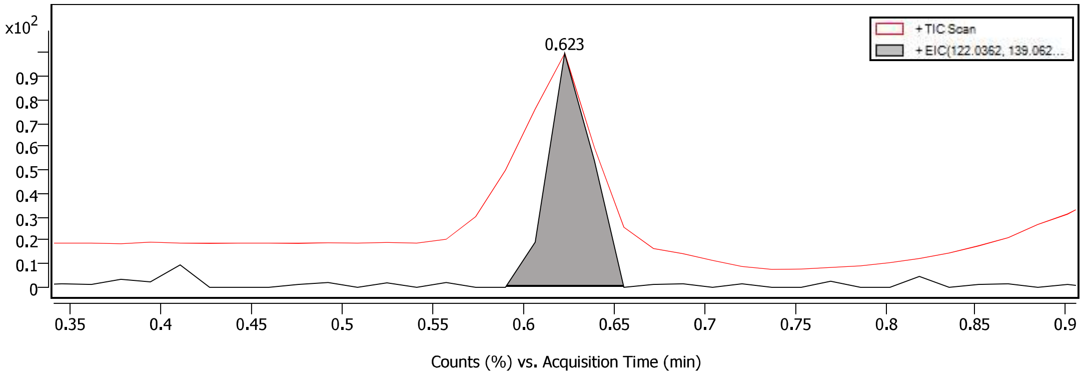

Structure

Compound Spectra (overlaid)

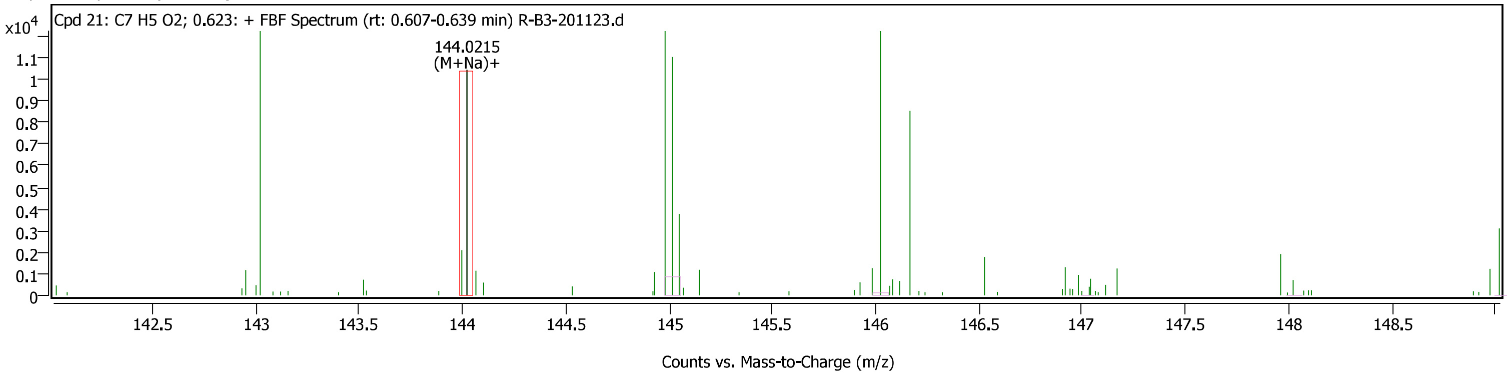

Compound ID Table

Name	Formula	Species	RT	RT Diff	Mass	CAS	ID Source	Score	Score (Lib)	Score (Tgt)
	C7 H5 O2	(M+Na)+	0.623		121.0323		FBF	11.97		11.97

Cpd. 22: C14 H6 O8

Name	Formula	RT	RI	Mass	Diff (Tgt, ppm)	CAS	ID Source	Score	Algorithm
	C14 H6 O8	0.623		302.0037	-8.53		FBF	42.56	FBF

Species	m/z	Score (Tgt)	Score (Lib)	Score (DB)	Score (MFG)	Score (RT)
(M+NH4)+	320.0374	42.56				

Compound Chromatograms (overlaid)

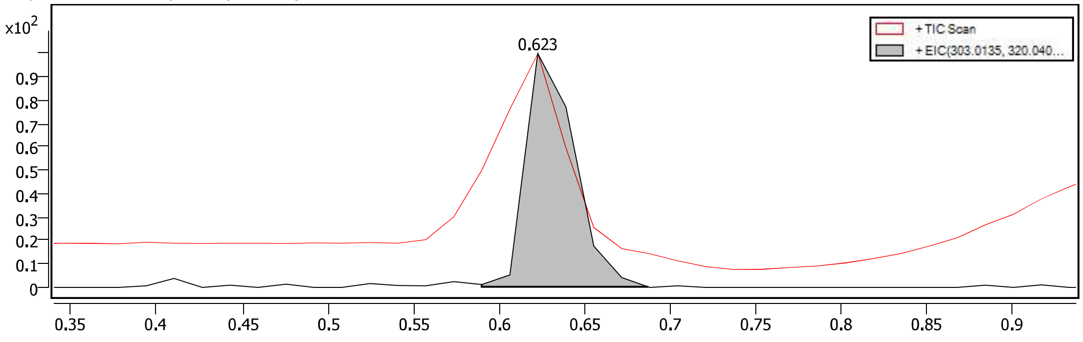

Structure

Compound Spectra (overlaid)

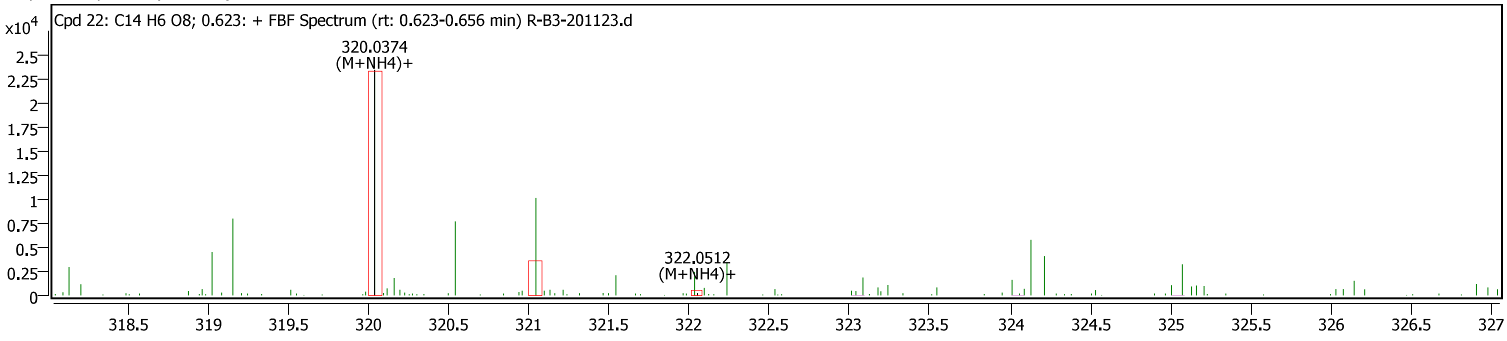

Compound ID Table

Name	Formula	Species	RT	RT Diff	Mass	CAS	ID Source	Score	Score (Lib)	Score (Tgt)
	C14 H6 O8	(M+NH4)+	0.623		302.0037		FBF	42.56		42.56

Cpd. 23: C9 H10 O5

Name	Formula	RT	RI	Mass	Diff (Tgt, ppm)	CAS	ID Source	Score	Algorithm
	C9 H10 O5	5.806		198.0522	-3.10		FBF	73.45	FBF

Species	m/z	Score (Tgt)	Score (Lib)	Score (DB)	Score (MFG)	Score (RT)
(M+H)+ (M+Na)+	199.0588 221.0438	73.45				

Compound Chromatograms (overlaid)

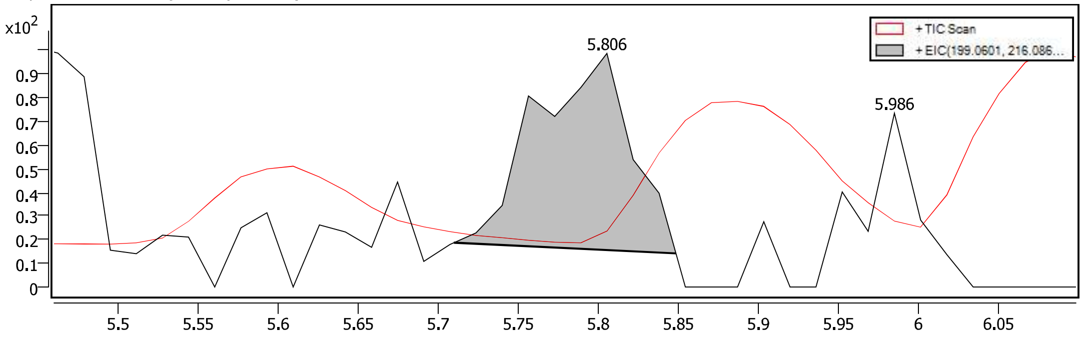

Structure

Compound Spectra (overlaid)

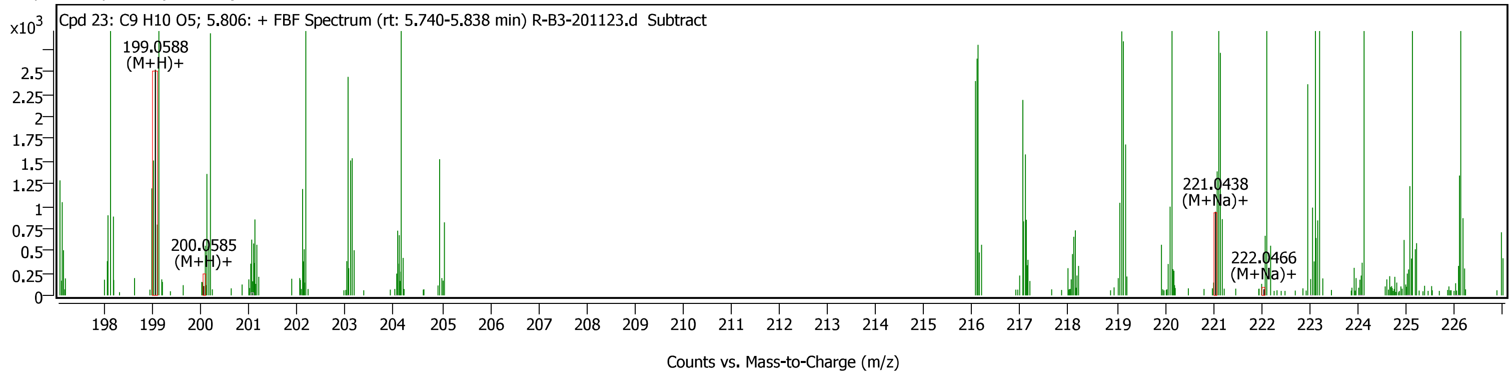

Compound ID Table

Name	Formula	Species	RT	RT Diff	Mass	CAS	ID Source	Score	Score (Lib)	Score (Tgt)
	C9 H10 O5	(M+H)+ (M+Na)+	5.806		198.0522		FBF	73.45		73.45

Cpd. 24: C10 H10 O4

Name	Formula	RT	RI	Mass	Diff (Tgt, ppm)	CAS	ID Source	Score	Algorithm
	C10 H10 O4	5.299		194.0595	8.29		FBF	75.40	FBF

Species	m/z	Score (Tgt)	Score (Lib)	Score (DB)	Score (MFG)	Score (RT)
(M+H)+ (M+NH4)+ (M+Na)+	195.0645 212.0890 217.0505	75.40				

Compound Chromatograms (overlaid)

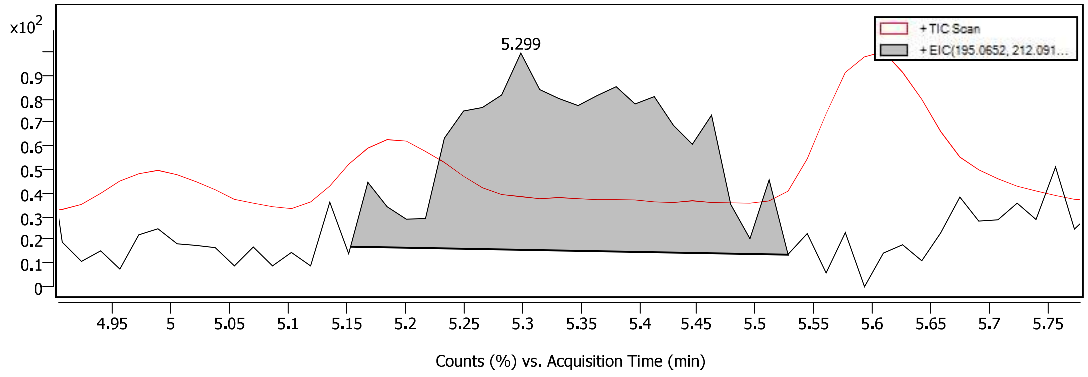

Structure

Compound Spectra (overlaid)

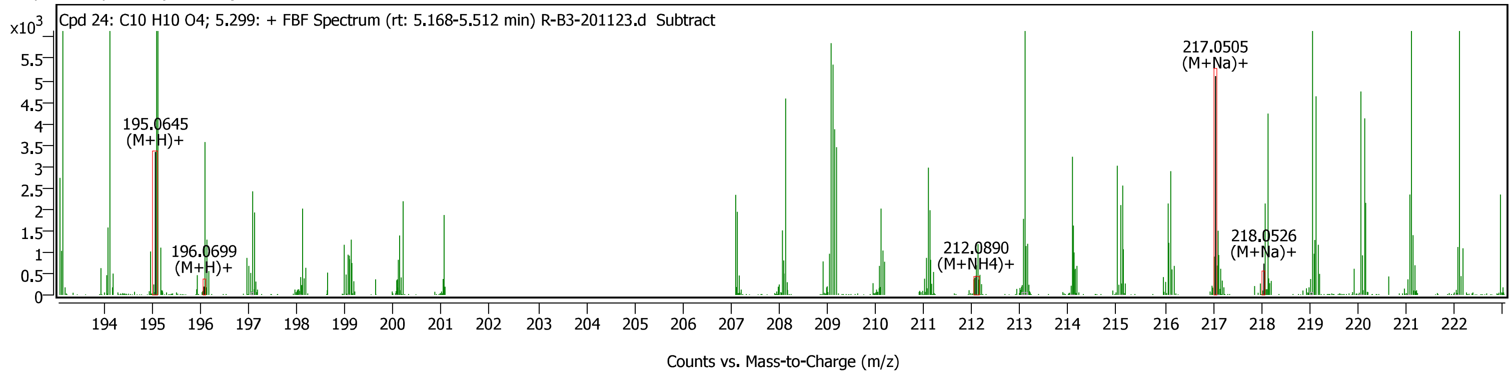

Compound ID Table

Name	Formula	Species	RT	RT Diff	Mass	CAS	ID Source	Score	Score (Lib)	Score (Tgt)
	C10 H10 O4	(M+H)+ (M+NH4)+ (M+Na)+	5.299		194.0595		FBF	75.40		75.40

Cpd. 25: C7 H6 O4

Name	Formula	RT	RI	Mass	Diff (Tgt, ppm)	CAS	ID Source	Score	Algorithm
	C7 H6 O4	3.549		154.0256	-6.63		FBF	79.18	FBF

Species	m/z	Score (Tgt)	Score (Lib)	Score (DB)	Score (MFG)	Score (RT)
(M+H)+ (M+NH4)+ (M+Na)+	155.0328 172.0580 177.0182	79.18				

Compound Chromatograms (overlaid)

Structure

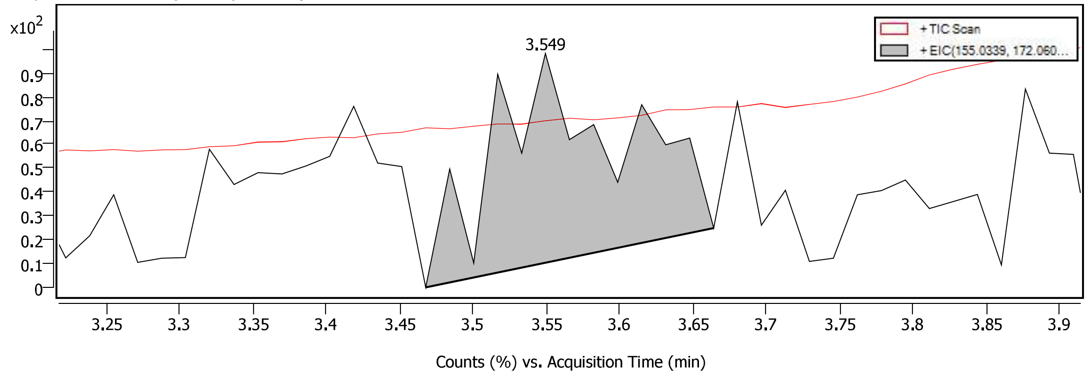

Compound Spectra (overlaid)

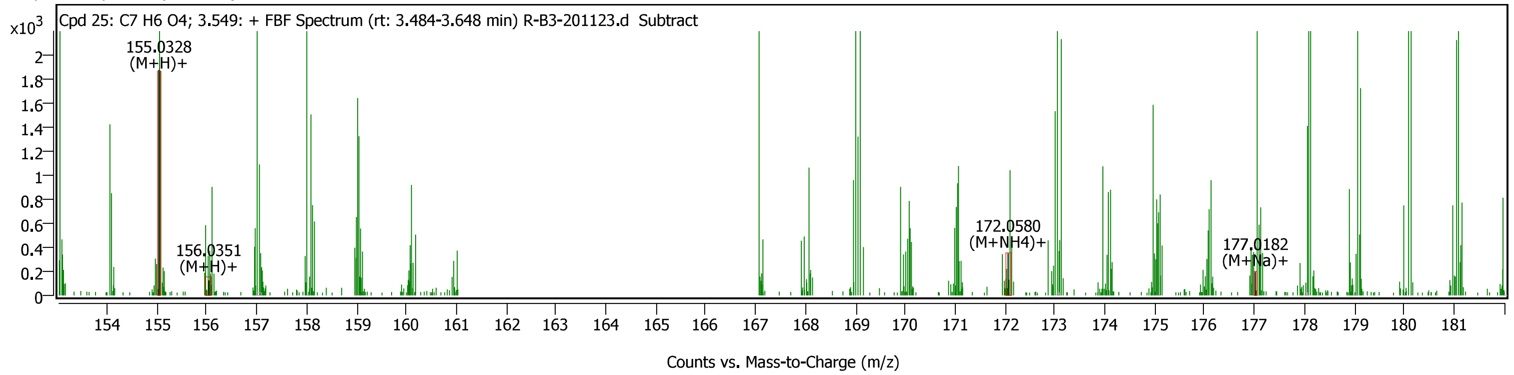

Compound ID Table

Name	Formula	Species	RT	RT Diff	Mass	CAS	ID Source	Score	Score (Lib)	Score (Tgt)
	C7 H6 O4	(M+H)+ (M+NH4)+ (M+Na)+	3.549		154.0256		FBF	79.18		79.18

Cpd. 26: C16 H14 O6

Name	Formula	RT	RI	Mass	Diff (Tgt, ppm)	CAS	ID Source	Score	Algorithm
	C16 H14 O6	0.623		302.0819	9.39		FBF	67.76	FBF

Species	m/z	Score (Tgt)	Score (Lib)	Score (DB)	Score (MFG)	Score (RT)
(M+H)+ (M+NH4)+ (M+Na)+	303.0942 320.1120 325.0717	67.76				

Compound Chromatograms (overlaid)

Structure

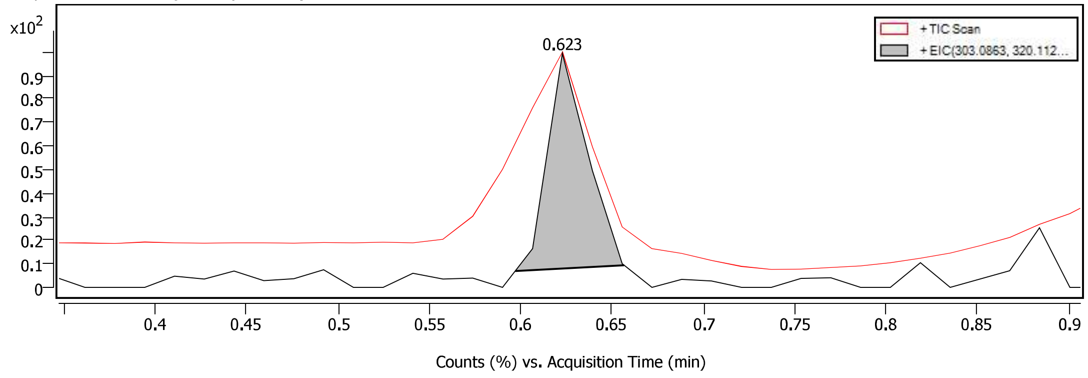

Target Screening Report

Compound Spectra (overlaid)

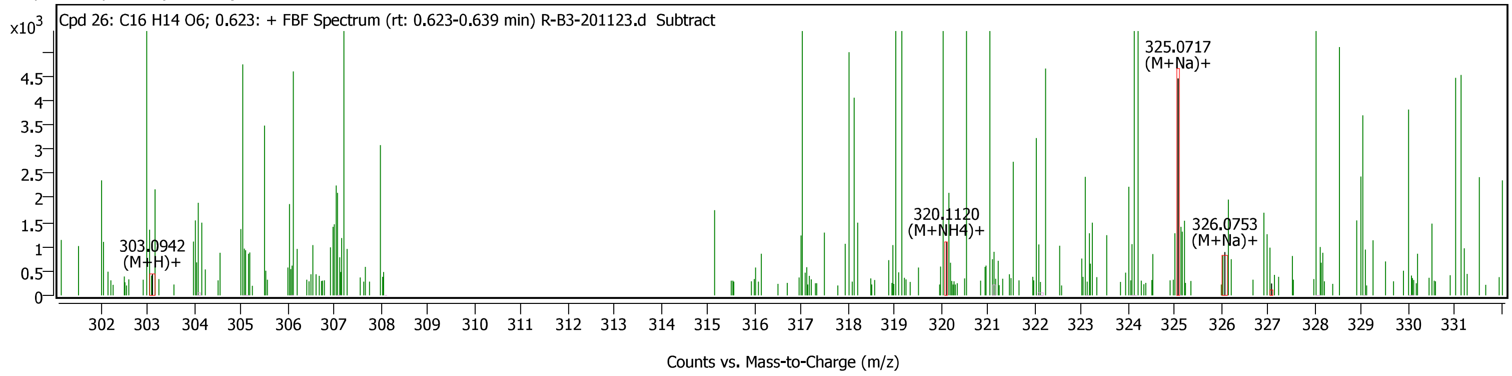

Compound ID Table

Name	Formula	Species	RT	RT Diff	Mass	CAS	ID Source	Score	Score (Lib)	Score (Tgt)
	C16 H14 O6	(M+H)+ (M+NH4)+ (M+Na)+	0.623		302.0819		FBF	67.76		67.76

Cpd. 27: C15 H10 O5

Name	Formula	RT	RI	Mass	Diff (Tgt, ppm)	CAS	ID Source	Score	Algorithm
	C15 H10 O5	6.640		270.0472	-20.90		FBF	47.61	FBF

Species	m/z	Score (Tgt)	Score (Lib)	Score (DB)	Score (MFG)	Score (RT)
(M+H)+ (M+NH4)+ (M+Na)+	271.0532 288.0862 293.0421	47.61				

Compound Chromatograms (overlaid)

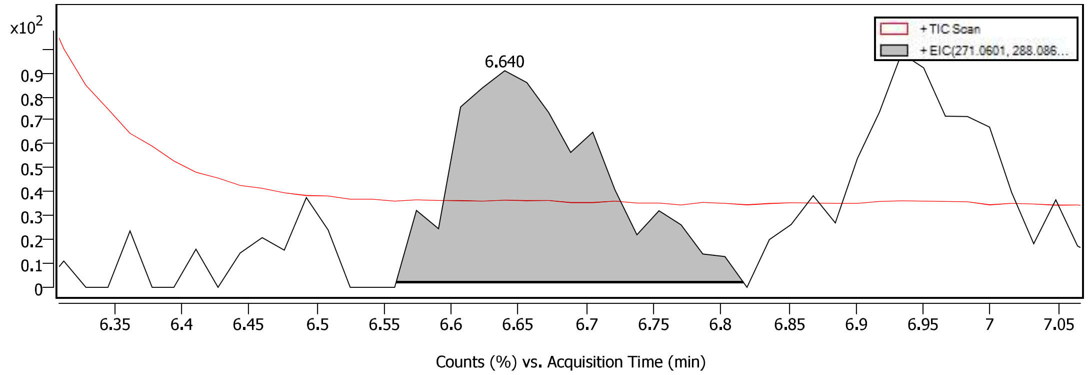

Structure

Compound Spectra (overlaid)

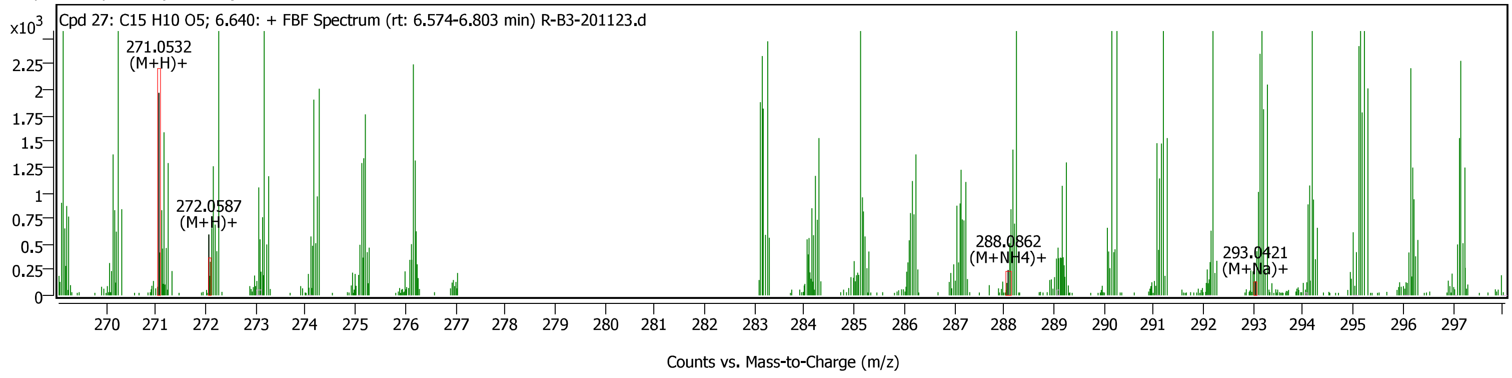

Compound ID Table

Name	Formula	Species	RT	RT Diff	Mass	CAS	ID Source	Score	Score (Lib)	Score (Tgt)
	C15 H10 O5	(M+H)+ (M+NH4)+ (M+Na)+	6.640		270.0472		FBF	47.61		47.61

Cpd. 28: C15 H14 O6

Name	Formula	RT	RI	Mass	Diff (Tgt, ppm)	CAS	ID Source	Score	Algorithm
	C15 H14 O6	5.675		290.0749	-14.25		FBF	44.05	FBF

Species	m/z	Score (Tgt)	Score (Lib)	Score (DB)	Score (MFG)	Score (RT)
(M+H)+ (M+NH4)+ (M+Na)+	291.0806 308.1118 313.0593	44.05				

Compound Chromatograms (overlaid)

Structure

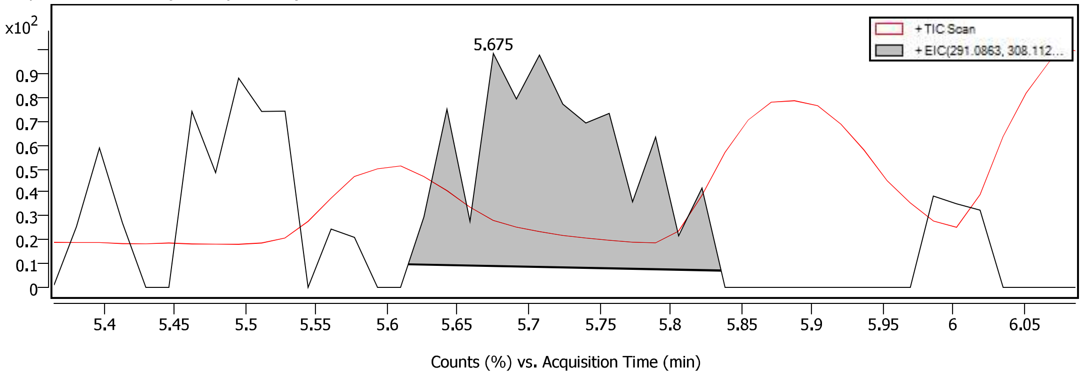

Compound Spectra (overlaid)

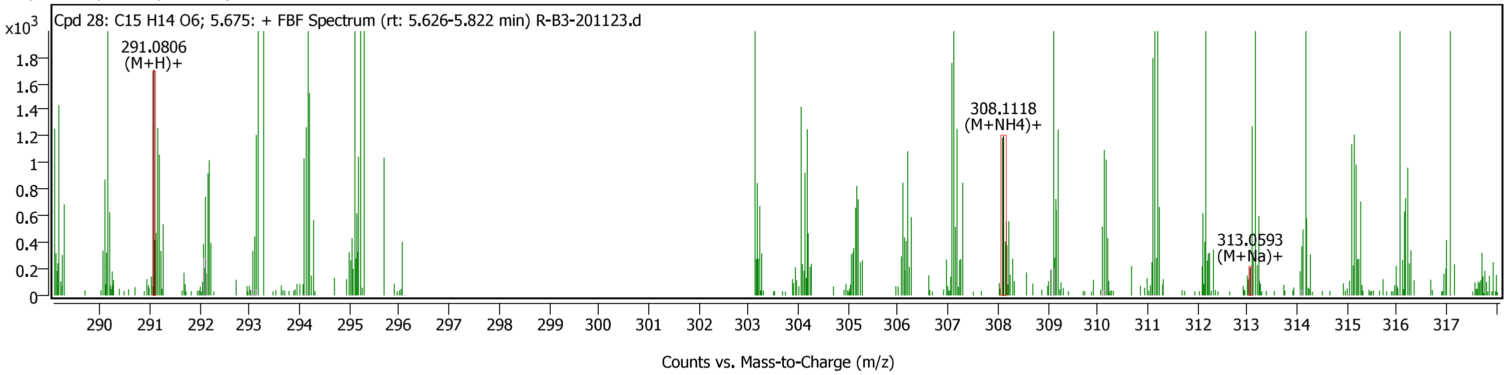

Compound ID Table

Name	Formula	Species	RT	RT Diff	Mass	CAS	ID Source	Score	Score (Lib)	Score (Tgt)
	C15 H14 O6	(M+H)+ (M+NH4)+ (M+Na)+	5.675		290.0749		FBF	44.05		44.05

Cpd. 29: C15 H10 O6

Name	Formula	RT	RI	Mass	Diff (Tgt, ppm)	CAS	ID Source	Score	Algorithm
	C15 H10 O6	5.806		286.0474	-1.18		FBF	98.82	FBF

Species	m/z	Score (Tgt)	Score (Lib)	Score (DB)	Score (MFG)	Score (RT)
(M+H)+ (M+NH4)+	287.0547 304.0765	98.82				

Compound Chromatograms (overlaid)

Structure

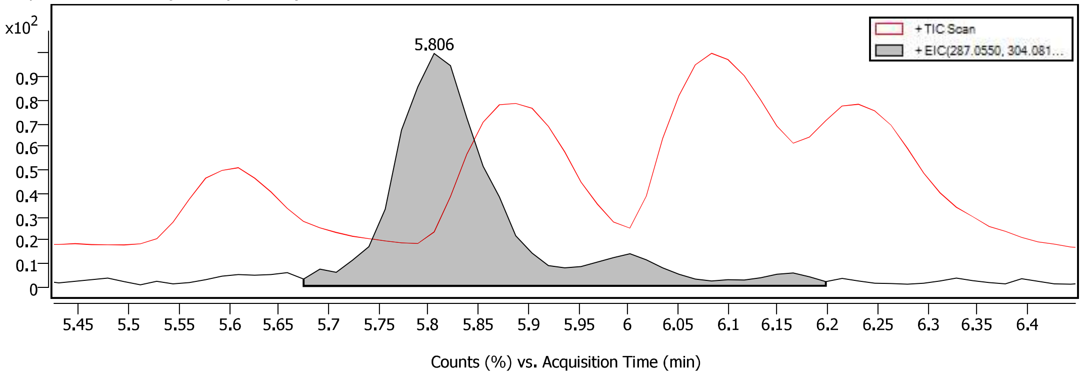

Compound Spectra (overlaid)

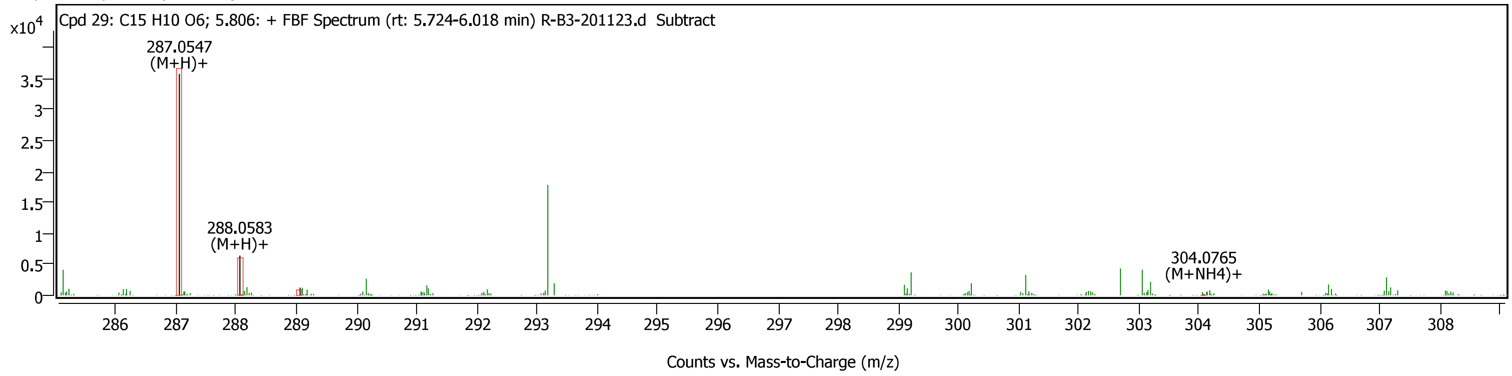

Compound ID Table

Name	Formula	Species	RT	RT Diff	Mass	CAS	ID Source	Score	Score (Lib)	Score (Tgt)
	C15 H10 O6	(M+H)+ (M+NH4)+	5.806		286.0474		FBF	98.82		98.82

Cpd. 30: C15 H10 O7

Name	Formula	RT	RI	Mass	Diff (Tgt, ppm)	CAS	ID Source	Score	Algorithm
	C15 H10 O7	5.593		302.0424	-0.94		FBF	85.20	FBF

Species	m/z	Score (Tgt)	Score (Lib)	Score (DB)	Score (MFG)	Score (RT)
(M+H)+ (M+NH4)+ (M+Na)+	303.0498 320.0747 325.0319	85.20				

Compound Chromatograms (overlaid)

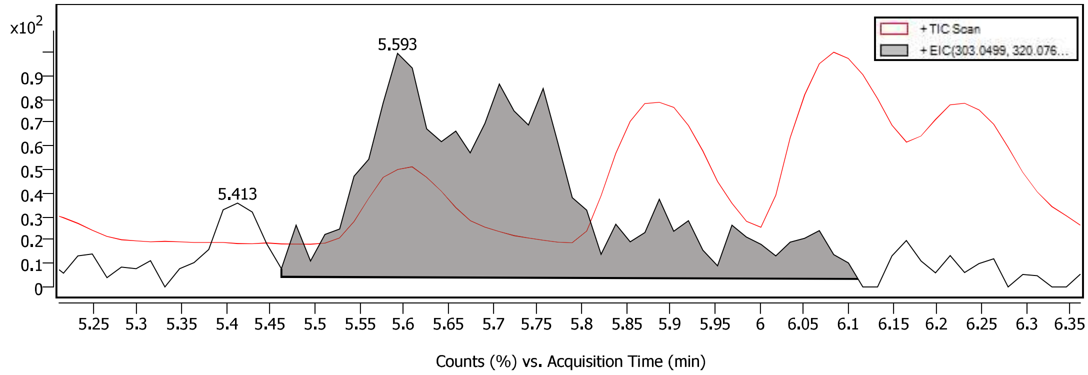

Structure

Compound Spectra (overlaid)

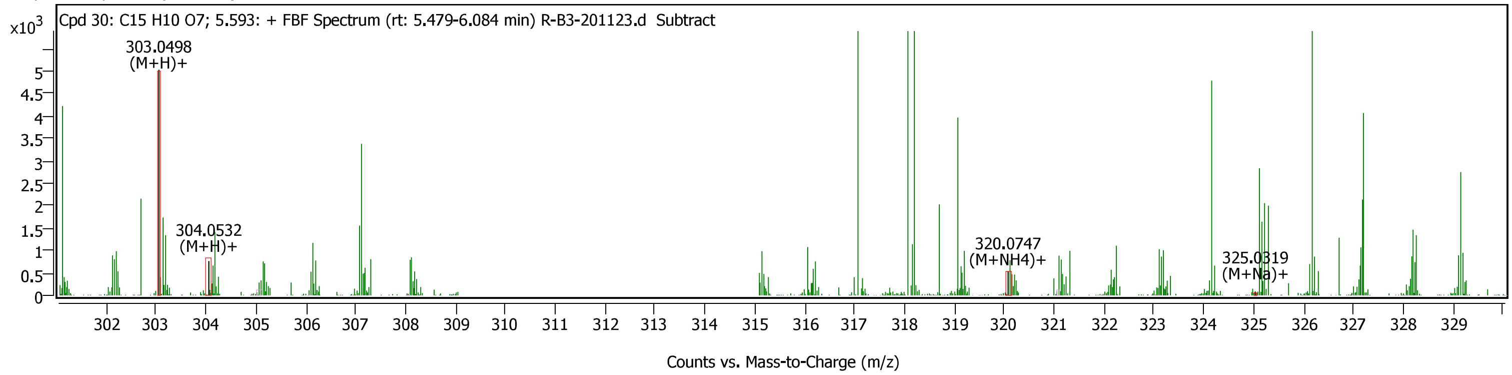

Compound ID Table

Name	Formula	Species	RT	RT Diff	Mass	CAS	ID Source	Score	Score (Lib)	Score (Tgt)
	C15 H10 O7	(M+H)+ (M+NH4)+ (M+Na)+	5.593		302.0424		FBF	85.20		85.20

MassHunter Qual 10.0
(End of Report)
